# Immunonutrition Decreases Postoperative Complications in Gastrointestinal Cancer—A Systematic Review and Meta-analysis of Randomized Controlled Trials

**DOI:** 10.1016/j.advnut.2026.100690

**Published:** 2026-06-22

**Authors:** Bettina Csilla Budai, Robert Panait, Barnabás Laczkó, Gefu Cai, Szilárd Váncsa, Dániel Sándor Veres, Karen Krisztina Fazekas, Eszter Ágnes Szalai, Krisztina Hagymási, Bálint Erőss, László Földvári-Nagy, Péter Hegyi, Stefania Bunduc

**Affiliations:** 1 Centre for Translational Medicine, Semmelweis University, Budapest, Hungary; 2 Department of Dietetics and Nutritional Sciences, Faculty of Health Sciences, Semmelweis University, Budapest, Hungary; 3 Carol Davila University of Medicine and Pharmacy, Bucharest, Romania; 4 Institute of Pancreatic Diseases, Semmelweis University, Budapest, Hungary; 5 Department of Biophysics and Radiation Biology, Semmelweis University, Budapest, Hungary; 6 Department of Restorative Dentistry and Endodontics, Semmelweis University, Budapest, Hungary; 7 Department of Surgery, Transplantation and Gastroenterology, Faculty of Medicine, Semmelweis University, Budapest, Hungary; 8 Institute for Translational Medicine, Medical School, University of Pécs, Pécs, Hungary; 9 Department of Morphology and Physiology, Faculty of Health Sciences, Semmelweis University, Budapest, Hungary; 10 Translational Pancreatology Research Group, Interdisciplinary Centre of Excellence for Research Development, and Innovation, University of Szeged, Szeged, Hungary; 11 Digestive Diseases and Liver Transplantation Center, Fundeni Clinical Institute, Bucharest, Romania

**Keywords:** immune-enhancing nutrition, immune-enhancing formula, gastrointestinal tumor, immune nutrient, perioperative nutrition

## Abstract

Current evidence supports the role of immunonutrition support (IMNS) to improve surgical outcomes in gastrointestinal (GI) cancer, but the optimal formula and prescription timing remain unclear. This systematic review and meta-analysis aimed to evaluate the efficacy of IMNS on postoperative outcomes in patients with GI cancer. The systematic search was conducted in 3 databases (PubMed, Embase, and Cochrane Library) in March 2024. The protocol was registered at PROSPERO under registration number CRD42024524537. Studies were selected based on the PICOS framework. Population: patients with GI cancer; Intervention: IMNS; Comparator: isonitrogenous and isocaloric supplementation, or standard care; Outcome: mortality, length of hospital stay, and postoperative complications; study design: randomized controlled trial (RCT). Random-effects models were used to calculate pooled odds ratios (OR) or mean differences with 95% confidence intervals (CIs). A total of 90 RCTs were included in the systematic review, with 55 eligible for meta-analysis. Data from 7462 patients were analyzed. IMNS formula based on arginine, nucleotides, and omega-3 (*n*–3) fatty acids, administered perioperatively, orally or enterally, significantly reduced the odds of anastomotic leak (OR = 0.62; 95% CI: 0.50, 0.76), infectious complications [e.g., respiratory (OR = 0.46; 95% CI: 0.33, 0.64), urinary (OR = 0.58; 95% CI: 0.38, 0.89), wound (OR = 0.67; 95% CI: 0.46, 0.98), and sepsis (OR = 0.45; 95% CI: 0.28, 0.70)], and shortened hospital stay by a mean of 2.47 d (95% CI: –4.13, –0.80). In contrast, ω-3 fatty acids alone did not improve postoperative morbidity. IMNS with arginine, nucleotides, and ω-3 fatty acids improves infection odds and hospital stay without a significant effect on overall survival, whereas ω-3 fatty acids alone did not decrease morbidity odds in patients with GI cancer undergoing surgery.

**Statement of Significance**

Immunonutrition, a formula based on arginine, nucleotides, and ω-3 fatty acids, could significantly decrease the length of hospital stay and reduce anastomotic leaks; respiratory, urinary tract, or wound infections; and sepsis odds in patients with gastrointestinal cancer undergoing surgical interventions. Immunonutrition with ω-3 fatty acids alone was not associated with reduced odds of postoperative complications in patients with gastrointestinal cancer.

## Introduction

Gastrointestinal (GI) cancer is one of the most important contributors to the global cancer burden and has an increasing incidence [1]. Surgical treatment is among the first-line therapies in early-stage disease [2]. However, malnutrition affects ≤40% of patients with GI cancer undergoing surgery [3], as multiple tumor-related factors contribute to malnutrition’s development [4]. Poor nutritional status has been associated with increased postoperative morbidity and mortality.

Immunonutrition support (IMNS), a special form of nutrition treatment, enriched with immune-modulating nutrients such as arginine (Arg), nucleotides, glutamine (Gln), omega-3 fatty acids (ω-3-FAs), vitamins and minerals, and commonly administered orally or enterally, shows promising effects in the perioperative management of patients with cancer [5]. Arg plays a key role in immune regulation, affecting immune cell biology through proinflammatory or anti-inflammatory immune response [6]. At the same time, nucleotides stimulate lymphocyte differentiation and proliferation and show anti-inflammatory effects by inhibiting TNF-α [7]. Additionally, ω-3-FAs also contribute significantly to immune functions [8].

To reduce postoperative complications, current guidelines from the European Society for Clinical Nutrition and Metabolism (ESPEN) advocate for IMNS, particularly in malnourished patients [9], whereas consensus recommendation suggests that benefits may extend irrespective of baseline nutritional status [10]. For upper GI cancer surgery, immunonutrition formulations containing Arg, ω-3-FAs, and nucleotides are strongly recommended based on high-quality evidence [11], administered primarily via oral or enteral routes [12]. There is a lack of evidence regarding the optimal timing [9,12]; however, preoperative delivery, starting 5–7 d and continuing into the postoperative phase, with daily volumes of ∼500–1000 mL, appears feasible [10].

Although IMNS has been extensively studied in upper GI surgery, evaluating its effects across GI malignancies is clinically justified. GI cancers share key features, including a high prevalence of malnutrition, systemic inflammation, and the need for major abdominal surgery, all of which substantially influence postoperative outcomes. Nevertheless, the current evidence base remains fragmented across tumor sites, limiting the generalizability of findings and the ability to determine whether observed treatment effects are primarily related to tumor-specific characteristics or reflect shared perioperative and inflammatory mechanisms. Furthermore, the optimal composition and administration strategies for IMNS in major oncologic surgical settings remain insufficiently defined [9,12].

Recent meta-analyses examined the effects of IMNS in patients undergoing surgery for GI and head and neck cancer [[13], [14], [15]]. These studies have reported favorable effects on various postoperative outcomes. Although some addressed the impact of timing and cancer type [13,14], data on specific IMNS formulations are limited. Only the presence or absence of Arg has been evaluated separately [13,14], whereas other component-specific comparisons are lacking [15].

The present study aimed to investigate the efficacy of oral or enteral IMNS in reducing postoperative morbidity and mortality in patients with GI cancer. We aimed to perform relevant subgroup analyses by cancer type, IMNS formula, and timing of IMNS to better understand the opportunities for IMNS personalization in patients with GI cancer.

## Methods

We report our systematic review and meta-analysis based on the recommendations of the PRISMA 2020 guideline [16] (Supplemental Table 1) and the Cochrane Handbook [17]. The protocol for our study was previously registered with the PROSPERO under registration number CRD42024524537. We had 2 protocol deviations; only randomized controlled trial (RCT) articles were included to ensure the highest quality evidence, and we excluded parenteral nutrition as an intervention from our review. The research project is conducted under the Systems Education model coordinated by the Centre of Translational Medicine at Semmelweis University and the Hungarian Pancreatic Study Group [18,19] and conducted within the translational medicine Cycle Framework by the Academia Europaea [20].

### Eligibility criteria

We included RCTs that met the following eligibility criteria, based on the PICOS framework: the population (P) was adult patients (≥18 y) diagnosed with GI cancer, the intervention (I) was IMNS as defined by the individual study protocols, and the comparator (C) was either a placebo or standard care without IMNS. No restrictions were imposed regarding the specific composition of the IMNS formula. The primary outcomes (O) included in-hospital mortality and long-term mortality, assessed at different follow-up intervals. The secondary outcomes were postoperative morbidity and biomarkers related to immune function. In studies that compared multiple IMNS administration timings, only data from the preoperative administration groups were included in the primary meta-analysis. Postoperative data were extracted but incorporated exclusively in subgroup analyses examining the timing of administration. When studies involved >1 comparator group, the group receiving a placebo without nutritional supplementation was selected for inclusion in the primary analysis.

Articles on neuroendocrine tumors, primary cancers outside the GI tract, or having pediatric populations were excluded.

### Search strategy

Our systematic search was conducted on the 22 March, 2024 in 3 databases: PubMed, Embase, and the Cochrane Library, without time or language restrictions. The search key included 3 domains comprising synonym terms related to the “GI tract,” “cancer,” and “immunonutrition,” respectively. The complete search key is found in the Supplemental Document 1.

### Selection process

Two pairs of independent reviewers (BCB and BL; BCB and RP) first screened the studies by title and abstract, then by full-text content, using the Rayyan software [21]. Cohen’s kappa coefficient was calculated after each selection step to assess interrater agreement, with discrepancies resolved by a third reviewer (RP or BL). During the selection process, we also screened the references of eligible papers for further relevant reports. All included articles are available in full text and were published in peer-reviewed journals.

### Data collection process

Data extraction was performed independently by 2 authors (RP and BL) and checked by a third investigator (BCB) to ensure quality. A standardized, predesigned data collection sheet was used to collect study-level information, including author, year of publication, design, country of origin, population characteristics (sample size, age, sex, type of cancer treatment), and intervention details (composition of IMNS, product name, route of administration, dosage, and timing of IMNS).

We extracted all data relevant to our predefined outcomes, including mortality as the primary outcome and secondary outcomes such as postoperative adverse events, length of hospital stay (LOH), and serum markers of inflammation. When mortality was reported at multiple time points, we extracted all available results to enable subgroup analyses based on follow-up duration. Similarly, when biomarker outcomes were reported at different time points, we extracted all data to examine temporal changes. For dichotomous outcomes, we extracted the number of events in intervention and control groups, or odds ratios (ORs) with corresponding 95% confidence intervals (CIs) if reported. For continuous outcomes such as hospital stay and biomarkers, we extracted means with SDs, or medians with IQRs when means and SDs were unavailable.

### Synthesis methods

Random-effects models were applied to calculate pooled effect estimates due to the anticipated heterogeneity among studies. The pooled effect size was expressed as OR with the corresponding 95% CIs for dichotomous variables, whereas for continuous variables, we reported mean differences (MD) with 95% CIs.

The Mantel–Haenszel method was applied to calculate the OR based on the extracted data. We reported the results as the odds of an event in patients who received IMNS, compared with the odds in the control group. A Hartung–Knapp adjustment [[22], [23], [24], [25]] was applied for the CI calculation of pooled OR (also for calculating *t*-test-based *P* value).

MDs were derived using the extracted sample sizes, means, and SDs from each group separately. We reported the results as experimental group minus control group values.

Between-study variance (τ^2^) was estimated using the Paule–Mandel method [26], and corresponding CIs were calculated using the Q profile method [27]. Heterogeneity was additionally evaluated with Higgins and Thompson’s *I*^2^ statistic [28].

Forest plots were used to visualize the pooled estimates, and statistical significance was determined based on whether the CIs did not contain the null value. Where applicable, the study number was sufficiently large (number of studies >7) and not too heterogeneous; prediction intervals were also calculated (i.e., the expected range of true effects in future studies) for the results.

Several subgroup analyses were performed. A fixed-effects (plural) model was used to analyze subgroups, assuming the same τ^2^ across all subgroups [29].

To investigate how the effect size varies with follow-up time, we conducted meta-regression analyses. We assumed a linear relationship and specified an autoregressive correlation structure of order 1 [AR(1)] with an initial correlation coefficient of 0.7, applying a robust sandwich variance estimator to account for potential misspecification. Sensitivity analyses were performed using different initial correlation values, a symmetric correlation structure, and models without the robust estimator.

We evaluated potential publication bias by visual inspection of funnel plots and Egger’s test [30] for analyses of a minimum of 10 studies reporting the outcome of interest. Sensitivity analyses were performed in accordance with recommendations by Harrer et al. [31].

All statistical analyses were conducted using *R* software [32], employing the *meta* package [33] for basic meta-analysis calculations and visualizations, and the *dmetar* package [34] for additional influential analysis calculations and plots. The metaregression was performed using the *metafor* package [35] with the *clubSandwich* [36] package for a robust estimator.

### Study risk of bias assessment and certainty of evidence

Risk of bias assessment was performed by 2 pairs of authors (BCB and GC; BL and RP) independently using the ROB-2 tool [37]. Disagreements were solved by a third person (BCB or BL). We used the RobVis tool for visualization [38].

We assessed the certainty of evidence according to the Grading of Recommendations Assessment, Development, and Evaluation (GRADE) [39].

## Results

### Search and selection

**FIGURE 1:**
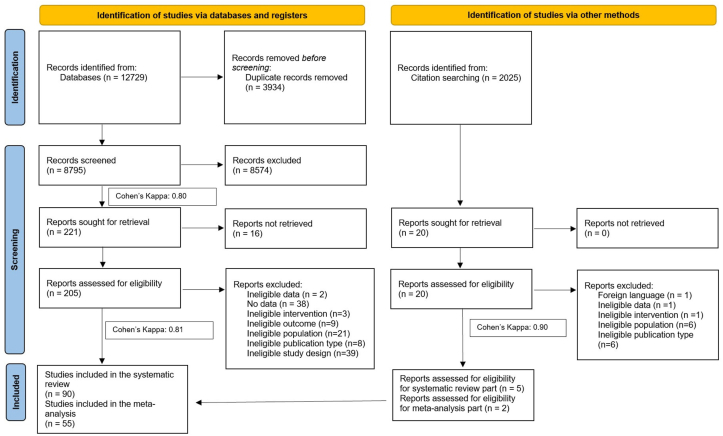
PRISMA flowchart of the selection.

The result of the selection process is summarized in Figure 1. We identified 12,729 reports. We included 90 articles in the systematic review and 55 [[40], [41], [42], [43], [44], [45], [46], [47], [48], [49], [50], [51], [52], [53], [54], [55], [56], [57], [58], [59], [60], [61], [62], [63], [64], [65], [66], [67], [68], [69], [70], [71], [72], [73], [74], [75], [76], [77], [78], [79], [80], [81], [82], [83], [84], [85], [86], [87], [88], [89], [90], [91], [92], [93], [94]] in the meta-analysis. In some of the studies, the IMNS formula was not mentioned [[95], [96], [97], [98], [99]], whereas others used unique IMNS formulas [[100], [101], [102], [103], [104], [105], [106], [107], [108], [109], [110], [111], [112], [113], [114], [115]]. Some studies had multiple reports; therefore, some articles were excluded from our systematic review [[116], [117], [118], [119], [120], [121], [122], [123], [124], [125], [126], [127], [128], [129]]. If multiple publications reported results from the same trial, the study including the largest patient population was used. When several comparator groups were available within a study, the placebo or standard-care control group was selected for meta-analysis. The complete strategy for avoiding overlapping populations is detailed in Supplemental Document 2.

### Basic characteristics of included studies

The study characteristics are summarized in Supplemental Table 2. The prevalence of malnourished patients varied across the included studies. In most of the studies, malnutrition prevalence was measured by screening tools, whereas others only reported the number of patients experiencing more than 5%–10% weight loss or had a BMI <19 kg/m^2^. The enteral route varied, including jejunostomy, gastrostomy, and tube feeding via nasogastric, nasoduodenal, or nasojejunal routes. Many studies investigated upper GI tract and GI cancer together, as well as specific cancer types. Regarding the IMNS formula, the combination of Arg, nucleotides, and ω-3-FAs (Arg+nucleotides+ω-3-FAs) was the most commonly used, whereas the doses of the immunonutrition were mixed (Supplemental Table 3).

### Effect of IMNS on mortality in patients with GI cancer

**FIGURE 2:**
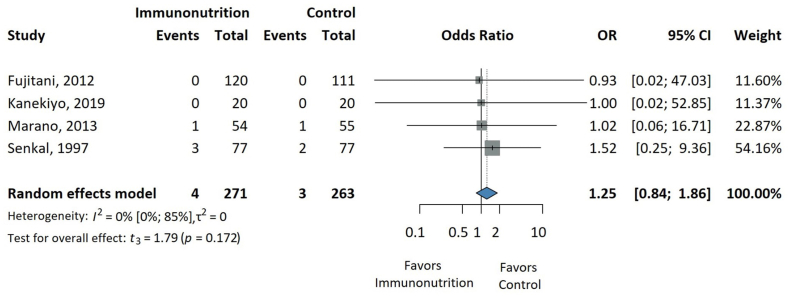
Meta-analysis of the effect of immunonutrition support (Arg+nucleotides+ω-3-FAs) on in-hospital mortality in gastrointestinal cancer. Arg+nucleotides+ω-3-Fas, arginine + nucleotides, omega-3 fatty acids; OR, odds ratio; 95% CI, 95% confidence interval.

Most studies reported mortality at several time points, including in-hospital, 30-d, 1-y, or beyond-1-y mortality after surgery (alone or in combination with other oncologic treatments). Most studies reporting on mortality used the standard IMNS based on Arg+nucleotides+ω-3-FAs. IMNS did not improve in-hospital mortality in patients with GI cancer when administered perioperatively (OR = 1.25, 95% CI: 0.84, 1.86, 4 studies [54,64,75,85], 534 patients) (Figure 2), and it did not show a significant effect on 30-d mortality (OR = 0.78, 95% CI: 0.39, 1.54, 6 studies [40,44,47,59,68,79], 523 patients) (Supplemental Figure 1). The mixed formulas (Arg+Gln+ω-3-FAs, Arg+Gln, Arg+nucleotides+ω-3-FAs, and Arg,+ω-3-FAs+dietary fiber) did not significantly change 30-d mortality (OR = 0.85, 95% CI: 0.55, 1.32, 8 studies [40,41,44,59,67,68,79,94], 814 patients) (Supplemental Figure 2) or long term survival (1-y - OR = 1.02, 95% CI: 0.60, 1.73, 3 studies [41,66,72], 315 patients; 3-y survival – OR = 1.33, 95% CI: 0.49, 3.64, 3 studies [66,70,72], 285 patients; 5-y OS - OR = 1.01, 95% CI: 0.37, 2.77, 3 studies [41,64,66], 247 patients) (Supplemental Figures 3–5).

The formulas were administered mainly postoperatively in the studies analyzing the long-term effect, except in 2 cases when the administration was perioperative [64] or after 1 course of chemotherapy [70].

Evidence for 1-y overall mortality was judged to be of low certainty due to inconsistency, indirectness, and imprecision. Furthermore, evidence on in-hospital mortality shows a moderate level of certainty due to indirectness (Supplemental Table 5).

Due to insufficient data for meta-analysis, we summarized progression-free survival results narratively. Kanekiyo et al. [64] reported that 75% of Arg+nucleotides+ω-3-FA patients experienced 5-y progression-free survival, compared with 64% in the control group. Shibata et al. [130] reported a 3-y progression-free survival rate of 97.4% in patients receiving an Arg-enhanced diet, compared with 85.5% in the control group. No significant difference was recorded in the study by Luo et al. [114] between the intervention group (Arg+nucleotides+ω-3-FA with intravenous Gln and vitamin complex) and the control group.

### Effect of perioperative IMNS on postoperative morbidity in patients with GI cancer undergoing surgical interventions

Infectious complications odds decreased overall with Arg+nucleotides+ω-3-FA administered perioperatively (OR = 0.51, 95% CI: 0.32, 0.81, 14 studies [45,48,52–54,58,59,64,74,75,80,82,83,90], 2390 patients) (Supplemental Figure 6); however, we did not find significant effects at subgroup analysis by time of administration (preoperative – OR = 0.68, 95% CI: 0.40, 1.16, 9 studies [48,52,54,59,74,80,82,83,90], 1259 patients; postoperative – OR = 0.60, 95% CI: 0.26, 1.37, 5 studies [45,53,75,82,90], 1209 patients Supplemental Figures 7 and 8). The certainty of evidence for infectious complications was rated as very low, reflecting serious concerns regarding inconsistency, indirectness, and potential publication bias.

Perioperative administration of Arg+nucleotides+ω-3-FAs was associated with reduced rates of respiratory, urinary tract, and wound infections, as well as sepsis, but not surgical site infections. These results are supported by moderate-certainty evidence (Table 1). Individual plots are found in the Supplemental Material in Supplemental Figures 9–20.

**TABLE 1:** Effect of immunonutrition support (Arg+nucleotides+ω-3-FAs) on infectious complications

Outcome	Administration time	Cancer type	Number of studies	Number of patients (intervention vs. control group)	OR with 95% CI	GRADE assessment
Respiratory infection∗	Perioperative∗	GI	7	290 vs. 293	0.46 (0.33, 0.64)∗	⊕⊕⊕◯ Moderate
	Preoperative∗	GI	5	214 vs. 218	0.50 (0.29, 0.84)∗	⊕⊕⊕◯ Moderate
Urinary tract infection∗	Perioperative	Upper GI	4	227 vs. 223	0.61 (0.14, 2.71)	⊕⊕⊕◯ Moderate
	Perioperative∗	GI	14	822 vs. 814	0.58 (0.38, 0.89)∗	⊕⊕⊕◯ Moderate
	Preoperative	GI	9	452 vs. 443	0.63 (0.35, 1.14)	⊕⊕⊕◯ Moderate
	Postoperative	GI	5	313 vs.311	0.61 (0.21, 1.76)	⊕⊕⊕◯ Moderate
Wound infection∗	Perioperative∗	GI	16	788 vs. 755	0.67 (0.46, 0.98)∗	⊕⊕⊕◯ Moderate
	Perioperative	Gastric	4	239 vs. 230	0.56 (0.23, 1.36)	⊕⊕⊕◯ Moderate
	Preoperative	GI	12	595 vs. 563	0.71 (0.44, 1.15)	⊕⊕⊕◯ Moderate
	Postoperative	GI	4	236 vs. 234	0.75 (0.20, 2.74)	⊕⊕⊕◯ Moderate
Sepsis∗	Perioperative∗	GI	9	528 vs.520	0.45 (0.28, 0.70)∗	⊕⊕⊕◯ Moderate
	Perioperative∗	Upper GI	4	227 vs. 223	0.36 (0.17, 0.77)∗	⊕⊕⊕◯ Moderate
	Perioperative∗	GI	6	367 vs. 358	0.43 (0.22, 0.87)∗	⊕⊕⊕◯ Moderate
	Perioperative∗	GI	4	226 vs. 224	0.43 (0.27, 0.69)∗	⊕⊕⊕◯ Moderate
Surgical site infection	Perioperative	GI	7	398 vs. 383	0.71 (0.33, 1.53)	⊕⊕⊕◯ Moderate

Individual plots are found in the Supplemental Material in Figures 9–20.

Abbreviations: Arg, arginine; CI, confidence interval; GI, gastrointestinal; GRADE, Grading of Recommendations Assessment, Development, and Evaluation; OR, odds ratio; ω-3-FAs, omega-3 fatty acids.

∗ Statistically significant outcomes and risks are indicated.

No significant effect was observed on noninfectious complications (OR = 0.94, 95% CI: 0.80, 1.11, 10 studies [45,48,52,58,59,62,80,82,83,90], 1890 patients), regardless of timing of administration (preoperative – OR = 0.93, 95% CI: 0.75, 1.16, 8 studies [48,52,59,62,80,82,83,90], 968 patients; postoperative – OR = 1.03, 95% CI: 0.58, 1.83, 3 studies [45,82,90], 1040 patients (Supplemental Figures 21–23). Definitions of noninfection complications varied across studies; they are summarized in Supplemental Document 3 and include cardiac dysfunction, respiratory dysfunction, anastomotic leak, etc.

Ω-3-FAs alone did not reduce urinary tract infection (OR = 1.53, 95% CI: 0.68, 3.43, 3 studies [42,88,89], 297 patients) or sepsis odds when administered (OR = 1.88, 95% CI: 1.24, 2.85, 4 studies [42,63,88,89], 488 patients) (Supplemental Figures 24 and 25).

**FIGURE 3:**
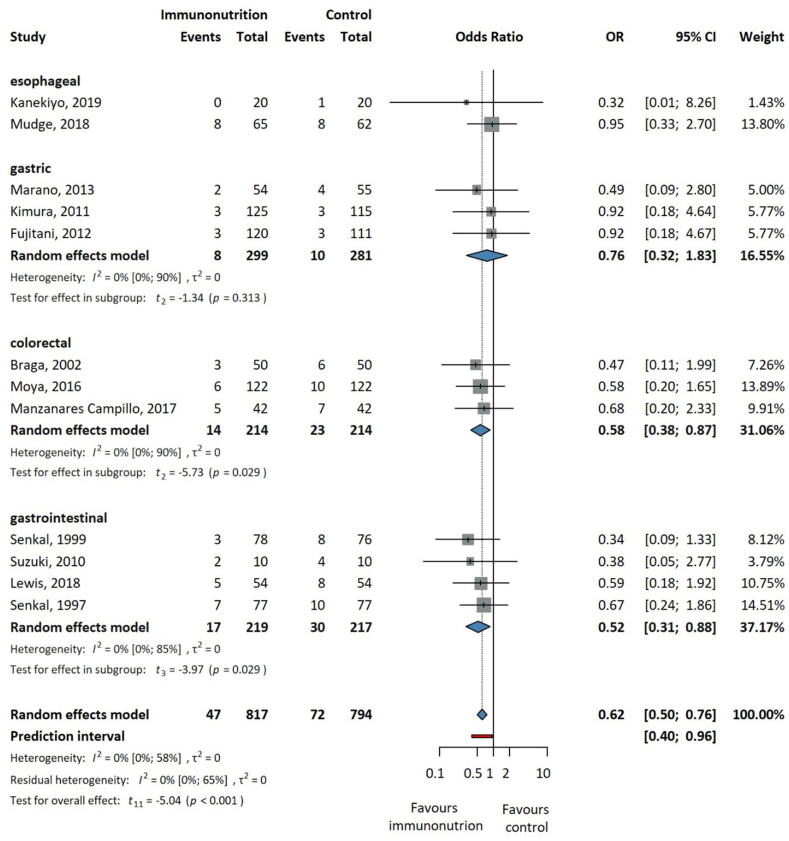
Meta-analysis of the effect of immunonutrition support (Arg+nucleotides+ω-3-FAs) on anastomotic leak in gastrointestinal cancer. Arg+nucleotides+ω-3-Fas, arginine + nucleotides, omega-3 fatty acids; OR, odds ratio; 95% CI, 95% confidence interval.

Overall, odds of anastomotic leakage are decreased by perioperative Arg+nucleotides+ω-3-FAs (OR = 0.62, 95% CI: 0.50, 0.76, 12 studies [47,54,64,65,68,74,75,81,82,85,86,90], 1611 patients) (Figure 3). The data were limited for subgroup analysis by cancer type. The odds were decreased in colorectal cancer (OR = 0.58, 95% CI: 0.38, 0.87, 3 studies [47,74,81], 428 patients), but not changed in gastric cancer (OR = 0.76, 95% CI: 0.32, 1.83, 3 studies [54,65,75], 580 patients) or in esophageal cancer (OR = 0.86, 95% CI: 0.01, 49.22, 2 studies [64,82], 167 patients).

Subgroup analysis based on time of administration showed significant reductions with preoperative delivery (OR = 0.61, 95% CI: 0.42, 0.87, 8 studies [48,54,65,68,74,82,86,90], 1168 patients), whereas the results of postoperative administration remain uncertain due to data scarcity (OR = 1.52, 95% CI: 0.03, 84.96, 3 studies [75,82,90], 256 patients) Supplemental Figures 26 and 27) Ω-3-FAs alone did not reduce the odds of anastomotic leakage patients (OR = 2.90, 95% CI: 0.31, 26.70, 5 studies [63,73,77,88,89], 605 patients, Supplemental Figure 28). The certainty of evidence for anastomotic leakage was rated as moderate due to indirectness.

### Effect of IMNS on the LOH stay in patients with GI cancer undergoing surgical interventions

**FIGURE 4:**
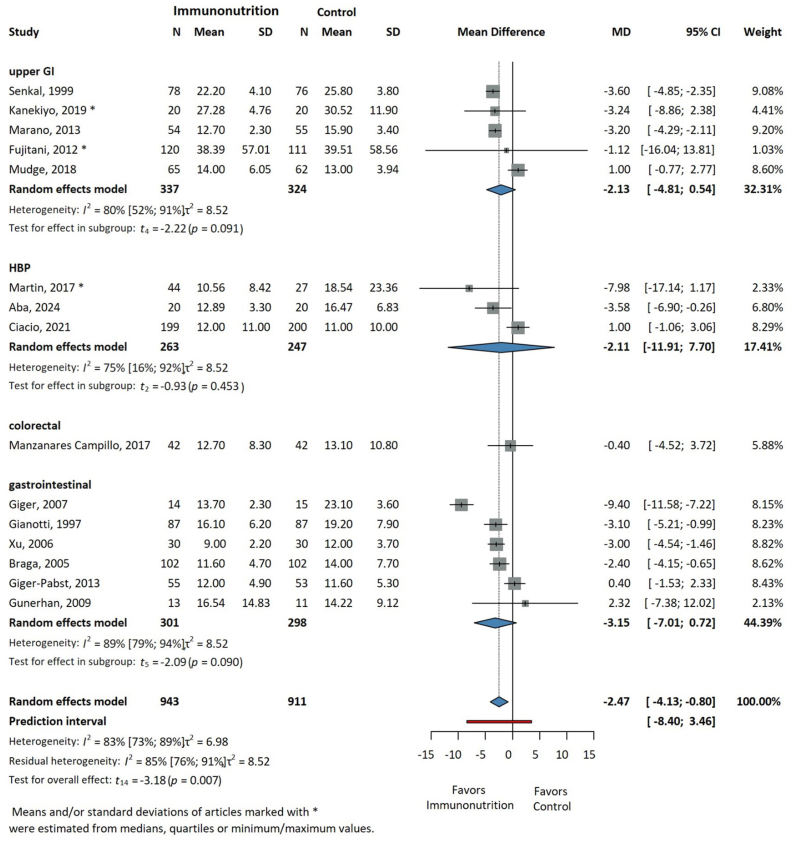
Meta-analysis of the effect of immunonutrition support (Arg+nucleotides+ω-3-FAs) on length of hospital stay (d) in gastrointestinal cancer. Arg+nucleotides+ω-3-Fas, arginine + nucleotides, omega-3 fatty acids; MD, mean difference; N, number of patients; 95% CI, 95% confidence interval.

Perioperative Arg+nucleotides+ω-3-FA reduces the LOH by an mean of 2 d, in patients with GI cancer undergoing curative intent resection (MD = –2.47, 95% CI: –4.13, –0.80, 15 studies [40,48,52,54,57–59,62,64,74–76,82,86,92], 1854 patients) (Figure 4). Data for subgroup analysis based on cancer type were limited.

Both preoperative (MD = –1.42, 95% CI: –2.95, –0.10, 11 studies [40,48,52,54,59,62,74,76,82,86,92], 1502 patients) and postoperative (MD = –3.16, 95% CI: –3.79, –2.52, 2 articles, 283 patients) delivery showed a clear tendency to decrease LOH (Supplemental Figure 29).

The evidence for the LOH reveals a low level of certainty due to serious concerns regarding inconsistency and imprecision.

We conducted a metaregression to evaluate changes in serum biomarkers associated with immune function after IMNS. Overall, IMNS tended to decrease inflammation; however, the changes in inflammatory parameters were not statistically significant and were primarily clinically irrelevant. The results are summarized in Table 2. Arg+nucleotides+ω-3-FA was associated with a modest, nonsignificant reduction in C-reactive protein (CRP) levels over time, with a decrease of 1.6 mg/L across a 1 to 30-d follow-up. ω-3-FAs alone demonstrated a greater, yet still statistically nonsignificant, reduction in CRP (12.2 mg/L) over a shorter follow-up of 1 to 8 d. IL-6 serum level decreased by 10.9 pg/mL within 30 d with Arg+nucleotides+ω-3-FA, whereas ω-3-FAs were associated with a greater IL-6 reduction of 134.50 pg/mL over 10 d.

**TABLE 2:** Results of meta-regression analysis of the association between immunonutrition support and serum biomarkers

Serum parameter	IMNS type	Treatment duration (d)	Slope	Slope SE	Intercept	*P* value
Albumin	Arg+nucleotides+ω-3-FAs	8	0.20 g/L/d	0.14 g/L/d	–0.76	0.2235
CD4+/CD8+ ratio	Arg+nucleotides+ω-3-FAs	14	0.04	0.03	0.24	0.3377
CRP	Arg+nucleotides+ω-3-FAs	30	–0.16 mg/L/d	0.84 mg/L/d	–23.12	0.8611
CRP	ω-3-FAs	8	–1.22 mg/L/d	2.71 mg/L/d	–1.48	0.6964
IL-6	Arg+nucleotides+ω-3-FAs	30	–1.09 pg/mL/d	3.18 pg/mL/d	–39.88	0.7686
IL-6	ω-3-FAs	10	–13.45 pg/mL/d	21.20 pg/mL/d	–39.81	0.5908
Total lymphocytes	Arg+nucleotides+ω-3-FAs	14	–16.67 cells/mm^3^/d	24.44 cells/mm^3^/d	–26.13	0.5506
Prealbumin	Arg+nucleotides+ω-3-FAs	14	0.003 g/L/d	0.002 g/L/d	–0.001	0.1266
Transferrin	Arg+nucleotides+ω-3-FAs	8	–0.31 mg/dL/d	0.84 mg/dL/d	9.91	0.7292
WBC	ω-3-FAs	30	–5.41 10^9^/L/d	60.60 10^9^/L/d	–229.61	0.9422

Abbreviations: Arg+nucleotides+ω-3-Fas, arginine + nucleotides, ω-3 fatty acids; CRP, C-reactive protein; IMNS, immunonutrition support; se, serum; WBC, white blood cell. CD, cluster of differentiation.

No significant effects were observed on albumin, prealbumin, total lymphocyte count, transferrin, or CD4+/CD8+ ratio with Arg+nucleotides+ω-3-FA. The complete outputs of the meta-regression analysis are provided in Supplemental Document 4.

In studies that reported cancer stage explicitly, subgroup analyses were conducted by disease stage. Perioperative IMNS containing Arg+nucleotides+ω-3-FAs was associated with a significantly reduced odds of anastomotic leakage in populations including patients with stage I–IV cancer. In contrast, this significant association was not observed in studies restricted to stage I–III disease. A similar pattern was identified for infectious complications, with significantly lower odds observed after perioperative Arg+nucleotides+ω-3-FA supplementation in stage I–IV populations (Supplemental Figures 30 and 31). No statistically significant subgroup differences according to cancer stage were detected for the remaining outcomes (Supplemental Figures 32–38).

### Biases and certainty of evidence

The overall results of the risk of bias assessment are presented in Supplemental Table 4. Across most domains, including the randomization process, deviations from intended interventions, missing outcome data, and outcome measurement, the majority of studies were judged to be at low risk of bias. Some studies showed minor concerns, particularly in outcome measurement and selective reporting, whereas only a very small proportion were assessed as high risk.

The certainty of evidence ranged from very low to moderate (Supplemental Table 5). The low certainty of evidence reflects concerns regarding inconsistency, indirectness, potential publication bias, and imprecision.

In most analyses, no publication bias was detected; however, in the analysis of infectious complications by cancer type, asymmetry was observed in the funnel plots (Egger test: *t* = –3.61, df = 12, *P* value = 0.0036). Specifically, studies with smaller sample sizes tended to report lower ORs, whereas the opposite side of the funnel plot was absent, indicating possible selective publication or reporting bias. The funnel plots are found in the Supplemental Figures 39–45.

Leave-one-out sensitivity analysis identified potential outliers in the cases of noninfection complications and LOH, suggesting nonrobust results. The results of the detailed leave-one-out analysis are found in the Supplemental Figures 46–52.

Additionally, subgroup analysis according to IMNS based on ω-3-FAs alone was underpowered due to small sample sizes and wide CIs.

## Discussion

This meta-analysis evaluated the efficacy of IMNS in patients with GI cancer. Although it did not seem to reduce in-hospital mortality or short and long-term mortality, IMNS significantly decreased postoperative complications, like infections, anastomotic leakage, and LOH.

Data scarcity did not allow for comprehensive subgroup analyses by time of administration or formula type. However, within the available data, preoperative IMNS was associated with more favorable outcomes than postoperative IMNS, particularly in reducing anastomotic leakage and respiratory infection. This result aligns with the ESPEN guideline, which recommends the perioperative use of IMNS, especially in patients with upper GI cancer [11]. Notably, our results extend these recommendations by underscoring the potential added benefit of preoperative administration, especially since the current ESPEN guideline suggests at least postoperative supplementation in malnourished individuals [9]. Optimizing nutritional and immunological status before surgery may enhance the patient’s capacity to tolerate surgical stress and modulate early inflammatory responses. In our analysis, postoperative IMNS alone did not yield comparable benefits, although the small number of available studies limits this conclusion. Overall, these findings suggest that the preoperative period may represent a critical window for influencing immune recovery and improving postoperative outcomes.

In our meta-analysis, the combination of Arg, nucleotides, and ω-3-FAs demonstrated better results than ω-3-FAs alone. Arg is essential for the biology of immune cells, contributing to anti-inflammatory immune response [6]. Nucleotides play a role in stimulating the differentiation and proliferation of lymphocytes and exhibit anti-inflammatory effects by inhibiting the secretion of TNF-α in vivo [7]. Fish oil is a common source of EPA and DHA, and has been shown to reduce proinflammatory cytokine expression, modulate inflammation levels, and decrease oxidative stress [131].

Ω-3-FAs alone did not reduce the odds of anastomotic leakage, sepsis, or urinary tract infections, highlighting the synergistic effect of the different immune-modulating nutrients [132]. This outcome contrasts with previous studies demonstrating the immunomodulatory and anti-inflammatory properties of ω-3-FAs, particularly their ability to attenuate pro-inflammatory cytokines such as IL-6 and TNF-α, which mediate the pathogenesis of sepsis [133,134]. Furthermore, recent network meta-analyses have supported the clinical utility of ω-3-FAs supplementation in the management of sepsis [135]. The observed lack of efficacy of ω-3-FAs administration in our analysis may reflect the complex metabolic environment of oncologic patients undergoing surgery. Advanced cancer and surgical stress often induce a hypercatabolic state, which may impair the bioavailability and incorporation of EPA [63], which is responsible for anti-inflammatory effects. Additionally, the efficacy of ω-3-FAs may depend on the EPA-to-DHA ratio, with evidence suggesting that ratios below 2:1, or even 1:1, are more effective in modulating inflammatory responses [136]. Another essential consideration is patient selection. Current literature advocates for ω-3-FAs supplementation primarily in patients with precachexia undergoing chemotherapy for advanced malignancies or patients with normal nutritional status [136], as both malnutrition and overweight or obesity worsen the effectiveness of ω-3 on inflammation and lean body mass [137,138]. In contrast, the populations included in our analysis were heterogeneous with respect to cancer stage and treatment type, which included both chemotherapy and surgery. Finally, variability in the route of administration (enteral compared with parenteral) and differences in dosing regimens may further explain the discordant results across studies [135,139]. Although the exact mechanism behind this phenomenon is still unclear, current evidence does not support the use of ω-3-FAs as monotherapy in preventing postoperative complications [131,140].

Our data demonstrate that perioperative IMNS containing Arg, nucleotides, and ω-3-FAs is associated with a significant reduction in postoperative complications among patients with GI cancer, particularly a decrease in anastomotic leakage and infectious complications. These results align with previous meta-analyses on upper GI cancers [14], gastric [15], and colorectal cancer [141]. Furthermore, IMNS was associated with a decreased incidence of respiratory, urinary tract, and wound infections, as well as sepsis, supporting its broader role in mitigating infection-related morbidity after oncologic surgery.

In addition to mitigating postoperative complications, IMNS was associated with a significant reduction in the LOH among patients undergoing surgical resection for GI cancer, with reductions ranging from 1 to 3 d across studies. This effect is likely attributable to improvements in postoperative recovery resulting from reduced morbidity. Even modest reductions in hospitalization duration may be clinically and economically relevant for both patients and healthcare systems [142]. However, the observed heterogeneity across studies suggests that this benefit may not be consistent across all clinical settings.

No significant reduction in mortality, either short-term (30-d) or long-term, was observed in our analysis. Although some studies [44,47,[66], [67], [68],^79^] have suggested a potential survival benefit, the limited number of available trials and wide CIs preclude definitive conclusions regarding the effect of IMNS on mortality. Importantly, these findings are consistent with the primary effects of IMNS on perioperative inflammatory and infectious pathways rather than on tumor biology, which predominantly determines survival outcomes in GI cancer. Moreover, survival is largely influenced by tumor stage, response to oncologic treatment, and the duration of follow-up in clinical studies.

Our metaregression analyses did not identify statistically significant changes in serum biomarkers associated with immune function during the follow-up period, in contrast to a previous meta-analysis in which serum white blood cell levels in GI cancer [143] and serum CRP levels in GI and colorectal cancer [141,143] significantly decreased. However, a mixed IMNS formula was analyzed in both studies. Notably, in our meta-analysis, formulations containing Arg, nucleotides, and ω-3-FAs were associated with greater reductions in inflammatory markers (CRP, IL-6) compared with ω-3-FAs alone, which also supports the synergic immunomodulatory effects of multiple immunoenhancing nutrients [132].

Patients with stage I–IV cancer demonstrated lower odds of anastomotic leakage and infectious complications following perioperative IMNS containing Arg, nucleotides, and ω-3 fatty acids compared with studies including only stage I–III disease or those in which cancer stage was not specified. These findings may suggest that the beneficial effects of IMNS are more pronounced in populations with more advanced or heterogeneous disease burden, potentially reflecting increased metabolic stress, systemic inflammation, and nutritional impairment in these patients.

Despite growing evidence supporting IMNS in GI cancer surgery, its implementation in routine clinical practice remains inconsistent. Several factors may contribute to this gap, including uncertainty about optimal formulations and timing in guideline recommendations, as well as subjective barriers such as limited nutritional support resources and patient adherence. A global survey designed by the European Society of Surgical Oncology and its young alumni club demonstrates that nutritional assessment is often neglected, making it harder to identify malnourished patients who may benefit from IMNS [144]. Our findings help address some of these uncertainties by demonstrating consistent benefits associated with perioperative administration of formulations containing Arg, nucleotides, and ω-3-FAs. These results may facilitate clearer clinical guidance and support broader integration of IMNS into perioperative oncological surgery care.

### Strengths and limitations

The strength of our analysis lies in the rigorous methodology and the inclusion of a large number of RCTs. Furthermore, we conducted several clinically relevant subgroup analyses to clarify the indications for IMNS in specific cancer types and the optimal administration time point.

There are several limitations. There was considerable clinical heterogeneity among the included studies, particularly regarding cancer site and tumor stage. These factors significantly influence postoperative outcomes, since advanced tumor stages often require a more extensive resection with a widespread systemic inflammatory reaction [145], which can affect the effectiveness of IMNS. We partially addressed subgroup analysis by cancer stage for specific outcomes, such as anastomotic leakage and infectious complications, in which IMNS showed significant effectiveness in cancer stage I–IV compared with stage I–III; however, because of the inconsistency and indirectness of the data, this should be interpreted with caution.

Additionally, malnourished patients, who are more likely to benefit from IMNS according to the ESPEN guideline [9], were overrepresented in certain studies, introducing a potential source of bias. We were unable to perform analyses that adjusted for baseline nutritional status because data were limited and inconsistently reported across studies. Many articles provided only indirect indicators, such as BMI or weight loss, without employing standardized nutritional screening methods. Moreover, the lack of uniform approaches to nutritional assessment in surgical oncology further limits objective evaluation of patients’ baseline nutritional status [144]. This heterogeneity may have influenced responsiveness to immunonutrition and thereby affected the magnitude of observed treatment effects. Furthermore, the observed superiority of combined IMNS formulations over ω-3 fatty acids alone is based on indirect comparisons between separately pooled analyses rather than direct comparison trials. Another limitation concerns the lack of standardization in control groups. Comparator interventions varied across studies, ranging from standard care without nutritional supplementation to isocaloric or isonitrogenous nutritional support, which are not clinically equivalent. Such variability may limit comparability across trials, as the incremental benefit of IMNS could be diminished in control groups that already receive nutritional support. This consideration is particularly relevant when interpreting mortality outcomes, for which only modest and statistically nonsignificant effects were observed. Moreover, variability in control group composition likely contributed to between-study heterogeneity and may have influenced the overall certainty of the evidence. This concern is reflected in the risk-of-bias assessments and GRADE evaluations, where inconsistency and indirectness led to downgrading the certainty of evidence for several outcomes.

Moreover, variability in both the duration (ranging from 3 to 10 d preoperatively, and from 5 to 37 d postoperatively), dosage, and administration route—either oral, via jejunostomy, or tube—of IMNS administered preoperatively and postoperatively may have influenced the observed outcomes (Supplemental Table 3). However, insufficient reporting across studies precluded subgroup analyses and prevented the formulation of precise recommendations regarding optimal dosing and duration.

Although we handled preoperative and postoperative analysis as individual outcomes, performing multiple calculations may still increase the type I error risk. As no correction for multiple comparisons was applied, some statistically significant findings should be interpreted with caution.

### Implications for practice and research

**FIGURE 5:**
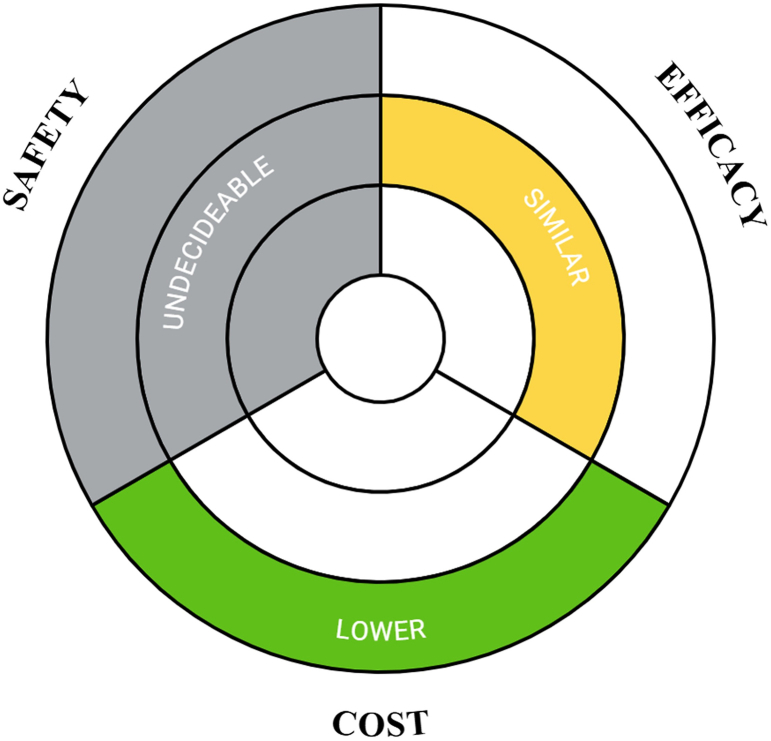
Visualization for the evaluation of translational implementation of the IMNS (Arg+nucleotides+ω-3-FA). Arg+nucleotides+ω-3-Fas, arginine + nucleotides, omega-3 fatty acids; IMNS, immunonutrition support.

The translation of scientific findings into clinical practice is essential for improving perioperative care in patients with GI cancer [20,146]. On the basis of our results, IMNS formulations containing Arg, nucleotides, and ω-3-FAs should be considered as part of preoperative management to reduce postoperative complications, including anastomotic leakage, infectious complications, and LOH. The intervention is cost-effective [48] and at least as effective as a nutritional intervention without immunonutrients, with certainty evidence ranging from very low to moderate (Figure 5) [147]. Our data do not support the use of ω-3-FAs alone to improve postoperative outcomes in patients with GI cancer undergoing surgical resection.

To optimize clinical implementation, further RCTs are warranted to establish the most effective timing, dosage, route of administration, and formula across different types of GI cancers. Additionally, well-designed prospective observational studies are needed to assess the effectiveness of IMNS across patients with varying nutritional status, using standardized nutritional screening tools and predefined nutritional phenotypes, including disease-related malnutrition and cachexia. Preoperative delivery should be considered.

In conclusion, IMNS containing Arg, nucleotides, and ω-3-FAs is associated with significant improvements in postoperative outcomes in patients with GI cancer. It substantially reduces the risk of anastomotic leakage and infectious complications and decreases the LOH. However, it does not appear to have a significant impact on short- or long-term mortality. Nevertheless, the level of evidence in this regard is low moderate, mainly due to the heterogeneity in the administered formula and baseline characteristics of the control arms across the included studies.

## Author contributions

The authors’ responsibilities were as follows – BCB: contributed to conceptualization, project administration, methodology, formal analysis, and writing – original draft; RP: contributed to methodology and writing – review and editing; BL: methodology, writing – review and editing; GC: contributed to methodology, writing – review and editing, SV: contributed to methodology, writing – review and editing; DSV: contributed to data curation, writing – review and editing; KKF: contributed to data curation, writing – review and editing; EÁS: contributed to conceptualization, methodology, writing – review and editing; KH: contributed to writing – review and editing; BE: contributed to writing – review and editing; LF-N: contributed to writing – review and editing; SB: contributed to conceptualization, supervision, writing – original draft; PH: contributed to conceptualization, supervision, writing – review and editing; and all authors: certify that they have participated sufficiently in the work to take public responsibility for the content, including participation in the manuscript's concept, design, analysis, writing, or revision.

## Data availability

The datasets used in this study can be found in the full-text articles included in the systematic review and meta-analysis.

## Ethical approval

No ethical approval was required for this systematic review with meta-analysis, as all data had already been published in peer-reviewed journals. No patients were involved in our study's design, conduct, or interpretation.

## Funding

The research was supported by the Hungarian Ministry of Innovation and Technology, National Research, Development and Innovation Fund (TKP2021-EGA-23 to PH), Translational Neuroscience National Laboratory program (RRF-2.3.1-21-2022-00011 to PH), project grants (K131996 and K147265 to PH), a Hirschberg Foundation seed grant (HF-2023-084), and the Translational Medicine Foundation. The funders did not affect the manuscript's concept, data collection, analysis, or writing.

## Declaration of generative AI and AI-assisted technologies in the writing process

The authors declare that no generative AI or AI-assisted technologies were used in the writing of this manuscript.

## Conflict of interest

The authors report no conflicts of interest.
