# Supplementary material for: Immunonutrition Decreases Postoperative Complications in Gastrointestinal Cancer—A Systematic Review and Meta-analysis of Randomized Controlled Trials
Source: Adv Nutr. 2026 Jun 22;17(8):100690. doi: 10.1016/j.advnut.2026.100690 (PMC13384005; doi:10.1016/j.advnut.2026.100690)
Supplement: multimedia component 1 [file mmc1.pdf]

Bettina Csilla Budai
Immunonutrition Decreases Postoperative Complications in Gastrointestinal Cancer – A Systematic Review and Meta-analysis of Randomized Controlled Trials
Supplementary Table 2: Basic characteristics Table

First Author	Year of publication	Study design	Country	Centres	Study period	Total number of patients	Age in the immunonutrition group	Age in the control group	Gender (number)	Cancer type	Prevalence of malnutrition (n)	Type of Immunonutrition	definition/nutrients
M. Aba	2024	RCT	Turkey	1	2020-2021	40	¶60,95± 11,13	¶62,05± 9,99	14	periampullary cancer	0 pts. (0%)	NA	arginine, omege-3 fatty acids and RNA (dietary nucleotides)
A. Adiamah	2021	RCT	UK	2	January 2000- June 2003	108	¶65,7± 1,4	¶66,6± 1	25	esophagogastric; pancreatobiliary	BMI<19 kg/m^2: 9 pts (8,3%)	Stresson	arginine, glutamine, omega-3 fatty acid
R. Ashida	2019	RCT	Japan	1	September 2012-September 2013	20	¶64± 11	¶69± 6	9	periampullary cancer	weight loss >10%: 5 pts (25%)	Prosure	EPA-enriched enteral formula
E. Ateş	2004	RCT	Turkey	NA	June 1998 - December 2001	42	¶58,3± 6,2	¶60,1± 4,2	8	gastric; colorectal	NA	Impact	arginine, omege-3 fatty acids and RNA (dietary nucleotides)
J. A. Benavides-Buleje	2022	RCT	Spain	multicenter	May 2019 - January 2021	143	¶69,6± 11,8	¶69,8± 11,7	59	colorectal	0 pts. (0%)	Impact or Atempero	arginine, omege-3 fatty acids and RNA (dietary nucleotides)

F. Bozzeti	2007	RCT	Italy	database of different RCT's	NA	863	NA	NA	834	gastrointestinal cancer (colorectal, gastric, pancreas)	weight loss >10%: 806 pts. (93%)	Impact	arginine, omege-3 fatty acids and RNA (dietary nucleotides)
M. Braga	1999	RCT	Italy	1	NA	206	¶60,9± 11,9	¶60,8± 9,7	65	colorectum; gastric; pancreas	40 pts. (19,4%)	Impact	arginine, omege-3 fatty acids and RNA (dietary nucleotides)
M. Braga	2002	RCT	Italy	1	NA	200	¶60,5± 11,5	¶61,8± 9,9	41	colorectal	weight loss >10%: 20 pts (10%)	Impact	arginine, omege-3 fatty acids and RNA (dietary nucleotides)
M. Braga	2005	RCT	Italy	1	NA	305	NA	NA	NA	gastrointestinal	NA	Impact	arginine, omege-3 fatty acids and RNA (dietary nucleotides)
M. Braga	1996	RCT	Italy	1	NA	40	¶64,1± 12,5	¶58,1± 9,3	15	gastric; colorectal	weight loss >10%: 6 pts. (15%)	Impact	arginine, omege-3 fatty acids and RNA (dietary nucleotides)
M. Braga	1998	RCT	Italy	1	NA	30	¶65,2± 12,5	¶63,4± 11,2	11	gastric	weight loss >10%: 11 pts. (36%)	Impact	arginine, omege-3 fatty acids and RNA (dietary nucleotides)

D. W. Chen	2005	RCT	China	1	September 2002- August 2003	40	NA	NA	12	gastric	NA	Stresson	arginine, glutamine, omega-3 fatty acid
O. Ciacio	2021	RCT	France	6	September 2013- June 2018	399	NA	NA	NA	hepatocellular carcinoma	NA	Impact	arginine, omege-3 fatty acids and RNA (dietary nucleotides)
N. Farreras	2005	RCT	Spain	2	1st January 1999 - 30th September 2000	60	¶66,7± 8,3	¶69,2± 13,8	28	gastric	weight loss >10%: 13 pts. (21,6%)	Impact	arginine, omege-3 fatty acids and RNA (dietary nucleotides)
K. Fujitani	2012	RCT	Japan	NA	16th February 2006 - 25th December 2009	244	$64 &26-78	$65 &30-79	63	gastric	5 pts. (2%)	Impact	arginine, omege-3 fatty acids and RNA (dietary nucleotides)
A. Gencer	2010	RCT	Turkey	1	NA	60	¶65± 12,2	¶67,3± 11,6	24	gastric; colorectal	weight loss >10%: 36 pts. (60%)	Impact	arginine, omege-3 fatty acids and RNA (dietary nucleotides)
L. Gianotti	1999	RCT	Italy	1	NA	50	¶62,5± 11,3	¶60,9± 12,5	20	gastric; colorectal	weight loss >10%: 11 pts. (22%)	Impact	arginine, omege-3 fatty acids and RNA (dietary nucleotides)

L. Gianotti	1997	RCT	Italy	1	1st January 1993- 18th March 1997	260	¶62,7± 14,3	¶64,5± 13,4	75	gastric; pancreas	NA	Impact	arginine, omege-3 fatty acids and RNA (dietary nucleotides)
U. Giger	2007	RCT	Switzerland	1	January 2001 - December 2001	29	¶64,4 &30-84	¶63 &47-79	12	gastric; pancreas	18 pts. (62%)	Impact	arginine, omege-3 fatty acids and RNA (dietary nucleotides)
U. Giger-Pabst	2013	RCT	Switzerland	multicenter	January 2006 - May 2008	108	¶64,9± 13,6	¶63,2± 11,8	42	upper or lower GI tract adenocarcinoma (esophagus, gastric, pancreas, liver, colon, rectum)	0 pts. (0%)	Impact	arginine, omege-3 fatty acids and RNA (dietary nucleotides)
M. B. Gómez Sánchez	2010	RCT	Spain	1	July 2006- March 2009	82	¶77± 7,02	¶74,23± 11,16	48	colorectal	26 pts. (31,7%)	Impact	arginine, omege-3 fatty acids and RNA (dietary nucleotides)
M. O. Gul	2022	RCT	Turkey	2	March 2018- December 2019	30	¶62,4± 2,47	¶57,9± 1,82	13	rectal	5 pts. (60%)		arginine, omega-3 fatty acids, nucleotids
Y. Gunerhan	2009	RCT	Turkey	1	NA	42	¶64,56± 16,16	¶61,31± 12,13	16	gastrointestinal	42 pts. (100%)	Impact	arginine, omege-3 fatty acids and RNA

													(dietary nucleotides)
L. Healy	2017	RCT	Ireland	1	January 2011-December 2014	191	$62 &31-77	$62 &38-82	40	esophageal	75 pts. (39,2%)	Prosure	EPA enriched enteral formula
S. Kanekiyo	2019	RCT	Japan	1	NA	40	$65 ^60-70	$62 ^60-72	8	esophageal	10 pts. (25%)	Impact	arginine, omege-3 fatty acids and RNA (dietary nucleotides)
Y. Kimura	2011	RCT	Japan	12	February 2004-December 2009	240	$63,5 &29-78	$66 &30-79	62	gastric	5 pts. (2%)	Impact	arginine, omege-3 fatty acids and RNA (dietary nucleotides)
S. Klek	2017	RCT	Poland	1	2003-2009	99	¶62,8± 11,9; $65 ^54-70; &33-86	¶63± 10,7, $64 ^54-70; &35-82	27	gastric	99 pts. (100%)	Reconvan	arginine, glutamine
S. Klek	2014	RCT	Poland	1	January 2001-December 2009	776	NA	NA	407	gastric; pancreas	776 pts. (100%)	Reconvan	arginine, glutamine
S. Lewis	2018	RCT	USA	1	November 2011-January 2016	108	¶64,5± 8,7	¶63,4± 10,1	7	esophagus, gastric, pancreas, colorectal, liver	79 pts. (73,1%)	NA	omega-3 fatty acids, arginine, nucleotides

C. Li	2020	RCT	China	1	August 2014- April 2016	78	¶61,4± 5,5	¶61,2± 5,9	35	pancreas	NA	NA	arginine, ω-3 polyunsaturated fatty acids and dietary fiber
K. Li	2020	RCT	China	1	June 2017- August 2018	118	¶57,32± 10,19	¶55,02± 9,61	54	gastric	0 pts. (0%)	NA	arginine, glutamine, omega-3 fatty acids, nucleotides, has an omega-3 to omega-6 ratio of 0.4
X. K. Li	2021	RCT	China	1	1st December 2017- 1st March 2018	103	¶62,13± 6,51	¶61,52± 5,97	32	esophageal	NA	NA	arginine, RNA, omega-3 fatty acids
D. N. Lobo	2006	RCT	UK	3	January 2000- June 2003	108	¶65,7± 1,4	¶66,6± 1,4	25	esophagus; gastric; pancreas	BMI<19 kg/m^2: 9 pts (8,3%)	Stresson	arginine, glutamine, omega-3 fatty acid
M. Ma	2023	RCT	China	1	June 2022- June 2023	65	¶61,52± 9,27	¶62,51± 9,63	21	gastric	NA	Prosure	EPA-enriched enteral formula
M. del Carmen Manzanares	2017	RCT	Spain	1	1st December 2010- 31st October 2011	84	¶67,7± 11,2	¶72± 10,5	26	colorectal	54 pts. (64,2%)	Impact	arginine, omege-3 fatty acids and RNA (dietary nucleotides)
L. Marano	2013	RCT	Italy	1	2006-2011	109	¶66,6 &55-78	¶65,1 &49-83	38	gastric	63 pts. (57,7%)	Impact	arginine, omege-3 fatty acids and RNA

													(dietary nucleotides)
R.C.G. Martin	2017	RCT	USA	1	January 2012- December 2013	71	$60 ^27-81	$62 ^47-75	39	pancreas	47 pts. (66,1%)	Impact	arginine, omege-3 fatty acids and RNA (dietary nucleotides)
Y. Matsuda	2017	RCT	Japan	1	November 2009- July 2011	72	¶64,1± 8,3	¶64,6± 6,4	18	esophageal	weight loss: 25 pts. (34,7%)	Oxepa	arginine, omege-3 fatty acids and RNA (dietary nucleotides)
A. Matsuda	2006	RCT	Japan	1	NA	36	¶65,9± 2	¶63,2± 3,1	17	colorectal	0 pts. (0%)	Impact	arginine, omege-3 fatty acids and RNA (dietary nucleotides)
M. D. McCarter	1998	RCT	USA	1	NA	38	¶62± 2,3	¶66± 4,4	17	esophagus; gastric; pancreas	8 pts. (21%)	NA	standard nutritional supplement added arginine and omega-3 fatty acid
K. Mikagi	2011	RCT	Japan	1	February 2005- December 2008	26	¶67,5± 11,3	¶61,5± 10,2	8	hepatocellular carcinoma, cholangiocellular carcinoma, metastatic	NA	Impact	arginine, omege-3 fatty acids and RNA (dietary nucleotides)

										liver carcinoma and carcinoid			
P. Moya	2016	RCT	Spain	6	January 2014- March 2015	244	$70 &42-88	$68 &41-89	113	colorectal	0 pts. (0%)	Atempero	arginine, omege-3 fatty acids and RNA (dietary nucleotides)
T. Moriya	2014	RCT	Japan	1	NA	88	NA	NA	NA	colorectal	NA	Impact	arginine, omege-3 fatty acids and RNA (dietary nucleotides)
L. A. Mudge	2018	RCT	Australia	11	November 2009- October 2014	276	¶64,6± 8,6	¶64,6± 8,2	53	esophageal	41 pts. (14,8%)	Impact	arginine, omege-3 fatty acids and RNA (dietary nucleotides)
Y. Okamoto	2009	RCT	Japan	1	April 2005- July 2007	60	¶66,9± 11,5	¶70,9± 13,2	18	gastric	NA	Impact	arginine, omege-3 fatty acids and RNA (dietary nucleotides)
Y. Sakurai	2007	RCT	Japan	1	NA	30	¶63± 4	¶63± 5	17	esophageal	NA	Impact	arginine, omege-3 fatty acids and RNA (dietary nucleotides)

M. Senkal	1999	RCT	Germany	2	1st April 1994- 30st August 1997	178	¶64± 11	¶67± 9	54	upper GI tract	NA	Impact	arginine, omege-3 fatty acids and RNA (dietary nucleotides)
M. Senkal	1997	RCT	Germany	2	April 1992- May 1994	154	¶65,1± 1,5	¶66,3± 1,8	NA	upper GI tract	NA	Impact	arginine, omege-3 fatty acids and RNA (dietary nucleotides)
R. Slotwinski	2008	RCT	Poland	NA	NA	41	¶59,8± 6	¶54,2± 4,1	12	pancreas	41 pts. (100%)	Stresson	arginine, glutamine, omega-3 fatty acid
L. S Sorensen	2014	RCT	Denmark	1	July 2007- January 2010	148	¶69± 11	¶71± 10	68	colorectal	weight loss >5%: 30 pts. (20,2%)	Supporta n	enriched with omega-3 fatty acids (EPA: 2g/day; DHA: 1 g/day)
J. Sultan	2012	RCT	United Kingdom	NA	April 2003- January 2007	195	$67 &42-79	$60 &42-79	34	esophagea l or gastric	16 pts. (8,2%)	Oxepa	arginine, omege-3 fatty acids and RNA (dietary nucleotides)
D. Suzuki	2010	RCT	Japan	1	May 2006- January 2008	30	¶62± 4	¶66± 3	12	pancreas, bile duct carcinoma , ampullary carcinoma , other	NA	Impact	arginine, omege-3 fatty acids and RNA (dietary nucleotides)

G. H. Wu	2001	RCT	China	1	NA	48	¶55,2± 12,1	¶52,6± 9,8	17	gastrointestinal cancer (gastric, colorectal, pancreas)	NA	Stresson	arginine, glutamine, omega-3 fatty acid
J. Xu	2006	RCT	China	1	January 2003-December 2003	60	¶60,05± 10,15	¶57,68± 11,50	24	colorectal; gastric	NA	Impact	arginine, omege-3 fatty acids and RNA (dietary nucleotides)

¶= *mean; $= median; &= range; ^= interquartile range; NA= not available; pts.= patients*

Supplementary Table 3: Basic characteristics Table for detailed intervention

**First Author**	**Year of publication**	**Type of Immunonutrition**	**definition/nutrients**	**Route of administration**	**Doses of the intervention**	**Duration if there is no surgery**	**Duration of administration - preoperative**	**Duration of administration - postoperative**	**Control**	**Extra diet**
M. Aba	2024	NA	arginine, omege-3 fatty acids and RNA (dietary nucleotides)	oral	3 times/day (237 mL/ piece)	NA	5 days	NA	standard enteral nutrition	NA
A. Adiamah	2021	Stresson	arginine, glutamine, omega-3 fatty acid	jejunostomy	25 mL/h day 0, 50mL/h day 1 and 75mL/h thereafter (delivering 20h/day with a 4h rest period)	NA	NA	10-15 days	isonitrogenous, isocaloric control feed (Nutrison High Protein e Nutricia Ltd)	NA
R. Ashida	2019	Prosure	EPA-enriched enteral formula	oral	600 kcal/day and EPA 2.0 g/day	NA	7 days	NA	isocaloric standard nutrition	1200 kcal regular food
E. Ateş	2004	Impact	arginine, omege-3 fatty acids and RNA (dietary nucleotides)	before surgery oral, after surgery nasoduodenal catheter	started as a rate of 0.2ml/kg/hr at postoperative period and increased progressively until the nutritional goal (2ml/kg/hr) was reached at POD4. Enteral nutrition was integrated with parenteral nutrition to reach the	NA	5 days	7 days	isonitrogenous and isocaloric TPN (preoperative 5 days, postoperative 7 days)	NA

					nutritional energy goal until POD4 in all patients of EEN group.					
J. A. Benavides -Buleje	2022	Impact or Atempero	arginine, omege-3 fatty acids and RNA (dietary nucleotides)	oral	200 ml	NA	NA	5 days	200 mL oligomeric hyperproteic normocaloric supplement	NA
F. Bozzeti	2007	Impact	arginine, omege-3 fatty acids and RNA (dietary nucleotides)	jejunostomy or nasojejunal tube	progressively increased by 20 mL h^{-1} day-1 until reaching the full nutritional goal (25–28 kcal kg-1 day-1).	NA	NA	continued until patients resumed adequate oral food intake (about 50% of basal energy requirement)	1. group: enteral nutrition; 2. group: total parenteral nutrition	
M. Braga	1999	Impact	arginine, omege-3 fatty acids and RNA (dietary nucleotides)	jejunostomy	infusion rate of 10 ml/h, which was progressively increased up to a volume of 1500 ml/d on POD3	NA	NA	started 6 hours after surgery until postoperativ e day 7	isonitrogenous, isoenergeric liquid diet	standard food form POD7
M. Braga	2002	Impact	arginine, omege-3 fatty acids and RNA (dietary nucleotides)	preoperative oral; postoperative enteral (jejunostomy or nasojejunal feeding)	1 liter/day	NA	5 day	administrati on was prolonged in the postoperativ e course by jejunal infusion	1. group: preoperative immunonutrition for 5 days before operation; 2. group: 1 L/day of an isonitrogenous,	regular food ad libitum during the preoperative 5 days

								through a naso-enteric tube.	isoenergetic, specially formulated liquid diet for 5 days before surgery; 3. group: not received any diet	
M. Braga	2005	Impact	arginine, omege-3 fatty acids and RNA (dietary nucleotides)	oral	NA	NA	5 days	NA	1. group: postoperative immunonutrition (jejunostomy) for postop. 7 days; 2. group: no supplementation	NA
M. Braga	1996	Impact	arginine, omege-3 fatty acids and RNA (dietary nucleotides)	preoperatively oral; postoperatively: enteral (nasojejunal tube)	preoperatively: 1000ml/ day; postoperatively: 6 hours after surgery with 10 ml/h progressively increased to 25 kcal/ttkg	NA	7 days	8 days	preoperatively: control liquid, postoperatively: control enteral formula	standard hospital diet (avarage 1200 kcal/day, 100 g protein/day)
M. Braga	1998	Impact	arginine, omege-3 fatty acids and RNA (dietary nucleotides)	preoperatively oral; postoperatively: enteral (nasojejunal or nasogastric tube)	1 liter/day	NA	7 days	starting 6 hours after surgery for 7 days	only postoperative immunonutrition (1000 ml/day)	standard food (1200 kcal/day and 100 g protein/day)
D. W. Chen	2005	Stresson	arginine, glutamine, omega-3 fatty acid	nasoenteric tube	On postoperative Day 2, patients received 25% of the planned caloric goal, based on 30 non-protein calories/kg/day.	NA	NA	9 days	standard enteral nutrition (Nutrison)	NA

					On postoperative Day 3, 50% of the planned caloric goal was given. From postoperative Day 4 until the end of the study, patients received 100% of the planned caloric goal.					
O. Ciacio	2021	Impact	arginine, omege-3 fatty acids and RNA (dietary nucleotides)	oral	NA	NA	7 days	NA	isocaloric supplements	NA
N. Farreras	2005	Impact	arginine, omege-3 fatty acids and RNA (dietary nucleotides)	jejunostomy	POD1:20 ml/h, POD2: 31 ml/h, POD3: 50 ml/h from POD4: adjusted to caloric requirements	NA	NA	started 12-18 h after surgery, 7 days	control formula (Isosource Protein)	NA
K. Fujitani	2012	Impact	arginine, omege-3 fatty acids and RNA (dietary nucleotides)	oral or tube feeding	1000 ml/day	NA	5 days	NA	regular diet without any nutritional supplementation	normal diet
A. Gencer	2010	Impact	arginine, omege-3 fatty acids and RNA (dietary nucleotides)	preoperatively: oral; postoperatively: nasogastric tube or nasojejunal tube or jejunostomy	preoperatively: half of the energy requirement; preoperatively: increased with 20 ml/h to	NA	5 days	5 days	half of the reqirement norma diet, other half with TPN (75% charbohydrate, 25% fat)	half of the energy normal diet

					reach 35 kcal/kg/day					
L. Gianotti	1999	Impact	arginine, omege-3 fatty acids and RNA (dietary nucleotides)	preoperatively oral; postoperatively: enteral (nasojejunal tube)	preoperatively 1 liter/day; postoperatively increased progressively by 20 mL/h per day until the full nutritional regimen (25 kcal/kg per day)	NA	7 days	6 hours after surgery for 7 days	standard enteral diet preoperatively oral, postoperatively enteral tube feeding	preoperatively standard meal
L. Gianotti	1997	Impact	arginine, omege-3 fatty acids and RNA (dietary nucleotides)	pancreaticoduodenectomy: enteral (jejunostomy), gastrectomy: enteral (nasojejunal tube)	started 6 hours after surgery with 10 ml/h, increased by 20 ml/h until reaching full nutritional goal (105 kJ/kg/day)	NA	NA	7 days	1. group: standard enteral formula; 2. group: total parenteral nutrition	NA
U. Giger	2007	Impact	arginine, omege-3 fatty acids and RNA (dietary nucleotides)	oral or jejunostomy	1 liter/day	NA	5 days	7 days; 20 mL/hour and it was progressively increased up to 60 or 80 mL/hour at postoperative day 3. The diet was intended to provide 25 kcal/kg/day.	control: no preoperative immunonutrition, but postoperative immunonutrition (Impact)	NA
U. Giger-Pabst	2013	Impact	arginine, omege-3 fatty acids and RNA	oral	750 ml/day	NA	3 days	NA	isocaloric and isonitrogenous placebo	

			(dietary nucleotides)							
M. B. Gómez Sánchez	2010	Impact	arginine, omege-3 fatty acids and RNA (dietary nucleotides)	oal	2x237 ml (2x1 pack)	NA	10 days	NA	1. group: rest of malnourished-intravenous fluids until the reintroduction of normal diet; 2. group: non-malnourished patients-intravenous fluids until the reintroduction of normal diet	NA
M. O. Gul	2022		arginine, omega-3 fatty acids, nucleotids	oral	3x daily	NA	7 days	NA	standard nutritional supplement group	standard diet
Y. Gunerhan	2009	Impact	arginine, omege-3 fatty acids and RNA (dietary nucleotides)	oral	1 liter/day	NA	7 days	NA	1. group: normal nutriiton (normal feeding planned by a dietitian); 2. group: standard enteral nutrition (Fresubin)	NA
L. Healy	2017	Prosure	EPA enriched enteral formula	preoperatively oral; postoperatively: enteral (jejunostomy)	2.2g EPA/day	NA	5 days	1 month	isocaloric, isonitrogenous standard nutritional feed (Ensure Plus)	oral diet established progressively in the postoperative phase
S. Kanekiyo	2019	Impact	arginine, omege-3 fatty acids and RNA (dietary nucleotides)	preoperatively oral; postoperatively: enteral (jejunostomy)	750 kcal/day	NA	7 days	7 days; started 6 h after surgery	standard enteral nutrition (Ensure)	regular meals

Y. Kimura	2011	Impact	arginine, omege-3 fatty acids and RNA (dietary nucleotides)	oral	1000 ml/day	NA	5 days	NA	free oral ingestion of regular diet	regular diet
S. Klek	2017	Reconvan	arginine, glutamine	enteral tube	20 mL/h on day 1, 50 mL/h on day 2, 75 mL/h on day 3, and 100 mL/h	NA	NA	7 days	standard enteral nutrition (Peptisorb)	NA
S. Klek	2014	Reconvan	arginine, glutamine	nasojejunal tube	20 ml/h on day 1, 50 ml/h on day 2, 75 ml/h on day 3, and 100 ml/h	NA	NA	7 days	1. group: standard parenteral nutrition; 2. group: immunomodulating parenteral nutrition; 3. group: standard enteral nutrition (Peptisorb)	all malnourished patients received intravenous nutrition the fortnight before surgery
S. Lewis	2018	NA	omega-3 fatty acids, arginine, nucleotides	oral	750 mL/day (3 servings)	NA	5 days	NA	standard nutrition group	normal diet
C. Li	2020	NA	arginine, ω-3 polyunsaturated fatty acids and dietary fiber	nasoduodenal tube	1st day one third of total dosage, then it was increased at 30 ml/h to 80 ml/h	1 course of chemotherapy treatment	NA	NA	conventional nutrition support	
K. Li	2020	NA	arginine, glutamine, omega-3 fatty acids, nucleotides, has an omega-3 to omega-6 ratio of 0.4	enteral tube	16 ml/h for the first hour; 40 ml/h for day 2; 56 ml/h for day 3; maximm 80 ml/h for day 4 and 5	NA	NA	5 days	standard enteral nutrition (with an omega-3 to omega-6 ratio of 0.2)	NA

X. K. Li	2021	NA	arginine, RNA, omega-3 fatty acids	preoperatively: oral; postopertively: jejunostomy	preoperatively: 500 ml/day (750 kcal/day); postopertively: started with 20-25 ml/h, gradually increased to 70-90 ml/h; from discharge 500 ml/day (750 kcal/day)	NA	7 days	37 days	enteral nutrition (Peptisorb)	oral diet from postopertive day 7
D. N. Lobo	2006	Stresson	arginine, glutamine, omega-3 fatty acid	jejunostomy	25 ml/h on day 0 (day of operation), 50 ml/h on day 1 and 75 ml/h thereafter	NA	NA	10-15 days	isonitrogenous, isocaloric control feed (Nutrison High Protein–Nutricia)	NA
M. Ma	2023	Prosure	EPA enriched enteral formula	oral	amount that was equivalent to 20 kcal/kg/day	NA	4 days	from 3 to 14 days	enteral nutrition equivalent to 20 kcal/kg/day (Ensure group-1.5 kcal/mL and 6.25 g of protein per 100 mL)	regular meals
M. del Carmen Manzanares	2017	Impact	arginine, omege-3 fatty acids and RNA (dietary nucleotides)	oral	3x237 ml bottles per day	NA	8 days	NA	rutin normal preoperative managment with normal diet	normal diet
L. Marano	2013	Impact	arginine, omege-3 fatty acids and RNA (dietary nucleotides)	jejunostomy	begin with 10 ml/h, and increased in every 12 h with 10 ml/h until maximum 80 ml/h - 35 kcal/kg/day	NA	NA	7 days	isonitrogenic and isoenergetic enteral standard nutrition (Jevity 1 Cal)	NA

R.C.G. Martin	2017	Impact	arginine, omege-3 fatty acids and RNA (dietary nucleotides)	oral	3 pack/day	NA	5 days	NA	no supplemental treatment	NA
Y. Matsuda	2017	Oxepa	arginine, omege-3 fatty acids and RNA (dietary nucleotides)	jejunostomy	started with 10 ml/h, increased by 10 ml/h every 2 days to 30 ml/h	NA	NA	21 days	standard isocaloric, isonitrogenous diet (Pulmocare)	introduced on POD7, enteral feeding was reduced to 400 ml/day
A. Matsuda	2006	Impact	arginine, omege-3 fatty acids and RNA (dietary nucleotides)	oral	750 ml/day	NA	5 days	NA	control - did not receive artificial nutritional supplementation	standard diet
M. D. McCarter	1998	NA	standard nutritional supplement added arginine and omega-3 fatty acid	oral	750 ml/day	NA	7 days	NA	1. group: standard nutritional supplement; 2. group: standard nutritional supplement added arginine	normal meals
K. Mikagi	2011	Impact	arginine, omege-3 fatty acids and RNA (dietary nucleotides)	oral	750 ml/day	NA	5 days before the days before surgery	NA	conventional hospital meals (1800 kcal/day)	half-size hospital meal (1000 kcal/day)
P. Moya	2016	Atempero	arginine, omege-3 fatty acids and RNA (dietary nucleotides)	oral	2x400 ml/day preop and postoperatively as well	NA	7 days	5 days	hypercaloric, high-protein supplement (HHS)-SUPRESSI of Vegenat)	normal food intake
T. Moriya	2014	Impact	arginine, omege-3 fatty acids and RNA	oral	750 ml/day	NA	5 days	NA	NA	NA

			(dietary nucleotides)							
L. A. Mudge	2018	Impact	arginine, omege-3 fatty acids and RNA (dietary nucleotides)	oral; in case of dyphagia: nasogastric tube or a jejunostomy	3x74g sachets/day (prepare with 250 ml water - 900ml)	NA	7 days	NA	1. group: perioperative isocaloric and isonitrogenous standard nutrition; 2. group: preoperative isocaloric and isonitrogenous standard nutrition and postoperative immunonutrition ; 3. group: perioperative immunonutrition	usual diet
Y. Okamoto	2009	Impact	arginine, omege-3 fatty acids and RNA (dietary nucleotides)	oral	750ml/day	NA	7 days	NA	isoenergetic standard formula (Medif)	standard meals
Y. Sakurai	2007	Impact	arginine, omege-3 fatty acids and RNA (dietary nucleotides)	preoperatively: oral; postoperatively: jejunostomy	preoperatively: 1000 kcal/day; postoperatively: started from 250 kcal/day, and the calories were progressively increased daily in a stepwise manner	NA	3 days	14 days	regular polymeric enteral formula (Ensure)	regular diet
M. Senkal	1999	Impact	arginine, omege-3 fatty acids and RNA	preoperatively: oral; postoperatively: jejunostomy	preoperatively: 1000ml/day (250ml	NA	5 days	5 days	isoenergetic control group	preoperatively: usual hospital diet

			(dietary nucleotides)		portions); postoperatively: started at 20 ml/h, and progressed to the optimal goal (80 ml/h) by the 5th postoperative day					
M. Senkal	1997	Impact	arginine, omege-3 fatty acids and RNA (dietary nucleotides)	jejunostomy	started at 20 ml/h, and progressed to the optimal goal (80 ml/h) by the 5th postoperative day	NA	NA	5 days	isocaloric and isonitrogenous placebo diet	NA
R. Slotwinski	2008	Stresson	arginine, glutamine, omega-3 fatty acid	tube feeding	started from 30 ml/h, then increased to full feeding within 72 hours	NA	NA	12.3±2.0 days	standard diet (Nutrison)	NA
L. S Sorensen	2014	Supportan	enriched with omega-3 fatty acids (EPA: 2g/day; DHA: 1 g/day)	oral	2x200ml	NA	7 days	7 days	standard oral nutrition supplement (isocaloric and isonitrogenous)	
J. Sultan	2012	Oxepa	arginine, omege-3 fatty acids and RNA (dietary nucleotides)	oral; in malnourished patients: jejunostomy	preoperatively: 675 ml/day; postoperatively: feeding was commenced at 25 ml/h, increased by the third day to 50 ml/h, reaching the	NA	7 days	7 days	1. group: standard enteral nutrition (Ensure Plus); 2. group: control with lower energy and protein content (Osmolite)	normal diet

					desired maximum rate					
D. Suzuki	2010	Impact	arginine, omege-3 fatty acids and RNA (dietary nucleotides)	oral	1000 kcal/day	NA	5 days	NA	1. group: postoperative immunonutrition ; 2. group: control (preoperative total parenteral nutrition)	half amount of ordinary diet for the preoperative treatment group
G. H. Wu	2001	Stresson	arginine, glutamine, omega-3 fatty acid	jejunostomy, or nasoenteric tube	started 50 ml/h, and reached the nutrition goal by 72 ml/h; goal was: 146kJ/kg/d and 2,2g protein/kg/day	NA	7 days	NA	isocaloric standrad diet (Nutrison + protein powder- to make isonitrogenous)	NA
J. Xu	2006	Impact	arginine, omege-3 fatty acids and RNA (dietary nucleotides)	nasal feeding catheter	25kcal/kg/day	NA	7 days	NA	isocaloric and isonitrogenous conventional diet	standard food as desired before surgery

NA= not applicable

Supplementary Table 4: Risk of bias assessment

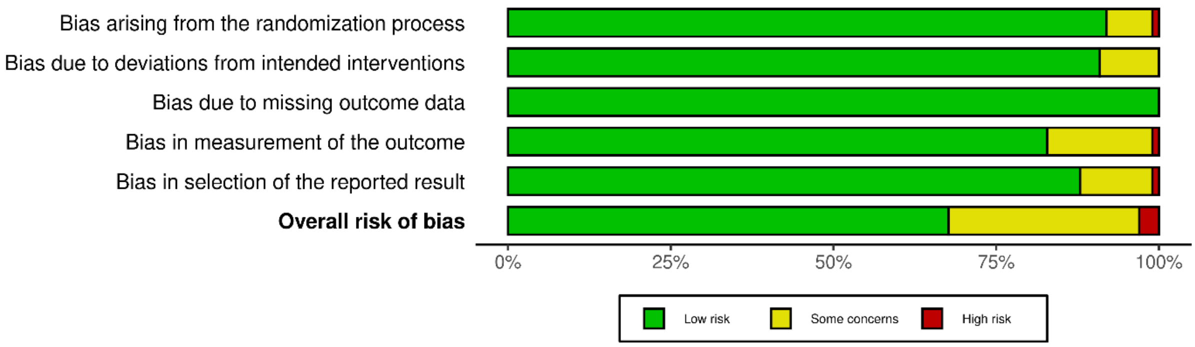

Study	D1	D2	D3	D4	D5	Overall
Aba, 2024	Low	Low	Low	Low	Low	Low
Adiamah, 2021a	Low	Low	Low	Low	Low	Low
Adiamah, 2021b	Low	Low	Low	Low	Some concerns	Some concerns
Angka, 2022	Low	Low	Low	Low	Low	Low
Ashida, 2019	Low	Low	Low	Low	Low	Low
Ateş, 2004	Low	Low	Low	Low	Low	Low
Aybar, 2022	Some concerns	Some concerns	Low	Low	Some concerns	Some concerns
Benavides-Buleje, 2022	Low	Low	Low	Low	Low	Low
Bozzetti, 2007	Some concerns	Low	Low	Low	Low	Some concerns
Braga, 1995	Low	Low	Low	Low	Low	Low
Braga, 1996	Low	Low	Low	Low	Low	Low
Braga, 1998a	Low	Some concerns	Low	Low	Low	Some concerns
Braga, 1998b	Low	Low	Low	Low	Low	Low
Braga, 2002a	Low	Low	Low	Low	Low	Low
Braga, 2002b	Low	Low	Low	Low	Low	Low
Braga, 2005	Low	Low	Low	Low	Some concerns	Some concerns
Braga, 1999	Low	Low	Low	Low	Low	Low
Chen, 2005a	Low	Low	Low	Low	Low	Low
Chen, 2005b	Low	Low	Low	Low	Low	Low
Ciacio, 2021	Low	Low	Low	Low	Some concerns	Some concerns
Ciacio, 2022	Low	Low	Low	Low	Some concerns	Some concerns
Faber, 2015	Some concerns	Low	Low	Low	Low	Some concerns

Farreras, 2005	Low	Low	Low	Low	Low	Low
Fujitani ,2012	Low	Low	Low	Low	Low	Low
Gencer, 2010	Low	Low	Low	Low	Low	Low
Gianotti, 1997	Low	Low	Low	High	Some concerns	High
Gianotti, 1999	Low	Low	Low	Low	Low	Low
Giger, 2007	Low	Low	Low	Some concerns	Low	Some concerns
Giger-Pabst, 2013	Low	Low	Low	Low	Low	Low
Gómez Sánchez, 2010	Low	Low	Low	Some concerns	Low	Some concerns
Gul, 2022	Low	Low	Low	Low	Low	Low
Gunerhan, 2009	Low	Low	Low	Some concerns	Low	Some concerns
Healy, 2017	Low	Low	Low	Low	Low	Low
Heller, 2004	Low	Low	Low	Low	Low	Low
Heslin, 1997	Low	Low	Low	Some concerns	Low	Some concerns
Hu, 2012	Low	Some concerns	Low	Low	High	High
Jiang, 2010	Low	Low	Low	Low	Low	Low
Kamocki, 2023	Low	Low	Low	Low	Low	Low
Kanekiyo, 2018	Low	Some concerns	Low	Low	Some concerns	Some concerns
Kanekiyo, 2019	Low	Low	Low	Low	Low	Low
Kimura, 2011	Low	Low	Low	Low	Some concerns	Some concerns
Kitagawa, 2017	Low	Low	Low	Low	Low	Low
Klek, 2008a	Low	Low	Low	Some concerns	Low	Some concerns
Klek, 2008b	Low	Low	Low	Low	Low	Low
Klek, 2011a	Low	Low	Low	Some concerns	Low	Some concerns
Klek, 2011b	Low	Low	Low	Low	Low	Low
Klek, 2014	Low	Low	Low	Low	Low	low
Klek, 2017	Low	Low	Low	Low	Low	Low
Lee, 2023a	Low	Low	Low	Low	Low	Low
Lee, 2023b	Low	Low	Low	Some concerns	Low	Some concerns
Lewis, 2018	Low	Low	Low	Low	Low	Low
Li, 2007	Some concerns	Low	Low	Some concerns	Low	Some concerns
Li, 2020a	Some concerns	Low	Low	Some concerns	Low	Some concerns
Li, 2020b	Low	Low	Low	Low	Low	Low
Li, 2020c	Low	Some concerns	Low	Low	Low	Some concerns

Li, 2021	Low	Low	Low	Low	Low	Low
Liang, 2008	Low	Low	Low	Low	Low	Low
Liu, 2012	Low	Low	Low	Some concerns	Low	Some concerns
Liu, 2011a	Low	Low	Low	Low	Low	Low
Liu, 2011b	Low	Low	Low	Some concerns	Low	Some concerns
Lobo, 2006	Low	Low	Low	Low	Low	Low
Luo, 2018	Low	Low	Low	Some concerns	Low	Some concerns
Ma, 2023	Low	Low	Low	Low	Low	Low
Ma, 2015	Low	Low	Low	Low	Low	Low
Manzanares, 2016	Low	Low	Low	Low	Low	Low
Manzanares, 2017	Low	Low	Low	Low	Low	Low
Marano, 2013	Low	Low	Low	Low	Low	Low
Martin, 2017	High	Some concerns	Low	Some concerns	Low	High
Matsuda, 2016	Low	Some concerns	Low	Low	Some concerns	Some concerns
Matsuda, 2006	Low	Low	Low	Low	Low	Low
Matsuda, 2017	Low	Low	Low	Low	Low	Low
McCarter, 1998	Low	Low	Low	Low	Low	Low
Mikagi, 2011	Low	Some concerns	Low	Some concerns	Low	Some concerns
Moriya, 2014	Some concerns	Some concerns	Low	Some concerns	Some concerns	Some concerns
Moya, 2016a	Low	Low	Low	Low	Low	Low
Moya, 2016b	Low	Low	Low	Low	Low	Low
Mudge, 2016	Low	Low	Low	Low	Some concerns	Some concerns
Mudge, 2018	Low	Low	Low	Low	Low	Low
Okamoto, 2009	Low	Low	Low	Some concerns	Low	Some concerns
Panova, 2015	Low	Low	Low	Low	Low	Low
Sakurai, 2007	Low	Low	Low	Low	Low	Low
Scislo, 2018	Low	Low	Low	Low	Low	Low
Senkal, 1997	Low	Low	Low	Low	Low	Low
Senkal, 1999	Low	Low	Low	Low	Low	Low
Shibata, 2011	Low	Low	Low	Low	Low	Low
Slotwinski, 2007a	Some concerns	Low	Low	Low	Low	Some concerns
Slotwinski, 2007b	Low	Low	Low	Low	Low	Low
Slotwinski, 2008	Low	Low	Low	Low	Low	Low

Slotwinski, 2011	Low	Low	Low	Low	Low	Low
Sorensen, 2014	Low	Low	Low	Low	Low	Low
Sultan, 2012	Low	Low	Low	Low	Low	Low
Suzuki, 2010	Low	Low	Low	Low	Low	Low
Vidal Casariego, 2023	Low	Low	Low	Low	Low	Low
Wei, 2014	Low	Low	Low	Low	Low	Low
Wendel, 2007	Low	Low	Low	Low	Low	Low
Wu, 2001	Low	Low	Low	Low	Low	Low
Xu, 2006	Low	Low	Low	Low	Low	Low
Xu, 2022	Low	Low	Low	Low	Low	Low
Yildiz, 2016	Low	Low	Low	Low	Low	Low

Supplementary Table 5: Certainty assessment by GRADE

Certainty assessment							Summary of findings				
Participants (studies) Follow-up	Risk of bias	Inconsistency	Indirectness	Imprecision	Publication bias	Overall certainty of evidence	Study event rates (%)		Relative effect (95% CI)	Anticipated absolute effects	
							With control	With immunonutrition		Risk with control	Risk difference with immunonutrition
**In-hospital mortality**											
534 (4 RCTs)	not serious	not serious	serious[a,b]	not serious	none	⊕⊕⊕◯ Moderate[a,b]	3/263 (1.1%)	4/271 (1.5%)	**OR 1.25** (0.84 to 1.86)	3/263 (1.1%)	**3 fewer per 1000** (from 2 fewer to 10 more)
**1-year overall survival**											
216 (3 RCTs)	not serious	serious[a,b,c]	serious[a,b,c]	serious[a,b,c]	none	⊕◯◯◯ Very low[a,b,c]	70/108 (64.8%)	74/108 (68.5%)	**OR 1.02** (0.60 to 1.73)	70/108 (64.8%)	**5 more per 1000** (from 123 fewer to 113 more)
**Anastomotic leakage**											
1611 (12 RCTs)	not serious	not serious	serious[a,b]	not serious	none	⊕⊕⊕◯ Moderate[a,b]	72/794 (9.1%)	47/817 (5.8%)	**OR 0.62** (0.50 to 0.76)	72/794 (9.1%)	**32 fewer per 1000** (from 43 fewer to 20 fewer)
**Infectious complication**											
2390 (14 RCTs)	not serious	serious[a,b]	serious[a,b]	not serious	publication bias strongly suspected[d]	⊕◯◯◯ Very low[a,b,d]	277/1136 (24.4%)	211/1254 (16.8%)	**OR 0.51** (0.32 to 0.81)	277/1136 (24.4%)	**103 fewer per 1000** (from 150 fewer to 37 fewer)
**Respiratory infection**											

Certainty assessment							Summary of findings				
583 (7 RCTs)	not serious	not serious	serious[a,b]	not serious	none	⨁⨁⨁◯ Moderate[a,b]	25/293 (8.5%)	12/290 (4.1%)	**OR 0.46** (0.33 to 0.64)	25/293 (8.5%)	**44 fewer per 1000** (from 55 fewer to 29 fewer)
**Urinary tract infection**											
1636 (14 RCTs)	not serious	not serious	serious[a,b]	not serious	none	⨁⨁⨁◯ Moderate[a,b]	32/814 (3.9%)	17/822 (2.1%)	**OR 0.58** (0.38 to 0.89)	32/814 (3.9%)	**16 fewer per 1000** (from 24 fewer to 4 fewer)
**Wound infection**											
1543 (16 RCTs)	not serious	not serious	serious[a,b]	not serious	none	⨁⨁⨁◯ Moderate[a,b]	82/755 (10.9%)	60/788 (7.6%)	**OR 0.67** (0.46 to 0.98)	82/755 (10.9%)	**33 fewer per 1000** (from 56 fewer to 2 fewer)
**Sepsis**											
1048 (9 RCTs)	not serious	not serious	serious[a,b]	not serious	none	⨁⨁⨁◯ Moderate[a,b]	26/520 (5.0%)	11/528 (2.1%)	**OR 0.45** (0.28 to 0.70)	26/520 (5.0%)	**27 fewer per 1000** (from 35 fewer to 14 fewer)
**Length of hospital stay (Scale from: 9 to 39.51)**											
1854 (15 RCTs)	not serious	serious[a,b]	serious[a,b]	not serious	none	⨁⨁◯◯ Low[a,b]	911	943	-	911	MD **2.47 day lower** (4.13 lower to 0.8 higher)

CI: confidence interval; **MD:** mean difference; **OR:** odds ratio

Explanations

a. different cancer population

b. difference in disease severity

c. difference in types of immunonutrition

d. larger studies are more asymmetrically distributed

Supplementary Document 1: Individualised search key in different databases

Pubmed

("digestive" OR "gastrointestinal" OR "oesophag*" OR "esophag*" OR "esogastric" OR "gastroesophageal" OR "stomach" OR "gastric" OR "cardia" OR "antrum" OR "gut" OR "bowel" OR "duodenum" OR "ileum" OR "jejunum" OR "colon*" OR "colo*" OR "rectum" OR "rectal" OR "appendix" OR "cecum" OR "cecal" OR "colorectal" OR "biliary" OR "gallbladder" OR "cholecyst" OR "pancreas" OR "liver" OR "hepat*" OR "cholangio*" OR "bile duct") AND ("Neoplasm" OR "neoplas*" OR "cancer" OR "cancers" OR "malignancy" OR "malignancies" OR "malignant" OR "tumor" OR "tumors" OR "tumour" OR "carcinoma" OR "adenocarcinoma") AND ("immunonutrition" OR "immuno-nutrition" OR "immuno nutrition" OR "immune nutrition" OR "immunonutrient" OR "immunonutri*" OR "immune-enhanced" OR "immune enhanced" OR "immune-enhancing" OR "immune enhancing" OR "immune-modulating" OR "immunemodulating" OR "immunomodulating" OR "immune-modulating" OR "immune modulating" OR "immunomodulatory" OR "pharmaconutrition" OR "pharmaconutri*" OR "pharmacological nutrition")

Embase

((digestive OR gastrointestinal OR oesophag* OR esophag* OR esogastric OR gastroesophageal OR stomach OR gastric OR cardia OR antrum OR gut OR bowel OR duodenum OR ileum OR jejunum OR colon* OR colo* OR rectum OR rectal OR appendix OR cecum OR cecal OR colorectal OR biliary OR gallbladder OR cholecyst OR pancreas OR liver OR hepat* OR cholangio* OR 'bile duct') AND (neoplasm OR neoplas* OR cancer OR cancers OR malignancy OR malignancies OR malignant OR tumor OR tumors OR tumour OR carcinoma OR adenocarcinoma) AND (immunonutrition OR 'immuno-nutrition' OR 'immuno nutrition' OR 'immune nutrition' OR immunonutrient OR immunonutri* OR 'immune-enhanced' OR 'immune enhanced' OR 'immune enhancing' OR 'immune-enhancing' OR immunemodulating OR 'immune modulating' OR immunemodulating OR immunoemodulating OR 'immune-modulating' OR 'immune modulating' OR immunomodulatory OR pharmaconutrition OR pharmaconutri* OR 'pharmacological nutrition')):ab,kw,ti

Cochrane

(digestive OR gastrointestinal OR oesophag* OR esophag* OR esogastric OR gastroesophageal OR stomach OR gastric OR cardia OR antrum OR gut OR bowel OR duodenum OR ileum OR jejunum OR colon* OR colo* OR rectum OR rectal OR appendix OR cecum OR cecal OR colorectal OR biliary OR gallbladder OR cholecyst OR pancreas OR liver OR hepat* OR cholangio* OR 'bile duct') AND (neoplasm OR neoplas* OR cancer OR cancers OR malignancy OR malignancies OR malignant OR tumor OR tumors OR tumour OR carcinoma OR adenocarcinoma) AND (immunonutrition OR immuno-nutrition OR " immuno nutrition" OR " immune nutrition" OR immunonutrient OR immunonutri* OR immune-enhanced OR " immune enhanced" OR immune-enhancing OR " immune enhancing" OR immune-modulating OR immunemodulating OR immunomodulating OR immune-modulating OR " immune modulating" OR immunomodulatory OR pharmaconutrition OR pharmaconutri* OR "pharmacological nutrition")

Supplementary Document 2: Detailed strategy for overlapping population

Outcome	Author	DOI number	Strategy
anastomotic leakage	Mudge, 2018	10.1002/bjs.10923	Data on the preoperative intervention arm
	Braga, 2002	10.1067/msy.2002.128350	used Braga, 2002 (doi: 10.1067/msy.2002.128350) with colorectal cancer
	Braga, 2002	NA	
	Braga, 2005	10.1177/01486071050290S1S57	
	Lewis, 2018	NA	Data from intent to treat analysis
	Suzuki, 2010	10.1016/j.surg.2010.01.017	Data on the preoperative intervention arm
	Takeuchi, 2007	10.1007/s00268-007-9219-8	Data on the perioperative intervention arm
	Moya, 2016	10.1007/s00464-016-4836-7	used Moya, 2016 (doi:10.1097/MD0000000000003704) higher population number
	Moya, 2016	10.1097/MD0000000000003704	
	Sultan, 2012	10.1002/bjs.7799	Data where 'standard enteral nutrition' was the control
infectious complication	Bozetti, 2007	doi:10.1016/j.clnu.2007.06.009	Data where 'enteral nutrition' was the control
	Giger-Pabst, 2013	NA	Data from intent to treat analysis
	Heslin, 1997	NA	Data from intent to treat analysis
	Mudge, 2018	10.1002/bjs.10923	Data on the preoperative intervention arm
	Suzuki, 2010	10.1016/j.surg.2010.01.017	Data on the preoperative intervention arm
	Braga, 1998	10.1097/00003246-199801000-00012	used Braga, 2005, with a higher population number
	Braga, 1999	NA	
	Braga, 2005	10.1177/01486071050290S1S57	
	Braga, 2002	10.1067/msy.2002.128350	
	Braga, 2002	NA	
	Sultan, 2012	10.1002/bjs.7799	Data where 'enteral nutrition' was the control
non-infectious complication	Bozetti, 2007	doi:10.1016/j.clnu.2007.06.009	Data where 'enteral nutrition' was the control
	Gunerhan, 2009	doi:10.3748/wjg.15.467	Data where 'standard enteral nutrition' was the control
	Suzuki, 2010	10.1016/j.surg.2010.01.017	Data on the preoperative intervention arm

	Braga, 1999	NA	used Braga, 2005, with a higher population number
	Braga, 2002	10.1067/msy.2002.128350	
	Braga, 2002	NA	
	Braga, 1998	10.1097/00003246-199801000-00012	
	Braga, 2005	10.1177/01486071050290S1S57	
respiratory infection	Braga, 2002	10.1067/msy.2002.128350	used Braga, 2005, with a higher population number
	Braga, 2002	NA	
	Braga, 2005	10.1177/01486071050290S1S57	
	Mudge, 2018	10.1002/bjs.10923	Data on the preoperative intervention arm
sepsis	Giger-Pabst, 2013	NA	Data from intent to treat analysis
	Gunerhan, 2009	doi:10.3748/wjg.15.467	Data where ‘standard enteral nutrition’ was the control
	Lewis, 2018	NA	Data from intent to treat analysis
	Mudge, 2018	10.1002/bjs.10923	Data on the preoperative intervention arm
	Sultan, 2012	10.1002/bjs.7799	Data where ‘standard enteral nutrition’ was the control
	Moriya, 2014	NA	Data where the intervention is 750 ml
urinary tract infection	Giger-Pabst, 2013	NA	Data from intent to treat analysis
	Gunerhan, 2009	doi:10.3748/wjg.15.467	Data where ‘standard enteral nutrition’ was the control
	Lewis, 2018	NA	Data from intent to treat analysis
	Mudge, 2018	10.1002/bjs.10923	Data on the preoperative intervention arm
	Braga, 2002	10.1067/msy.2002.128350	used Braga, 2005, with a higher population number
	Braga, 2002	NA	
	Braga, 1998	10.1097/00003246-199801000-00012	
	Braga, 1999	NA	
	Braga, 2005	10.1177/01486071050290S1S57	
	Sultan, 2012	10.1002/bjs.7799	Data where ‘standard enteral nutrition’ was the control
wound infection	Giger-Pabst, 2013	NA	Data from intent to treat analysis

	Gunerhan, 2009	doi:10.3748/wjg.15.467	Data where 'standard enteral nutrition' was the control
	Lewis, 2018	NA	Data from intent to treat analysis
	Mudge, 2018	10.1002/bjs.10923	Data on the preoperative intervention arm
	Braga, 2002	10.1067/msy.2002.128350	used Braga, 2005, with a higher population number
	Braga, 2002	NA	
	Braga, 1998	10.1097/00003246-199801000-00012	
	Braga, 1999	NA	
	Braga, 2005	10.1177/01486071050290S1S57	
Mortality	Braga, 2002	10.1067/msy.2002.128350	used Braga, 2002, (doi:NA) because it investigates GI cancer, and not only colorectal
	Braga, 2002	NA	
	Braga, 2002	NA	used data where immunonutrition was administered preoperatively
	Mudge, 2018	10.1002/bjs.10923	Data on the preoperative intervention arm
	Suzuki, 2010	10.1016/j.surg.2010.01.017	Data on the preoperative intervention arm
	Lewis, 2018	NA	Data from intent to treat analysis
Surgical site infection			
	Sultan, 2012	10.1002/bjs.7799	Data where 'standard enteral nutrition' was the control
serum albumin	Braga 1999	NA	used Braga, 1999, with a higher population number
	Braga 1995	10.1159/000223143	
	Słotwiński, 2007	NA	choose Słotwiński 2008 – with the latest data
	Słotwiński, 2008	NA	
serum CRP	Braga 1999	NA	used Braga, 1999, with a higher population number
	Braga 1995	10.1159/000223143	
	Sultan, 2012	10.1002/bjs.7799	Data where 'standard enteral nutrition' was the control
serum CD4/CD8	Słotwiński, 2007	NA	choose Słotwiński 2008 – with the latest data
	Słotwiński, 2008	NA	
	Braga, 1998	10.1097/00003246-199801000-00012	

	Braga, 1999	NA	used Braga, 1999, with a higher population number
serum interleukin-6	Braga, 2002	10.1067/msy.2002.128350	used Braga, 2002, with a higher population number
	Braga, 1998	10.1097/00003246-199801000-00012	
	Gianotti, 1999	10.1177/0148607199023006314	used Gianotti 1997, with a higher population number
	Gianotti, 1997	10.1001/archsurg.1997.01430350072012	
	Li, 2020	NA	used Li, 2021
	Li, 2021	10.21037/apm-20-1399	
	Słotwiński, 2007	NA	choose Słotwiński 2008 – with the latest data
	Słotwiński, 2008	NA	
serum prealbumin	Gunerhan, 2009	doi:10.3748/wjg.15.467	Data where ‘standard enteral nutrition’ was the control
	Braga, 1998	10.1097/00003246-199801000-00012	used Braga, 1999, with a higher population number
	Braga, 1999	NA	
	Gianotti, 1999	10.1177/0148607199023006314	used Gianotti 1997, with a higher population number
	Gianotti, 1997	10.1001/archsurg.1997.01430350072012	
length of hospital stay	Braga, 2002	10.1067/msy.2002.128350	used Braga, 2005, with a higher population number
	Braga, 2002	NA	
	Braga, 1998	10.1097/00003246-199801000-00012	
	Braga 1995	10.1159/000223143	
	Braga, 2005	10.1177/01486071050290S1S57	
	Gunerhan, 2009	doi:10.3748/wjg.15.467	Data where ‘standard enteral nutrition’ was the control
	Mudge, 2018	10.1002/bjs.10923	Data on the preoperative intervention arm
	Sultan, 2012	10.1002/bjs.7799	Data where ‘standard enteral nutrition’ was the control

Supplementary Document 3: Definition of non-infection complications of each study

Study	Definition
Okamoto, 2009	• **Cardiac dysfunction**<br>• **Intestinal obstruction**<br>• **Edematous of anastomosis**<br>• **Bleeding**
Mudge, 2018	• **Acute respiratory distress syndrome**<br>• **Anastomotic leak**<br>• **Chyle leak**<br>• **Others**
Suzuki, 2010	• **Anastomotic leakage**<br>• **Atelectasis**<br>• **Liver infarction**
Mikagi, 2011	• **Ileus**<br>• **Atelectasis**
Ciacio, 2021	NA
Giger, 2007	NA
Braga, 2005	NA
Bozzeti, 2007	• **Wound dehiscence** (Any dehiscence of the fascia longer than 3 cm)<br>• **Bleeding** (Necessity of blood transfusion ≥2 units)<br>• **Anastomotic leak** (Any dehiscence with clinical and radiologic evidence)<br>• **Respiratory failure** (Presence of dyspnea and respiratory rate >35/min or PaO_2 <70mmHg)<br>• **Circulatory insufficiency** (Unstable blood pressure requiring use of extra fluids and/or cardiac stimulants)<br>• **Renal dysfunction** (Increased serum urea and/or creatinine level (50% above baseline))<br>• **Renal failure** (Necessary of hemodialysis)<br>• **Hepatic dysfunction** (Increased serum bilirubin level<br>• (50% above baseline))<br>• **Pancreatic fistula** (Daily output of fluid >10 mL from surgical drainage with amylase level 5 times higher than serum concentration)<br>• **Delayed gastric emptying** (Necessity of naso-gastric suction for more than 8 days after surgery)<br>• **Multiple Organ Dysfunction Syndrome** (A state of physiological derangement in which organ function is not capable of maintaining homeostasis)
Giger-Pabst, 2013	• **Sterile pancreatic fistula**<br>• **Presacral sterile hematoma**

	• **Heart failure**<br>• **Myocardial infection**<br>• **Pulmonary embolism**<br>• **Pleural effusion**<br>• **Transient renal failure**<br>• **Anastomotic rectal bleeding**<br>• **Delayed gastric emptying**<br>• **Anastomotic insufficiency**<br>• **Aspiration /Adult respiratory distress syndrome**
Gunerhan, 2009	NA

NA= Not available

Supplementary Document 4: Meta-regression analysis

1. Meta-regression analysis for **serum albumin level** in gastrointestinal cancer patients (intervention: arginine, nucleotides, omega-3 fatty acids)

Results Table

**Slope**	0.20 g/l/days
**Slope SE**	0.14 g/l/days
**Intercept**	-0.76
**Intercept SE**	0.71
**p-value**	0.2235

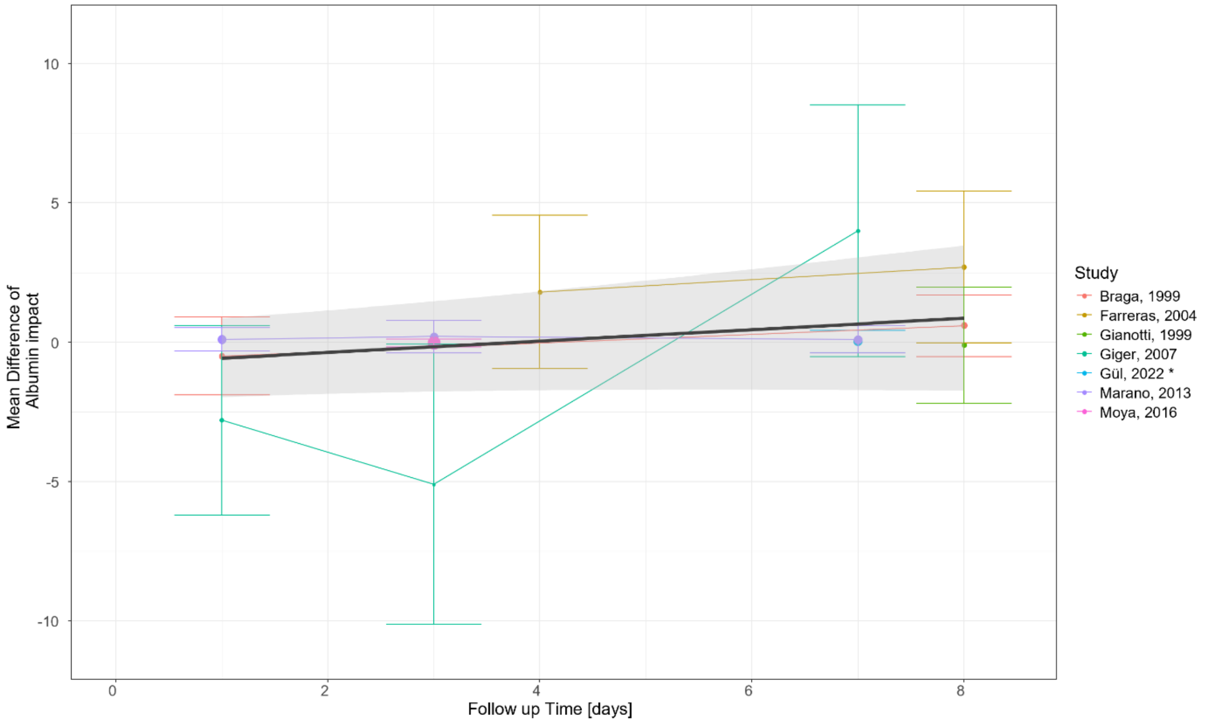

Leave-one-out analysis

Study	Slope	Slope SE	Intercept	Intercept SE	p-value
Omitting Braga, 1999	0.31 g/l/days	0.26 g/l/days	-1.24	1.41	0.3194
Omitting Farreras, 2004	0.10 g/l/days	0.07 g/l/days	-0.34	0.34	0.3368
Omitting Gianotti, 1999	0.27 g/l/days	0.18 g/l/days	-1.00	0.88	0.2177
Omitting Giger, 2007	0.06 g/l/days	0.05 g/l/days	-0.15	0.20	0.3255
Omitting Gül, 2022 *	0.27 g/l/days	0.18 g/l/days	-0.97	0.88	0.2191
Omitting Marano, 2013	0.39 g/l/days	0.25 g/l/days	-1.75	1.42	0.2162
Omitting Moya, 2016	0.26 g/l/days	0.19 g/l/days	-1.05	1.06	0.2531

2. Meta-regression analysis for **serum CD4+/CD8+ ratio level** in gastrointestinal cancer patients (intervention: arginine, nucleotides, omega-3 fatty acids)

Results Table

**Slope**	0.04
**Slope SE**	0.03
**Intercept**	0.24
**Intercept SE**	0.22
**p-value**	0.3377

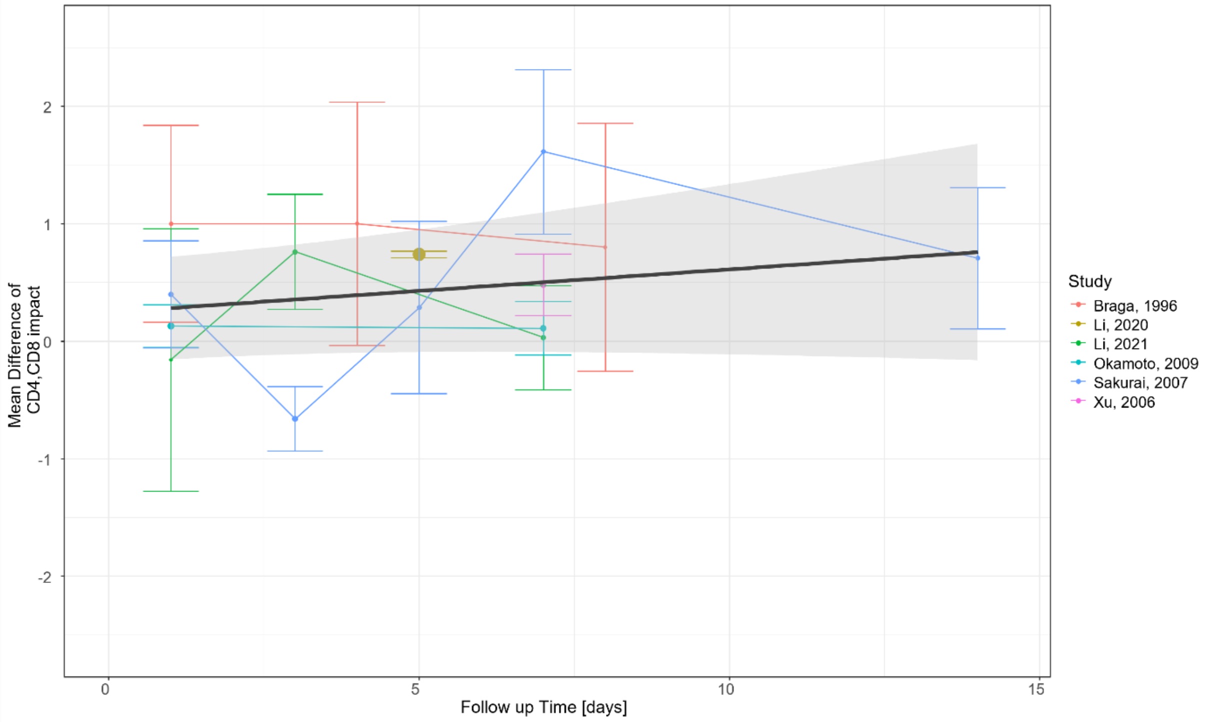

Leave-one-out analysis

Study	Slope	Slope SE	Intercept	Intercept SE	p-value
Omitting Braga, 1996	0.05	0.02	0.11	0.16	0.2109
Omitting Li, 2020	0.04	0.03	0.18	0.22	0.3637
Omitting Li, 2021	0.04	0.03	0.24	0.25	0.3133
Omitting Okamoto, 2009	0.04	0.04	0.32	0.32	0.5002
Omitting Sakurai, 2007	0.001	0.03	0.48	0.29	0.9869
Omitting Xu, 2006	0.04	0.03	0.24	0.22	0.3517

3. Meta-regression analysis for **serum CRP level** in gastrointestinal cancer patients (intervention: arginine, nucleotides, omega-3 fatty acids)

Results Table

**Slope**	-0.16 mg/l/days
**Slope SE**	0.84 mg/l/days
**Intercept**	-23.12
**Intercept SE**	11.41
**p-value**	0.8611

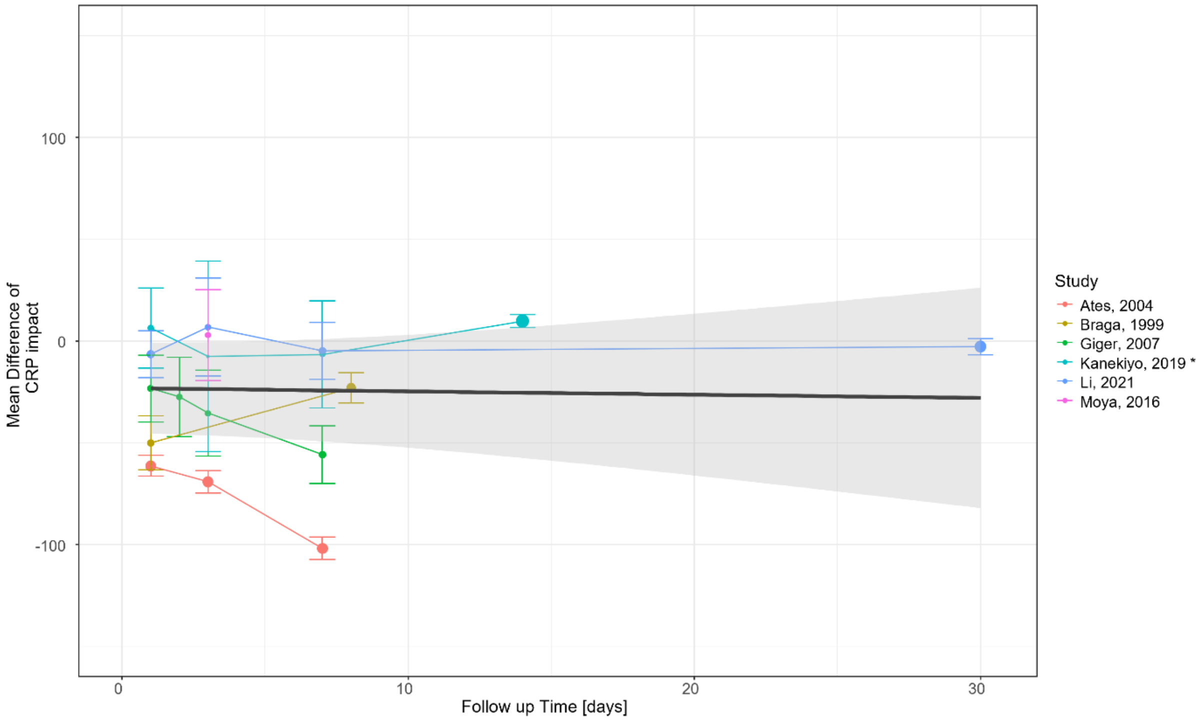

Leave-one-out analysis

Study	Slope	Slope SE	Intercept	Intercept SE	p-value
Omitting Ates, 2004	0.33 mg/l/days	0.45 mg/l/days	-15.84	10.36	0.5551
Omitting Braga, 1999	- 0.76 mg/l/days	1.19 mg/l/days	-16.75	12.20	0.5938
Omitting Giger, 2007	0.21 mg/l/days	0.59 mg/l/days	-22.92	14.36	0.7596
Omitting Kanekiyo, 2019 *	-0.26 mg/l/days	1.37 mg/l/days	-28.90	11.80	0.8693

Omitting Li, 2021	-1.04 mg/l/days	2.07 mg/l/days	-24.59	12.70	0.6552
Omitting Moya, 2016	-0.12 mg/l/days	0.84 mg/l/days	-28.09	12.42	0.9011

4. Meta-regression analysis for **serum CRP level** in gastrointestinal cancer patients (intervention: omega-3 fatty acids)

Results Table

**Slope**	-1.22 mg/l/days
**Slope SE**	2.71 mg/l/days
**Intercept**	-1.48
**Intercept SE**	17.82
**p-value**	0.6964

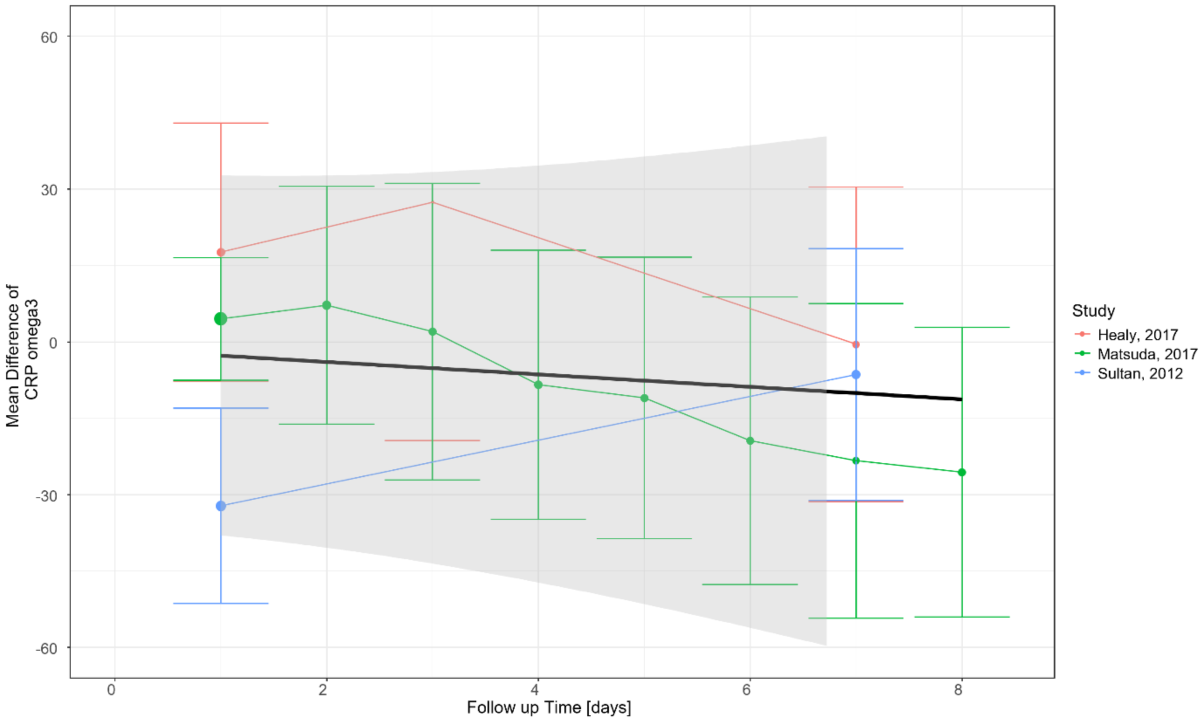

Leave-one-out analysis

Study	Slope	Slope SE	Intercept	Intercept SE	p-value
Omitting Healy, 2017	-0.40 mg/l/days	4.37 mg/l/days	-12.93	23.33	0.9414
Omitting Matsuda, 2017	0.64 mg/l/days	3.71 mg/l/days	-6.81	30.24	0.8907
Omitting Sultan, 2012	-3.74 mg/l/days	0.76 mg/l/days	15.37	6.58	0.1258

5. Meta-regression analysis for **serum interleukin-6 level** in gastrointestinal cancer patients (intervention: arginine, nucleotides, omega-3 fatty acids)

Results Table

**Slope**	-1.09 pg/ml/days
**Slope SE**	3.18 pg/ml/days
**Intercept**	-39.88
**Intercept SE**	17.81
**p-value**	0.7686

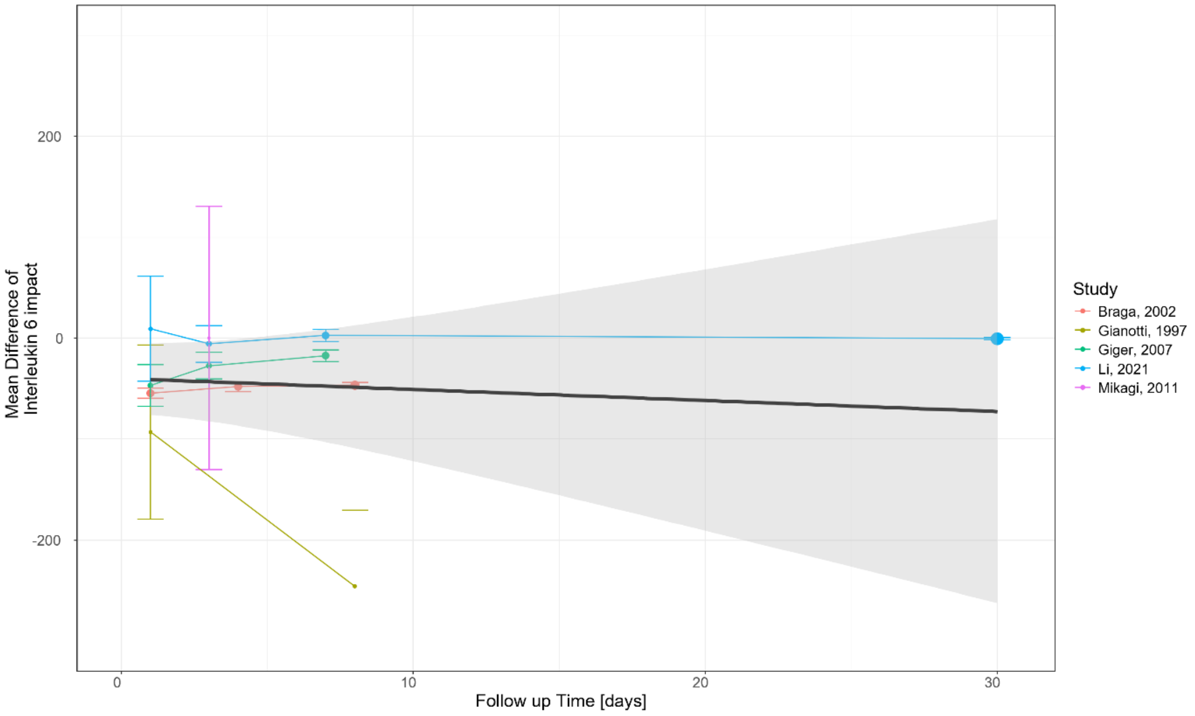

Leave-one-out analysis

Study	Slope	Slope SE	Intercept	Intercept SE	p-value
Omitting Braga, 2002	-1.38 pg/ml/days	4.30 pg/ml/days	-36.34	23.84	0.7890
Omitting Gianotti, 1997	0.75 pg/ml/days	0.92 pg/ml/days	-24.73	16.66	0.5277

Omitting Giger, 2007	-1.78 pg/ml/days	4.59 pg/ml/days	-37.91	23.29	0.7482
Omitting Li, 2021	-6.31 pg/ml/days	8.78 pg/ml/days	-40.31	18.69	0.5463
Omitting Mikagi, 2011	-1.12 pg/ml/days	3.17 pg/ml/days	-49.21	19.17	0.7636

6. Meta-regression analysis for **serum interleukin-6 level** in gastrointestinal cancer patients (intervention: omega-3 fatty acids)

Results Table

**Slope**	-13.45 pg/ml/days
**Slope SE**	21.20 pg/ml/days
**Intercept**	-39.81
**Intercept SE**	29.00
**p-value**	0.5908

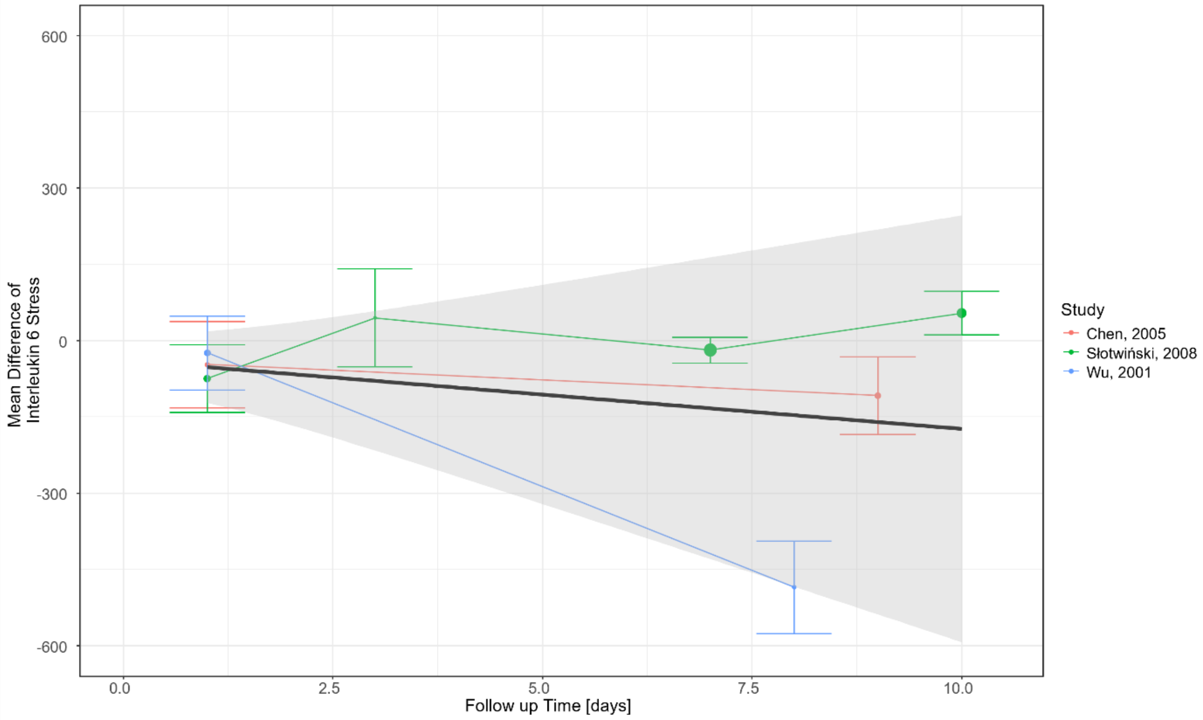

Leave-one-out analysis

Study	Slope	Slope SE	Intercept	Intercept SE	p-value
Omitting Chen, 2005	-18.00 pg/ml/days	38.37 pg/ml/days	-34.93	57.27	0.7202
Omitting Słotwiński, 2008	-31.19 pg/ml/days	28.69 pg/ml/days	-17.91	40.04	0.4732
Omitting Wu, 2001	2.79 pg/ml/days	8.08 pg/ml/days	-39.33	5.08	0.7881

7. Meta-regression analysis for **serum total lymphocyte level** in gastrointestinal cancer patients (intervention: arginine, nucleotides, omega-3 fatty acids)

Results Table

**Slope**	-16.67 cells/mm^3/days
**Slope SE**	24.44 cells/mm^3/days
**Intercept**	-26.13
**Intercept SE**	130.36
**p-value**	0.5506

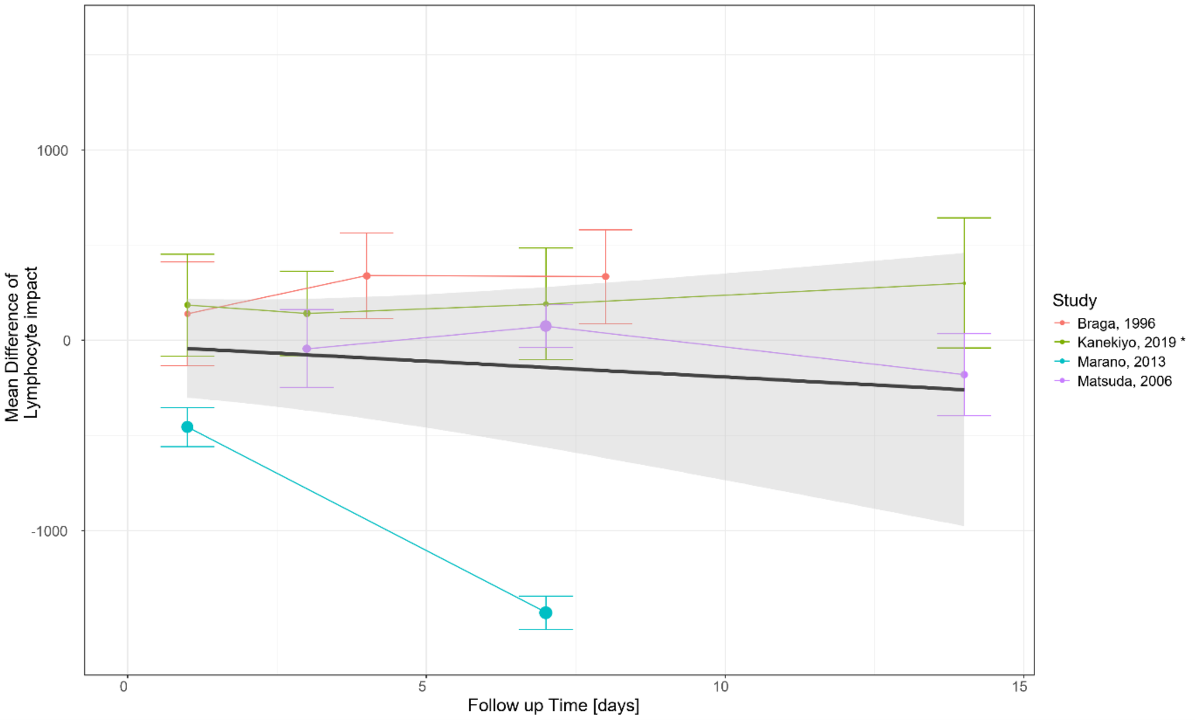

Leave-one-out analysis

Study	Slope	Slope SE	Intercept	Intercept SE	p-value
Omitting Braga, 1996	-25.78 cells/mm3/days	32.07 cells/mm3/days	-77.42	162.89	0.5149
Omitting Kanekiyo, 2019 *	-32.10 cells/mm3/days	36.65 cells/mm3/days	-73.94	160.10	0.4798
Omitting Marano, 2013	3.10 cells/mm3/days	10.58 cells/mm3/days	105.74	41.23	0.7997
Omitting Matsuda, 2006	-20.12 cells/mm3/days	42.80 cells/mm3/days	-33.70	179.88	0.6900

8. Meta-regression analysis for **serum prealbumin level** in gastrointestinal cancer patients (intervention: arginine, nucleotides, omega-3 fatty acids)

Results Table

**Slope**	0.003 g/l/days
**Slope SE**	0.002 g/l/days
**Intercept**	-0.001
**Intercept SE**	0.006
**p-value**	0.1266

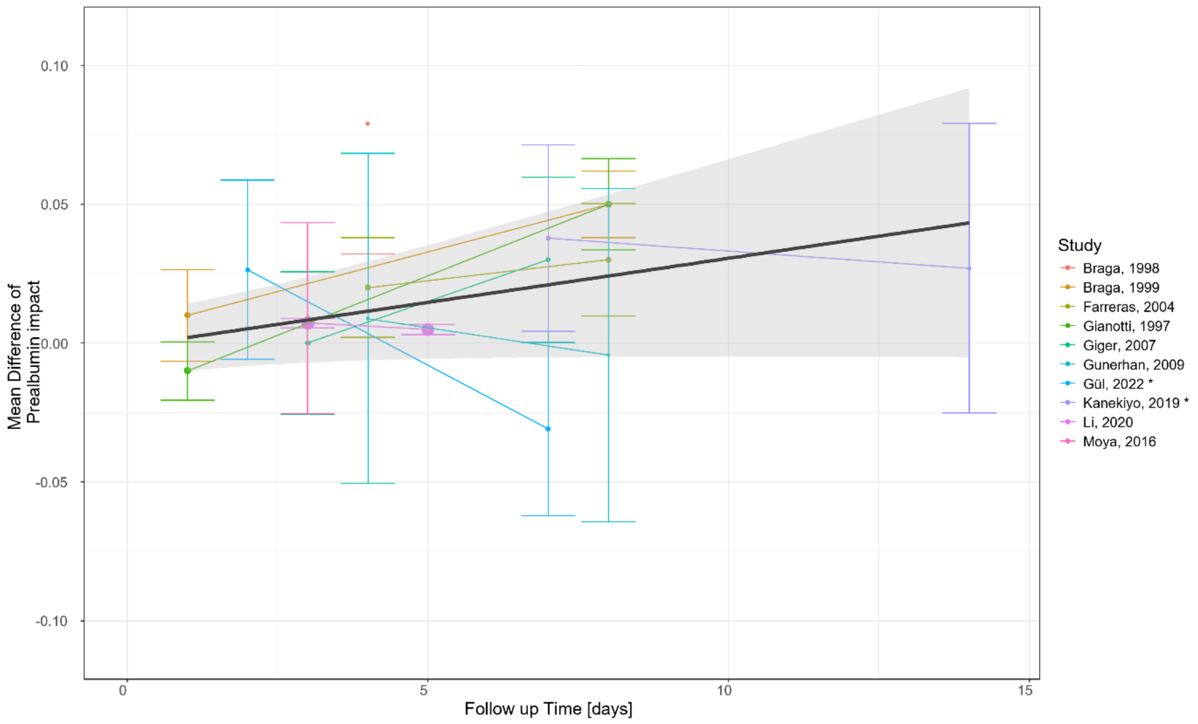

Leave-one-out analysis

Study	Slope	Slope SE	Intercept	Intercept SE	p-value
Omitting Braga, 1998	0.003 g/l/days	0.002 g/l/days	-0.003	0.006	0.12499
Omitting Braga, 1999	0.002 g/l/days	0.002 g/l/days	0.0003	0.008	0.32182
Omitting Farreras, 2004	0.003 g/l/days	0.002 g/l/days	-0.001	0.006	0.17877
Omitting Gianotti, 1997	0.002 g/l/days	0.002 g/l/days	0.004	0.007	0.29571
Omitting Giger, 2007	0.003 g/l/days	0.002 g/l/days	-0.0005	0.006	0.16431
Omitting Gunerhan, 2009	0.003 g/l/days	0.002 g/l/days	-0.002	0.006	0.11860
Omitting Gül, 2022 *	0.004 g/l/days	0.002 g/l/days	-0.004	0.005	0.06417
Omitting Kanekiyo, 2019 *	0.003 g/l/days	0.002 g/l/days	-0.002	0.007	0.20628
Omitting Li, 2020	0.004 g/l/days	0.002 g/l/days	0.002	0.01	0.13740
Omitting Moya, 2016	0.003 g/l/days	0.002 g/l/days	-0.001	0.006	0.12647

9. Meta-regression analysis for **serum transferrin level** in gastrointestinal cancer patients (intervention: arginine, nucleotides, omega-3 fatty acids)

Results Table

**Slope**	-0.31 mg/dl/days
**Slope SE**	0.84 mg/dl/days
**Intercept**	9.91
**Intercept SE**	4.57
**p-value**	0.7292

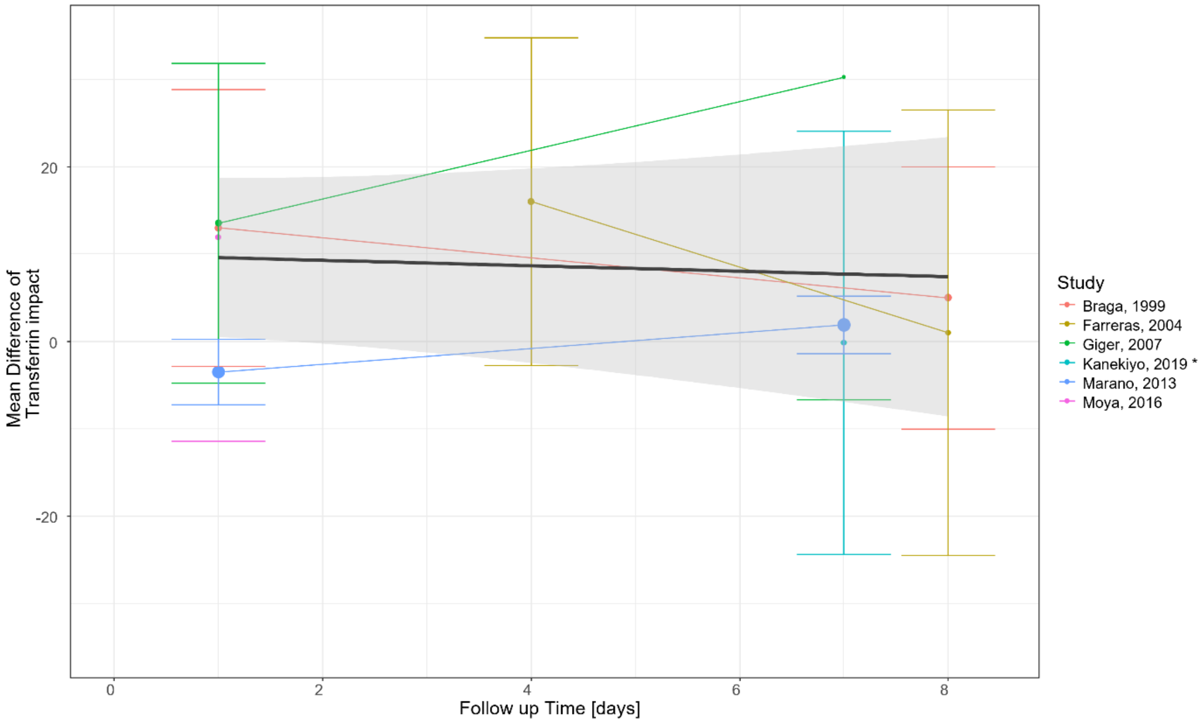

Leave-one-out analysis

Study	Slope	Slope SE	Intercept	Intercept SE	p-value
Omitting Braga, 1999	0.011 mg/dl/days	1.12 mg/dl/days	8.42	5.58	0.9926
Omitting Farreras, 2004	0.28 mg/dl/days	0.93 mg/dl/days	7.26	4.41	0.7873
Omitting Giger, 2007	-0.78 mg/dl/days	0.77 mg/dl/days	9.00	6.25	0.3852
Omitting Kanekiyo, 2019 *	-0.09 mg/dl/days	0.93 mg/dl/days	10.05	4.58	0.9262
Omitting Marano, 2013	-0.82 mg/dl/days	0.93 mg/dl/days	14.91	1.87	0.4426
Omitting Moya, 2016	-0.22 mg/dl/days	0.94 mg/dl/days	9.17	5.89	0.8303

10. Meta-regression analysis for **serum white blood cell level** in gastrointestinal cancer patients (intervention: omega-3 fatty acids)

Results Table

**Slope**	-5.41 10^9/l/days
**Slope SE**	60.60 10^9/l/days
**Intercept**	-229.61
**Intercept SE**	934.39
**p-value**	0.9422

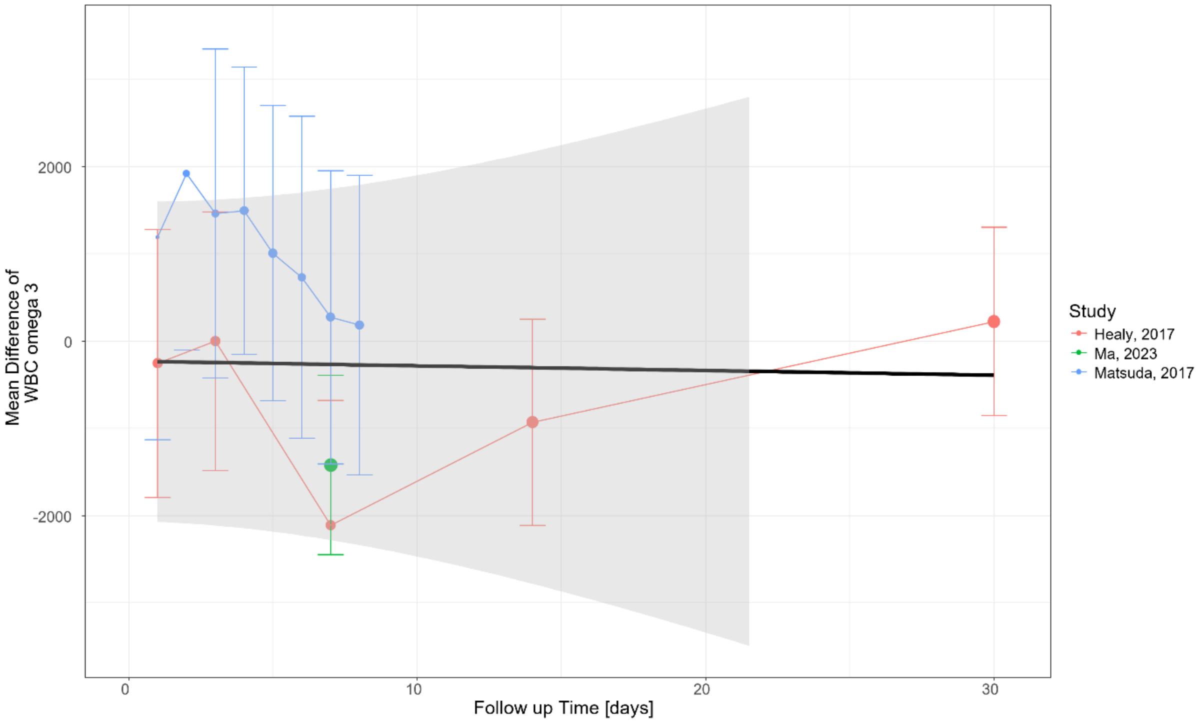

Leave-one-out analysis

Study	Slope	Slope SE	Intercept	Intercept SE	p-value
Omitting Healy, 2017	-174.12 10^9/l/days	241.42 10^9/l/days	737.50	2174.42	0.5962
Omitting Ma, 2023	-13.33 10^9/l/days	49.58 10^9/l/days	202.68	1023.84	0.8280
Omitting Matsuda, 2017	34.30 10^9/l/days	14.85 10^9/l/days	-1187.60	396.04	0.2576

Supplementary Figures 1-29: Individual Forest plots

Figure 1: Pooled analysis of the effect of immunonutrition support (**arginine, nucleotides, omega-3 fatty acids**) on **30-day mortality** in gastrointestinal cancer

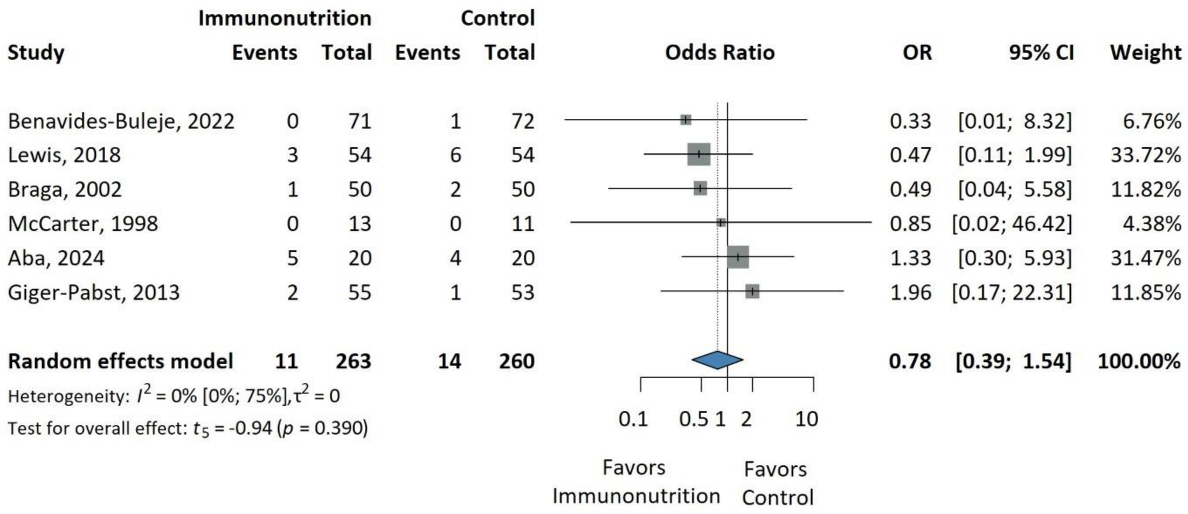

OR=odds ratio, 95% CI= 95% confidence interval

Figure 2: Pooled analysis of the effect of immunonutrition support (**mixed types**) on **30-day mortality** in gastrointestinal cancer

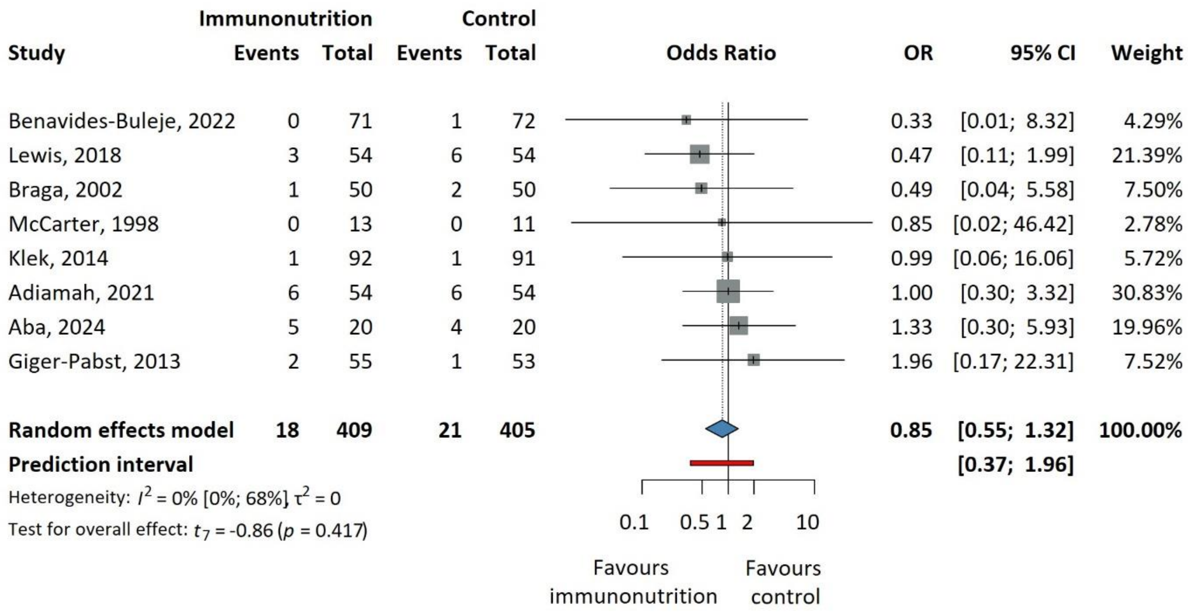

OR=odds ratio, 95% CI= 95% confidence interval

Figure 3: Pooled analysis of the effect of immunonutrition support (**mixed types**) on **1-year mortality** in gastrointestinal cancer

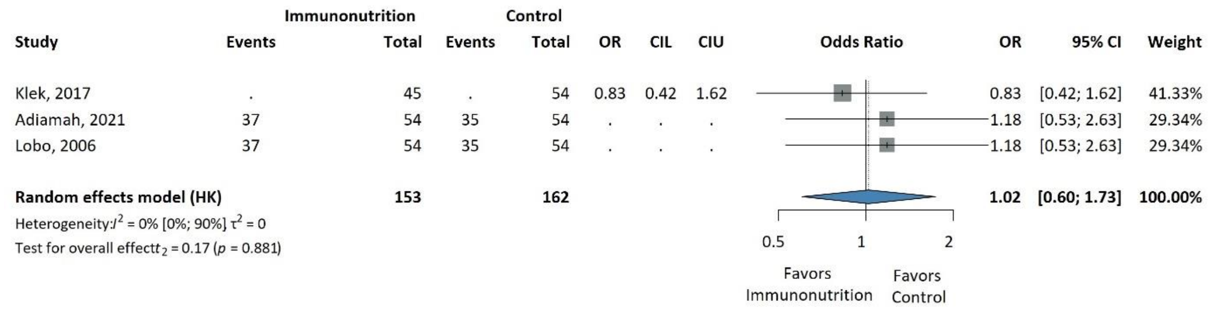

OR=odds ratio, 95% CI= 95% confidence interval

Figure 4: Pooled analysis of the effect of immunonutrition support (**mixed types**) on **3-year mortality** in gastrointestinal cancer

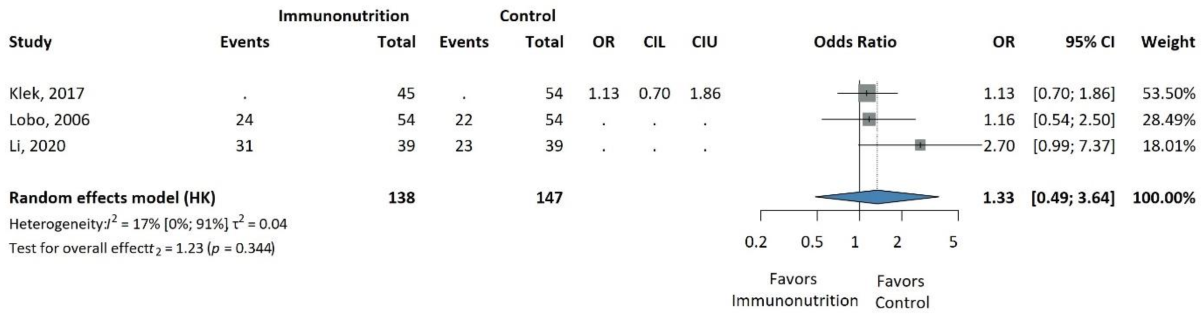

OR=odds ratio, 95% CI= 95% confidence interval

Figure 5: Pooled analysis of the effect of immunonutrition support (**mixed types**) on **5-year mortality** in gastrointestinal cancer

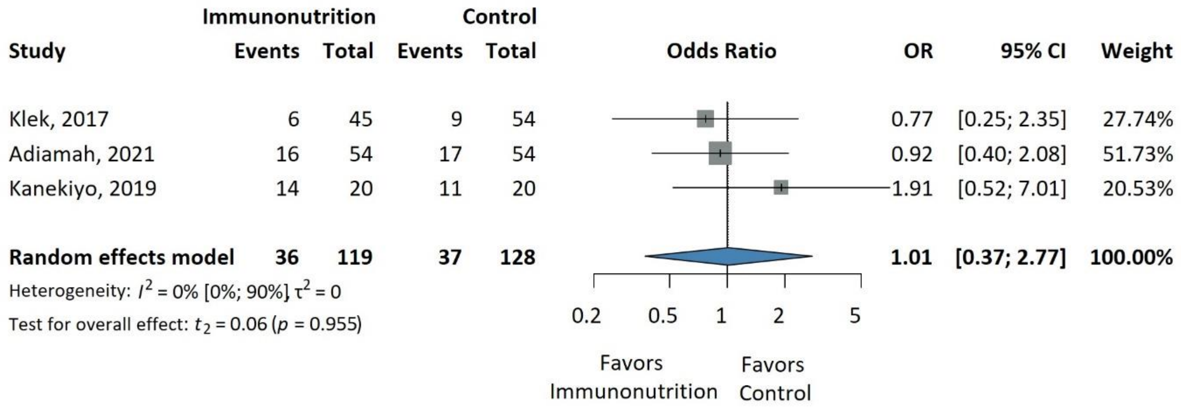

OR=odds ratio, 95% CI= 95% confidence interval

Figure 6: Pooled analysis of the effect of immunonutrition support (**arginine, nucleotides, omega-3 fatty acids**) on **infectious complications** in gastrointestinal cancer

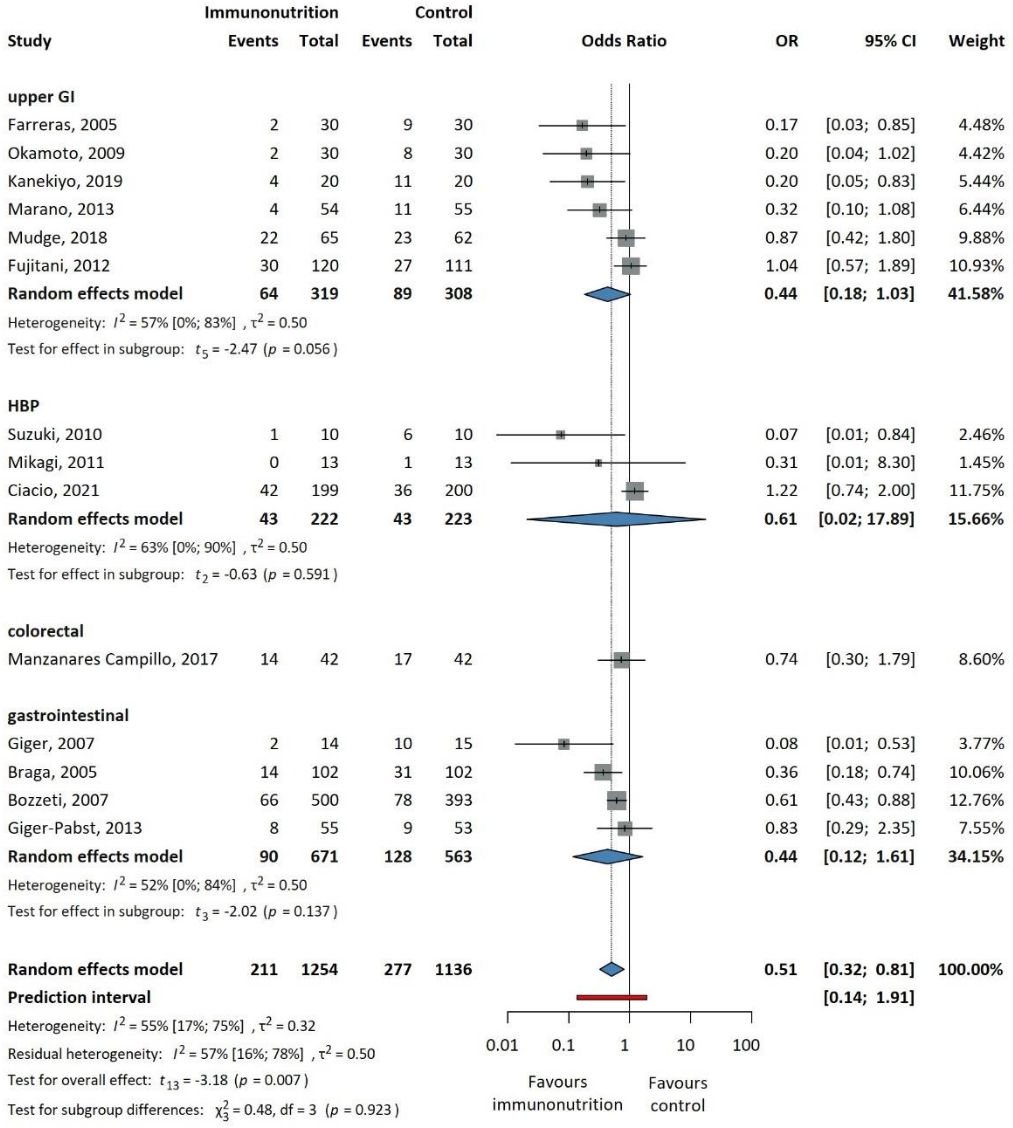

OR=odds ratio, 95% CI= 95% confidence interval, upper GI= upper gastrointestinal, HBP= hepatobiliary-pancreatic

Figure 7: Pooled analysis of the effect of **preoperative** immunonutrition support (**arginine, nucleotides, omega-3 fatty acids**) on **infectious complications** in gastrointestinal cancer

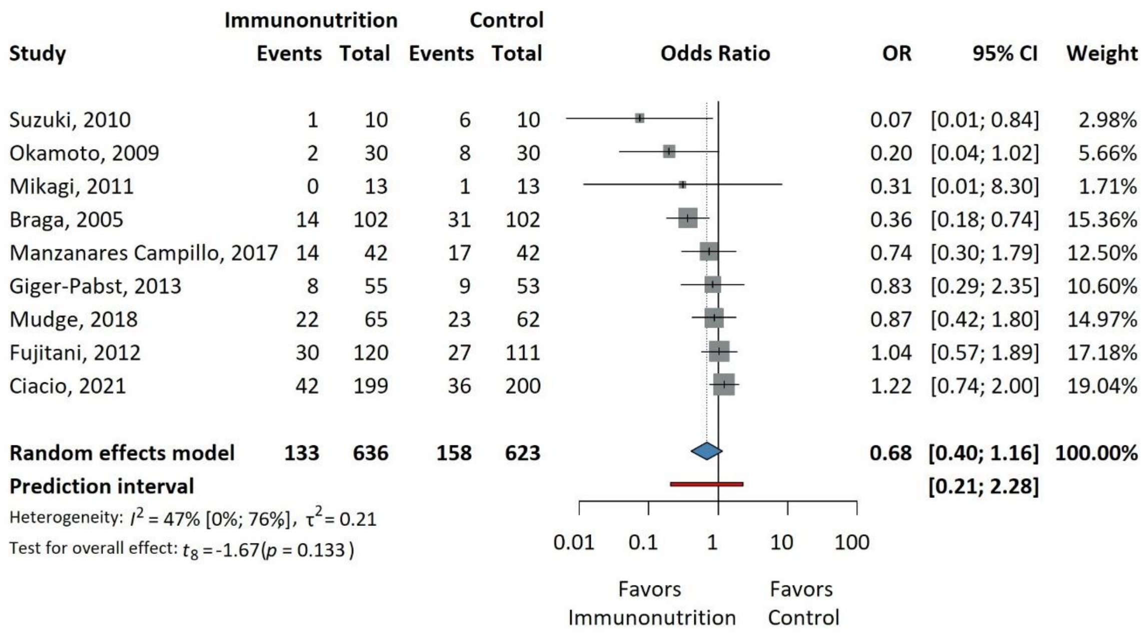

OR=odds ratio, 95% CI= 95% confidence interval,

Figure 8: Pooled analysis of the effect of **postoperative** immunonutrition support (**arginine, nucleotides, omega-3 fatty acids**) on **infectious complications** in gastrointestinal cancer

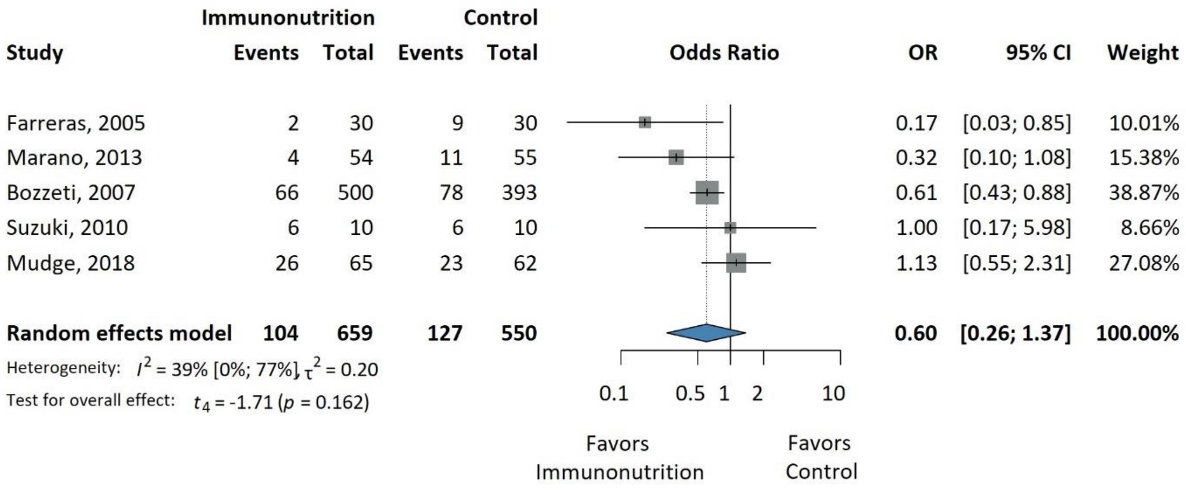

OR=odds ratio, 95% CI= 95% confidence interval

Figure 9: Pooled analysis of the effect of immunonutrition support (**arginine, nucleotides, omega-3 fatty acids**) on **respiratory infection** in gastrointestinal cancer

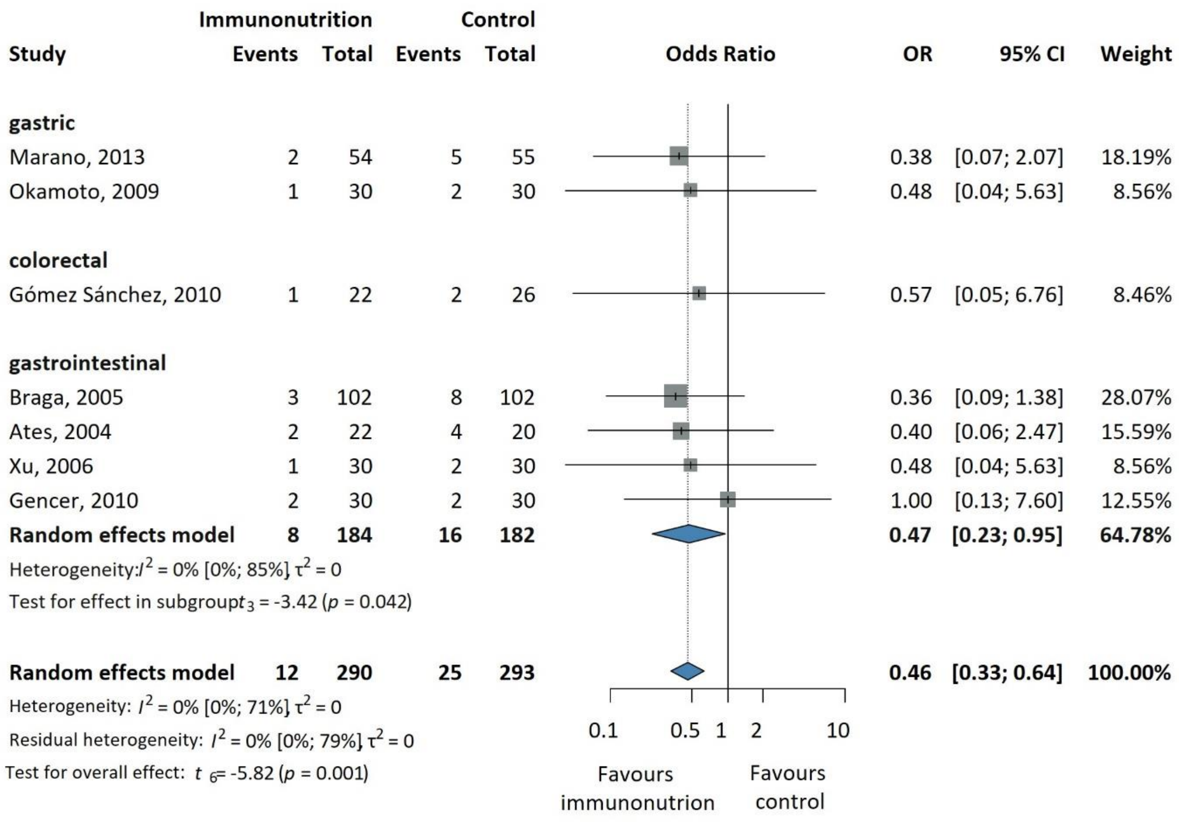

OR=odds ratio, 95% CI= 95% confidence interval

Figure 10: Pooled analysis of the effect of immunonutrition support (**arginine, nucleotides, omega-3 fatty acids**) in different administration time points on **respiratory infection** in gastrointestinal cancer

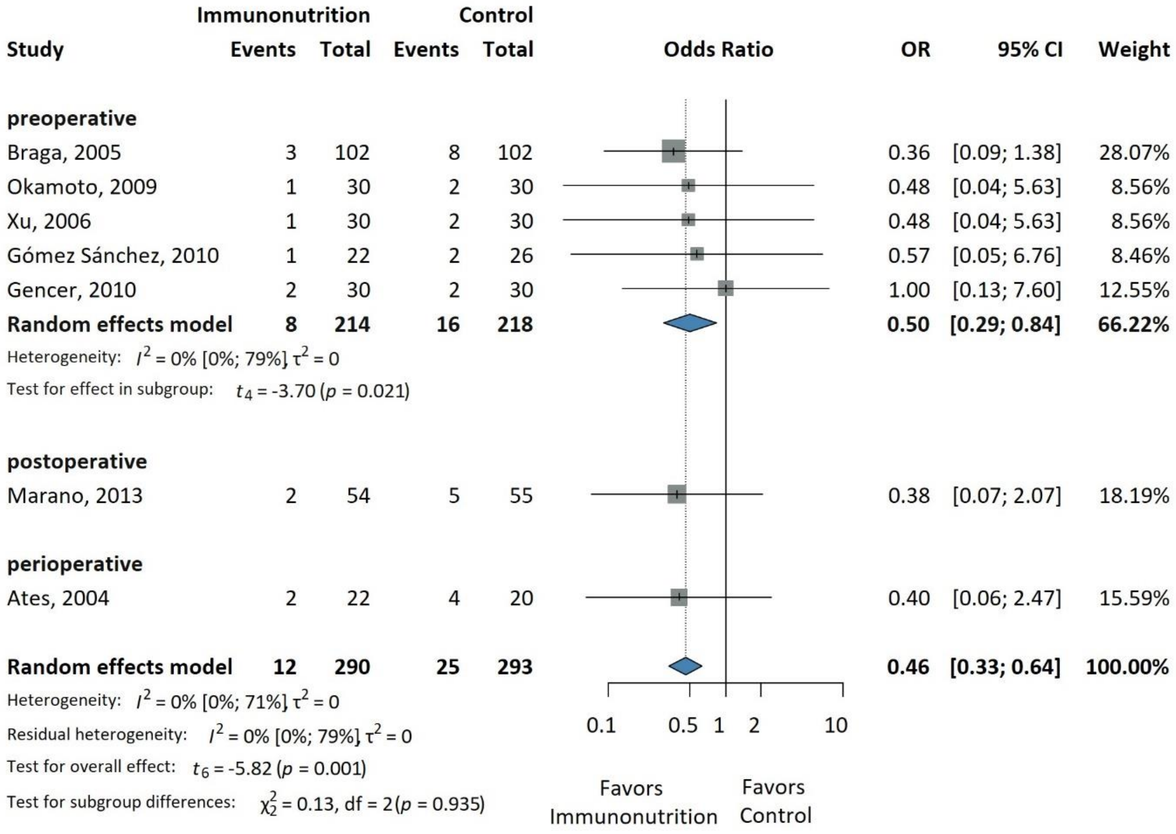

OR=odds ratio, 95% CI= 95% confidence interval

Figure 11: Pooled analysis of the effect of immunonutrition support (**arginine, nucleotides, omega-3 fatty acids**) on **urinary tract infection** in gastrointestinal cancer

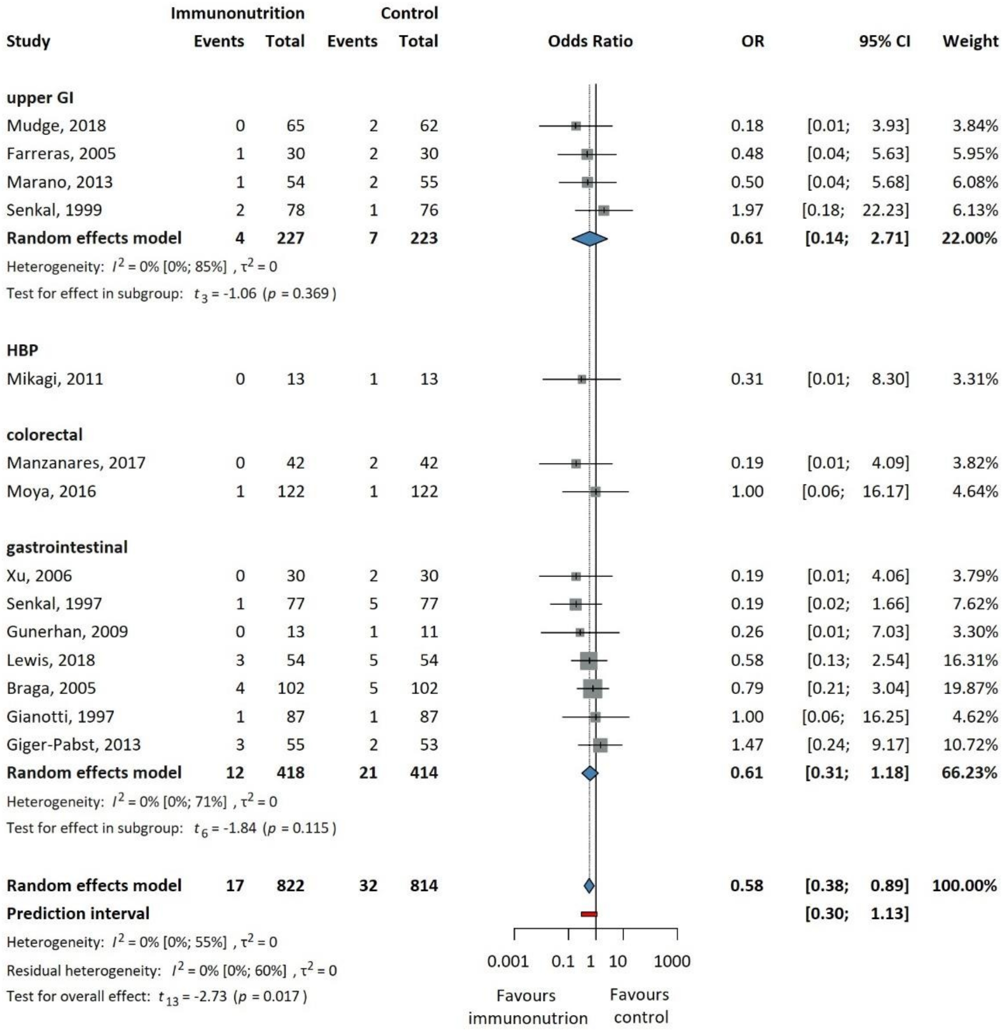

OR=odds ratio, 95% CI= 95% confidence interval, upper GI= upper gastrointestinal, HBP= hepatobiliary-pancreatic

Figure 12: Pooled analysis of the effect of **preoperative** immunonutrition support (**arginine, nucleotides, omega-3 fatty acids**) on **urinary tract infection** in gastrointestinal cancer

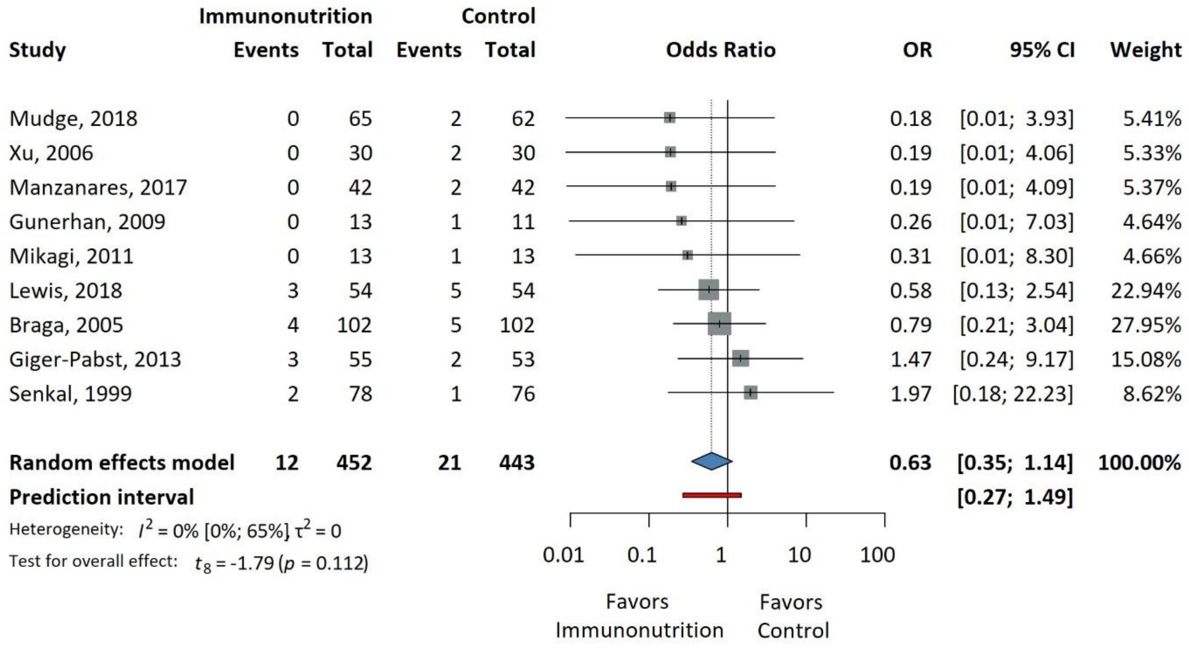

OR=odds ratio, 95% CI= 95% confidence interval

Figure 13: Pooled analysis of the effect of **postoperative** immunonutrition support (**arginine, nucleotides, omega-3 fatty acids**) on **urinary tract infection** in gastrointestinal cancer

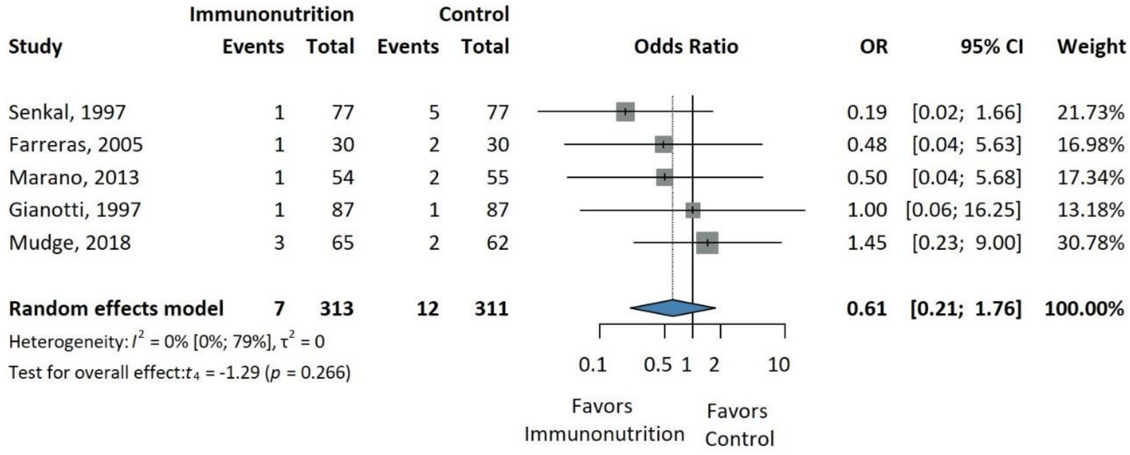

OR=odds ratio, 95% CI= 95% confidence interval

Figure 14: Pooled analysis of the effect of immunonutrition support (**arginine, nucleotides, omega-3 fatty acids**) on **wound infection** in gastrointestinal cancer

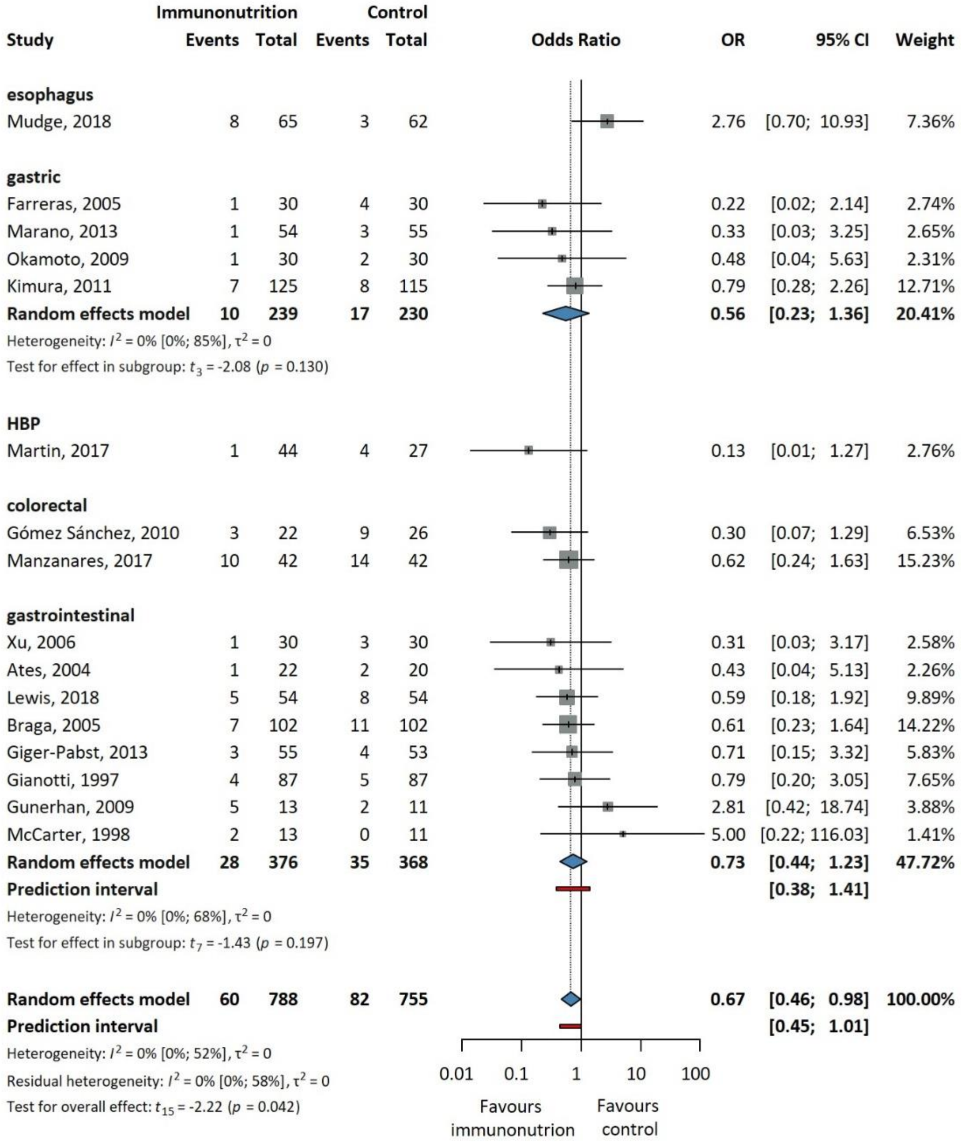

OR=odds ratio, 95% CI= 95% confidence interval, upper GI= upper gastrointestinal, HBP= hepatobiliary-pancreatic

Figure 15: Pooled analysis of the effect of **preoperative** immunonutrition support (**arginine, nucleotides, omega-3 fatty acids**) on **wound infection** in gastrointestinal cancer

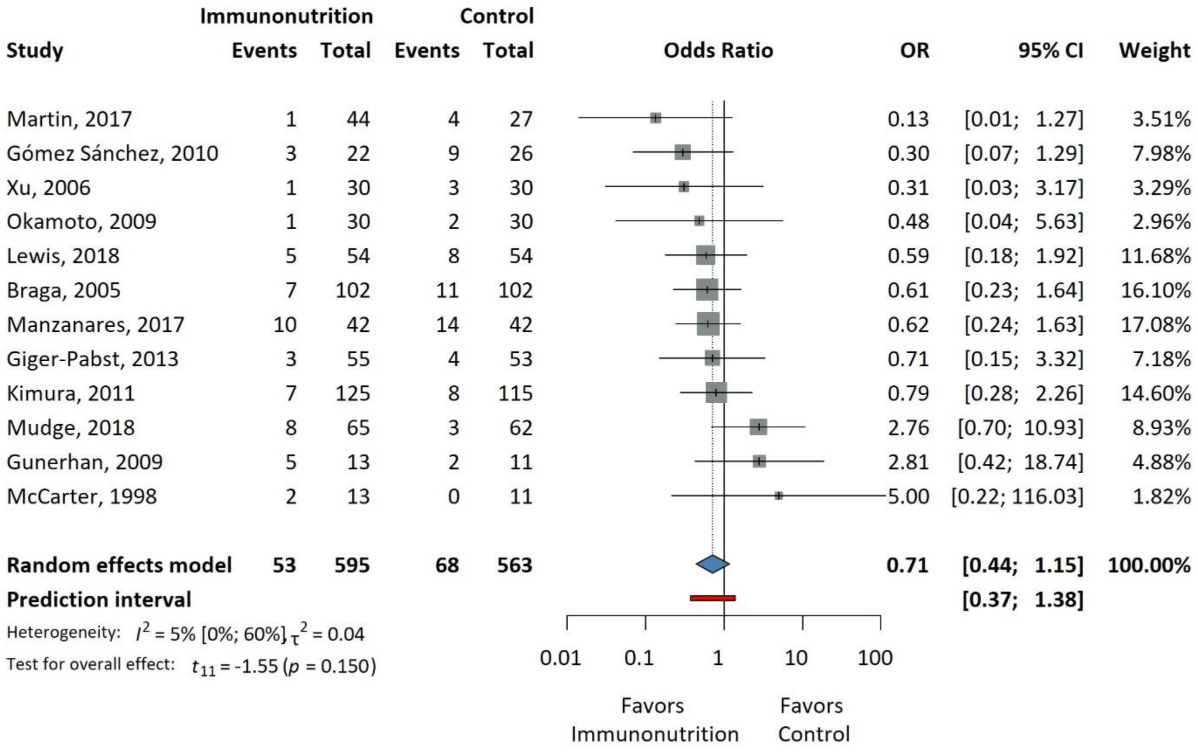

OR=odds ratio, 95% CI= 95% confidence interval

Figure 16: Pooled analysis of the effect of **postoperative** immunonutrition support (**arginine, nucleotides, omega-3 fatty acids**) on **wound infection** in gastrointestinal cancer

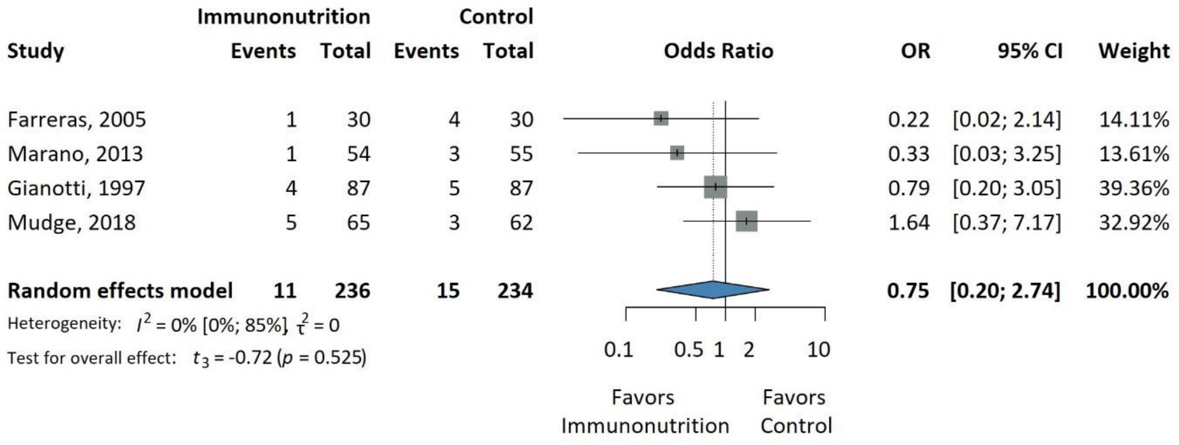

OR=odds ratio, 95% CI= 95% confidence interval

Figure 17: Pooled analysis of the effect of immunonutrition support (**arginine, nucleotides, omega-3 fatty acids**) on **sepsis** in gastrointestinal cancer

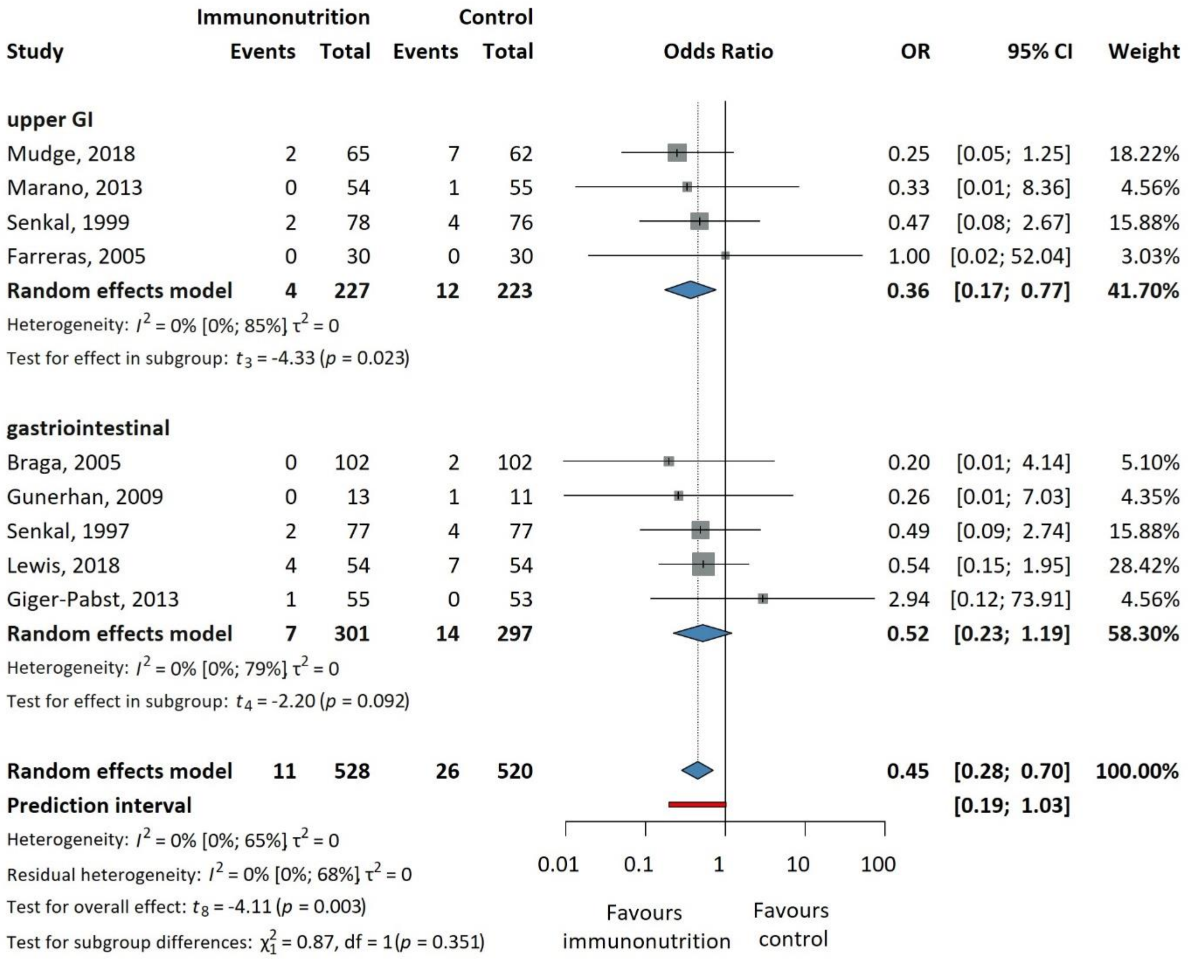

OR=odds ratio, 95% CI= 95% confidence interval, upper GI= upper gastrointestinal

Figure 18: Pooled analysis of the effect of **preoperative** immunonutrition support (**arginine, nucleotides, omega-3 fatty acids**) on **sepsis** in gastrointestinal cancer

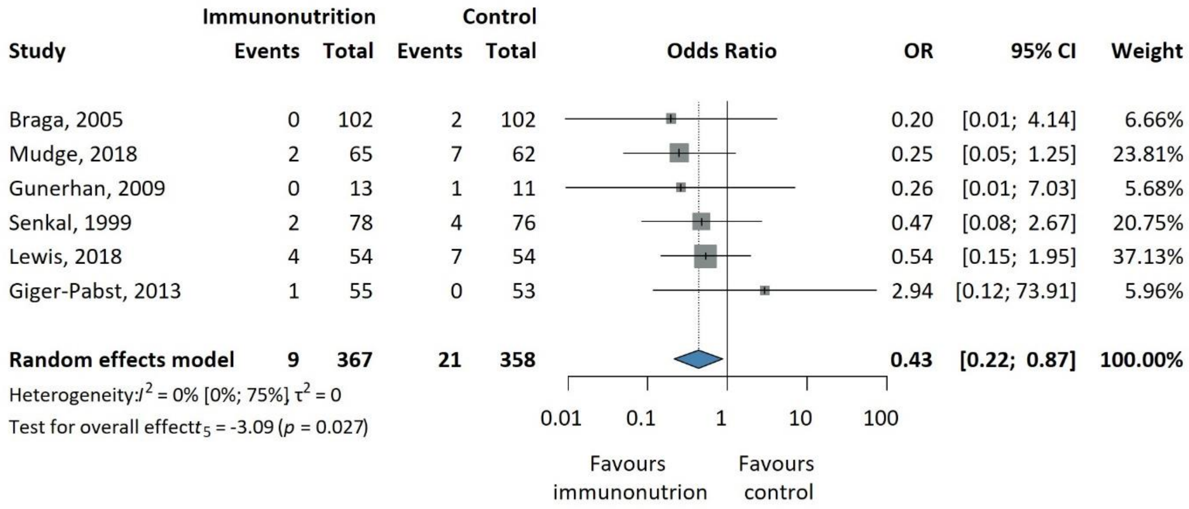

OR=odds ratio, 95% CI= 95% confidence interval

Figure 19: Pooled analysis of the effect of **postoperative** immunonutrition support (**arginine, nucleotides, omega-3 fatty acids**) on **sepsis** in gastrointestinal cancer

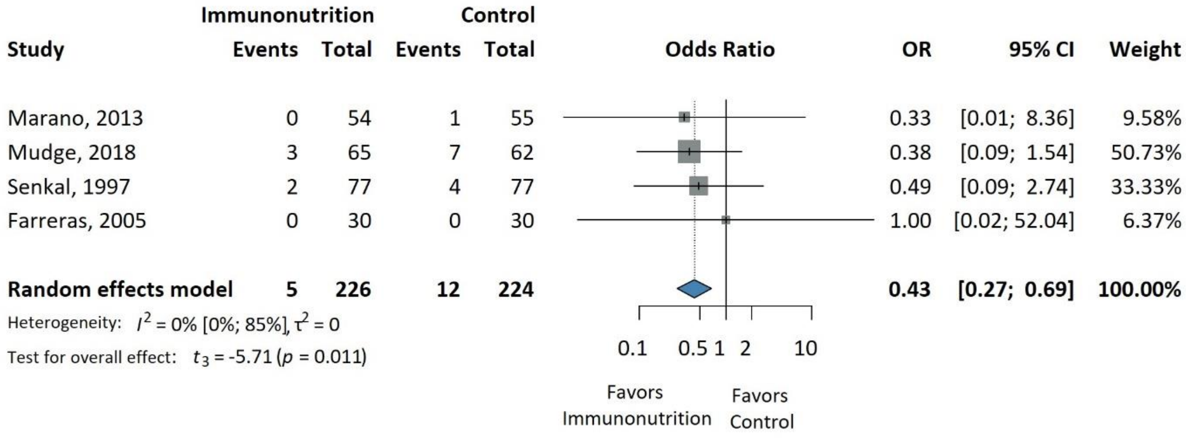

OR=odds ratio, 95% CI= 95% confidence interval

Figure 20: Pooled analysis of the effect of immunonutrition support (**arginine, nucleotides, omega-3 fatty acids**) on **surgical site infection** in gastrointestinal cancer

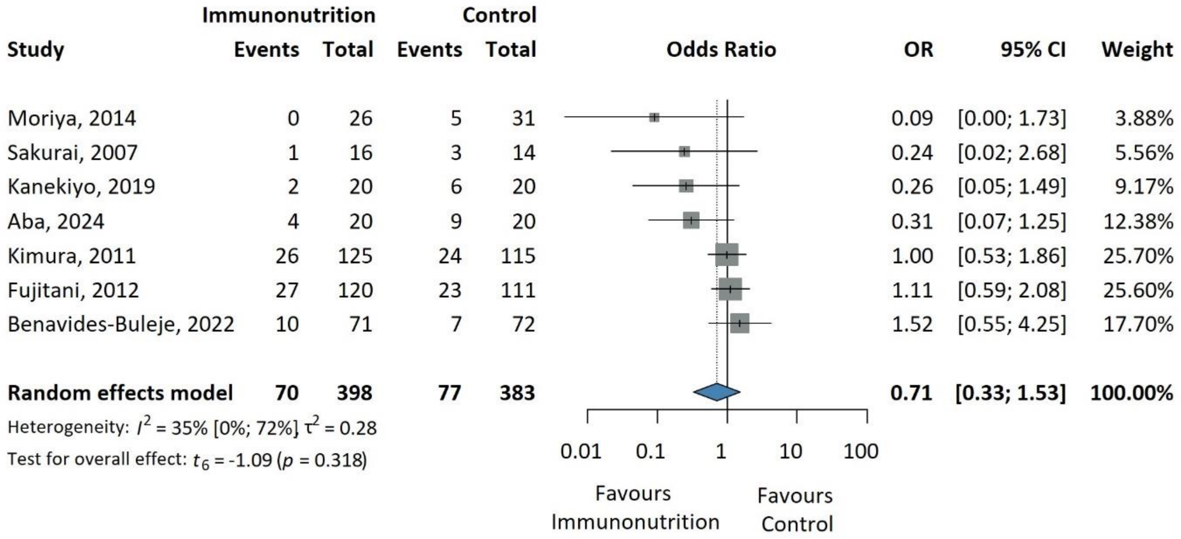

OR=odds ratio, 95% CI= 95% confidence interval

Figure 21: Pooled analysis of the effect of immunonutrition support (**arginine, nucleotides, omega-3 fatty acids**) on **non-infectious complications** in gastrointestinal cancer

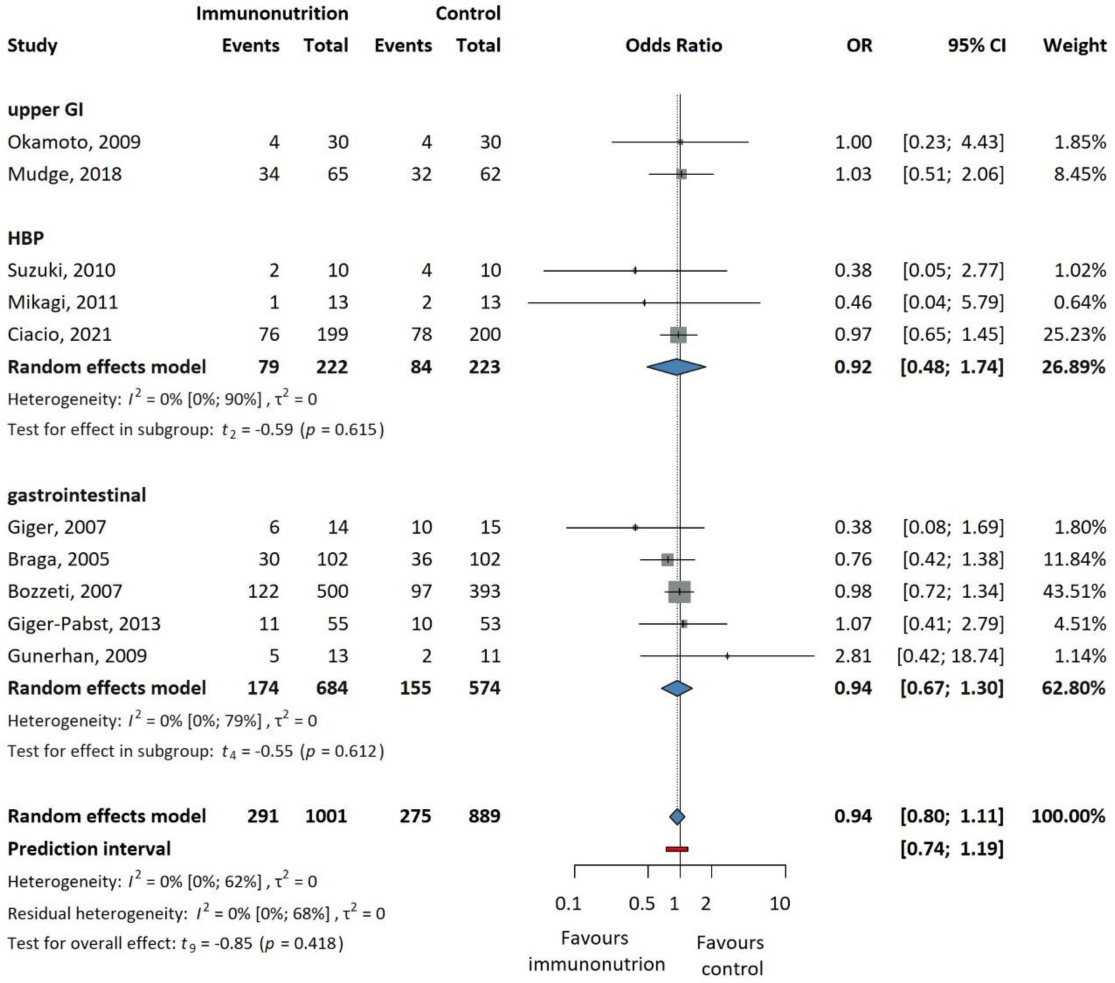

OR=odds ratio, 95% CI= 95% confidence interval, upper GI= upper gastrointestinal, HBP= hepatobiliary-pancreatic

Figure 22: Pooled analysis of the effect of **preoperative** immunonutrition support (**arginine, nucleotides, omega-3 fatty acids**) on **non-infectious complications** in gastrointestinal cancer

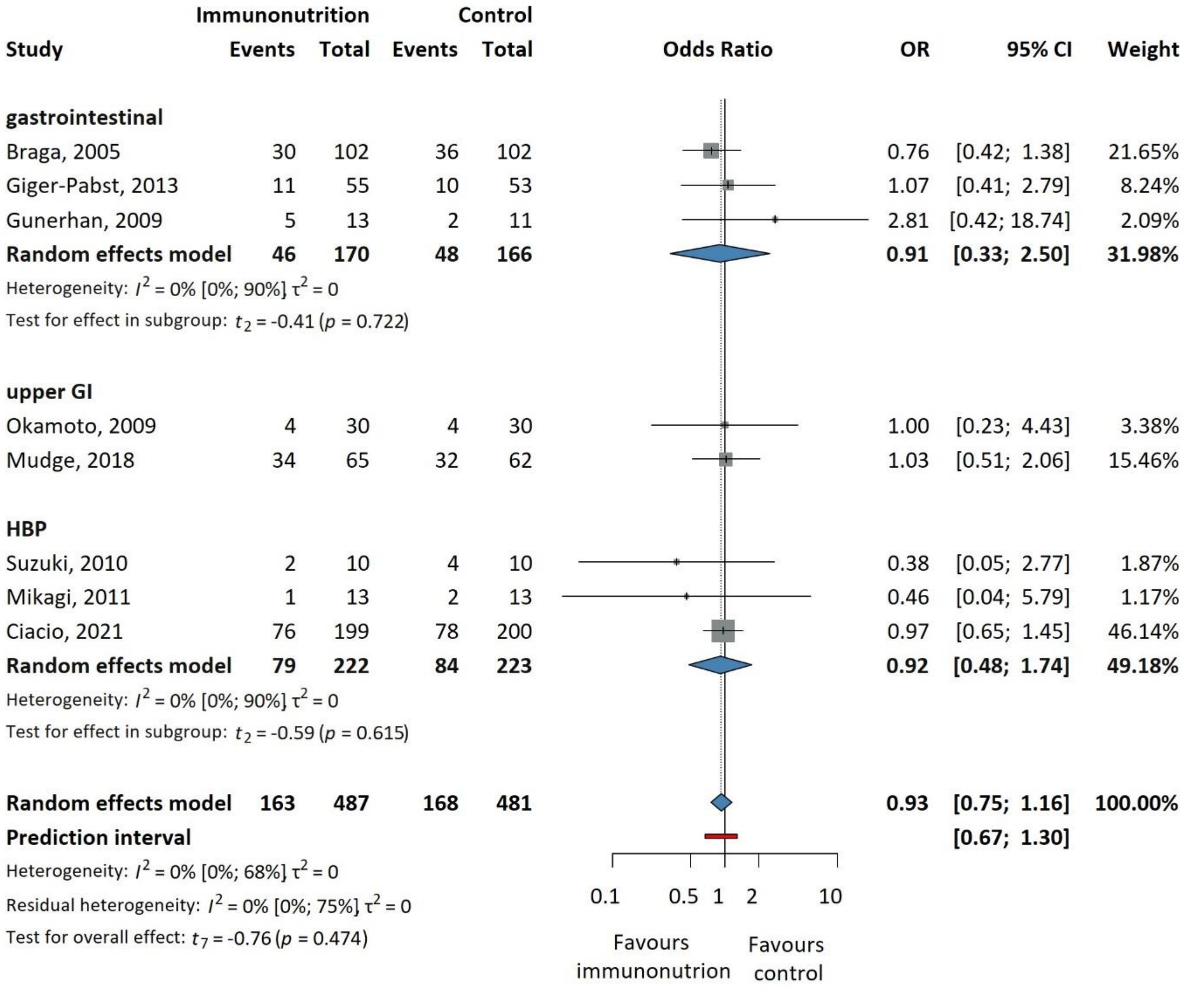

OR=odds ratio, 95% CI= 95% confidence interval, upper GI= upper gastrointestinal, HBP= hepatobiliary-pancreatic

Figure 23: Pooled analysis of the effect of **postoperative** immunonutrition support (**arginine, nucleotides, omega-3 fatty acids**) on **non-infectious complications** in gastrointestinal cancer

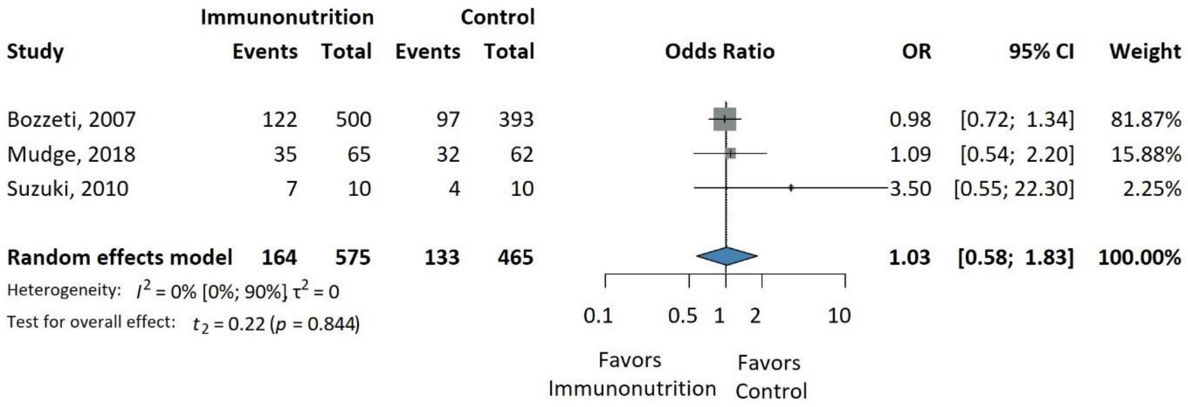

OR=odds ratio, 95% CI= 95% confidence interval

Figure 24: Pooled analysis of the effect of immunonutrition support (**omega-3 fatty acids**) on **urinary tract infection** in gastrointestinal cancer

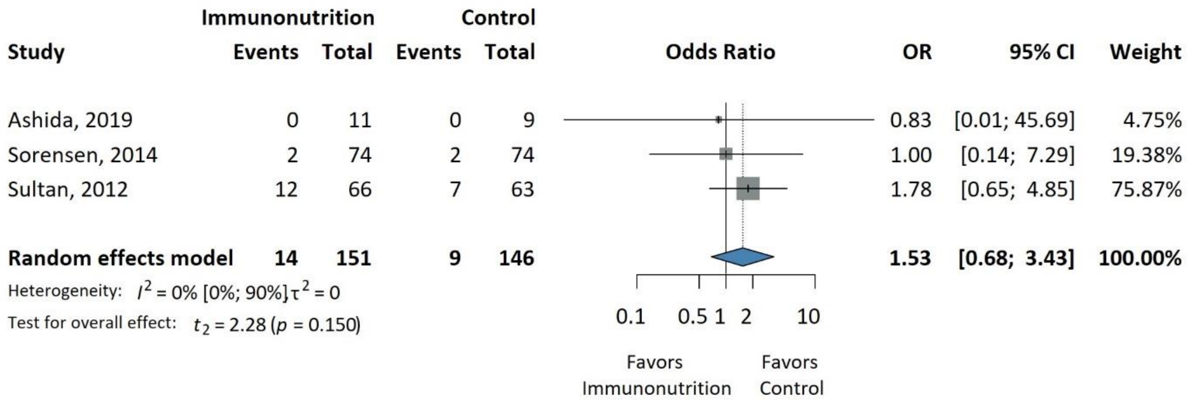

OR=odds ratio, 95% CI= 95% confidence interval

Figure 25: Pooled analysis of the effect of immunonutrition support (**omega-3 fatty acids**) on **sepsis** in gastrointestinal cancer

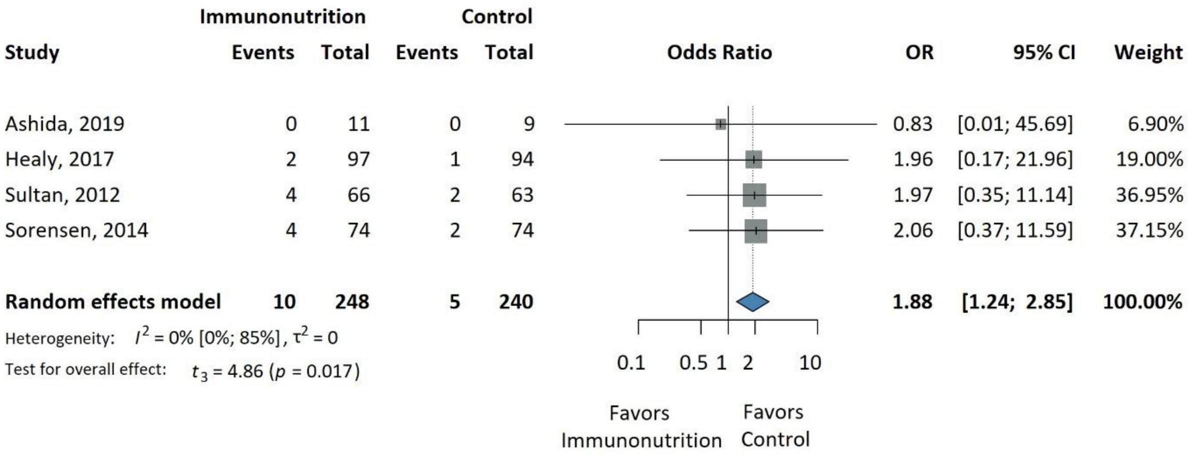

OR=odds ratio, 95% CI= 95% confidence interval

Figure 26: Pooled analysis of the effect of **preoperative** immunonutrition support (**arginine, nucleotides, omega-3 fatty acids**) on **anastomotic leakage** in gastrointestinal cancer

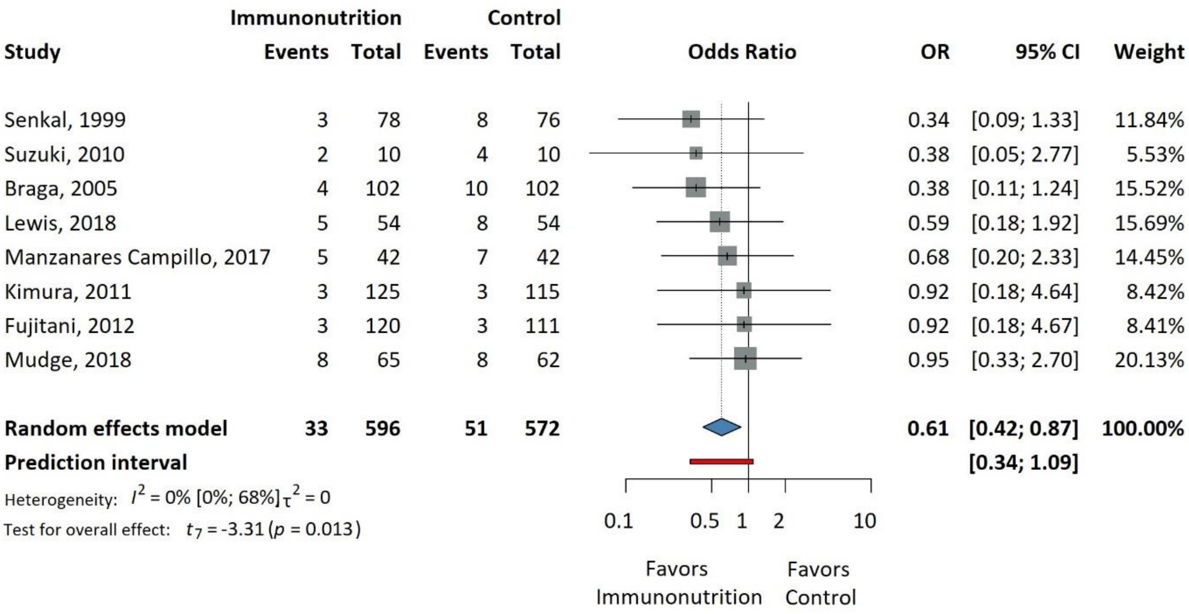

OR=odds ratio, 95% CI= 95% confidence interval

Figure 27: Pooled analysis of the effect of **postoperative** immunonutrition support (**arginine, nucleotides, omega-3 fatty acids**) on **anastomotic leakage** in gastrointestinal cancer

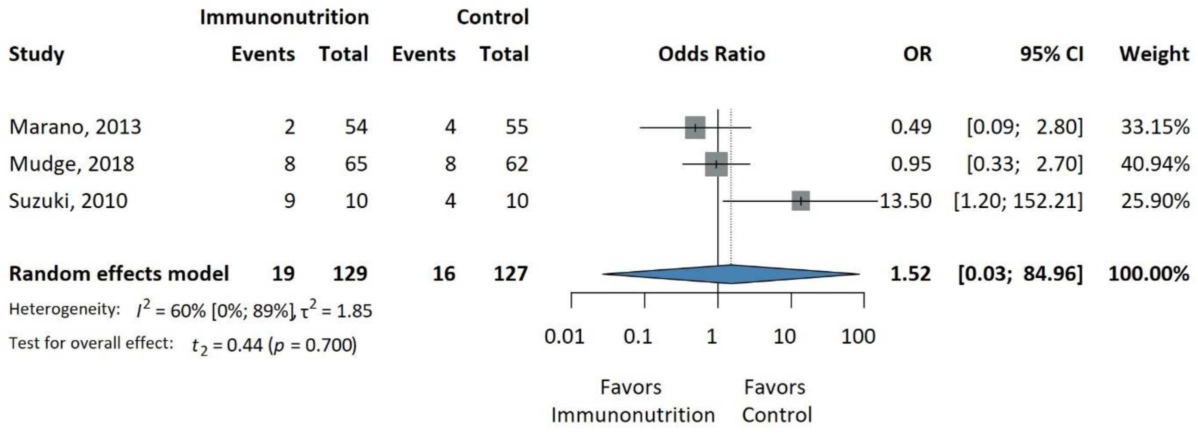

OR=odds ratio, 95% CI= 95% confidence interval

Figure 28: Pooled analysis of the effect of immunonutrition support (**omega-3 fatty acids**) on **anastomotic leak** in gastrointestinal cancer

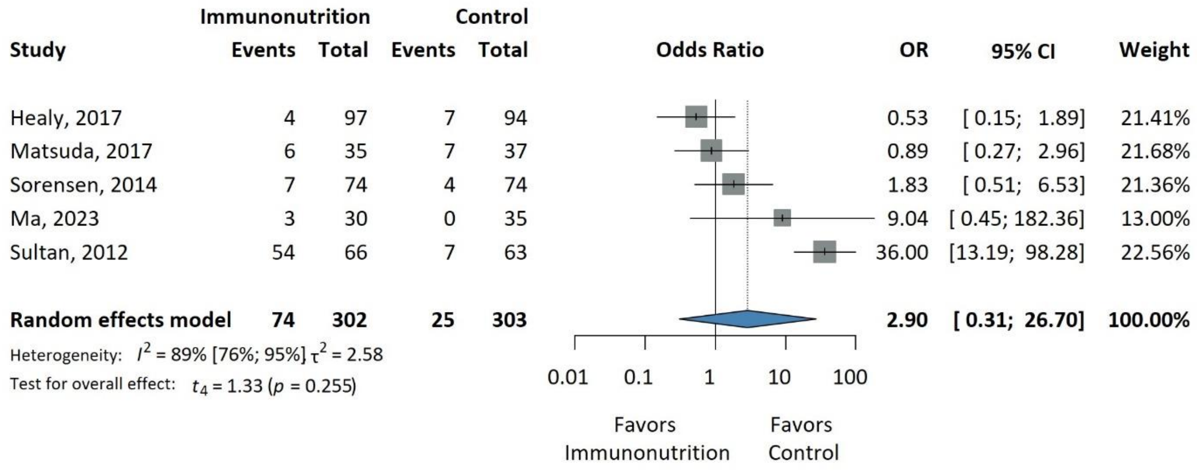

OR=odds ratio, 95% CI= 95% confidence interval

Figure 29: Pooled analysis of the effect of immunonutrition support (**arginine, nucleotides, omega-3 fatty acids**) at different time points on the **length of hospital stay** in gastrointestinal cancer

Study	Immunonutrition N	Immunonutrition Mean	Immunonutrition SD	Control N	Control Mean	Control SD	MD	95% CI	Weight
**preoperative**									
Martin, 2017 *	44	10.56	8.42	27	18.54	23.36	-7.98	[-17.14; 1.17]	2.33%
Senkal, 1999	78	22.20	4.10	76	25.80	3.80	-3.60	[-4.85; -2.35]	9.08%
Aba, 2024	20	12.89	3.30	20	16.47	6.83	-3.58	[-6.90; -0.26]	6.80%
Xu, 2006	30	9.00	2.20	30	12.00	3.70	-3.00	[-4.54; -1.46]	8.82%
Braga, 2005	102	11.60	4.70	102	14.00	7.70	-2.40	[-4.15; -0.65]	8.62%
Fujitani, 2012 *	120	38.39	57.01	111	39.51	58.56	-1.12	[-16.04; 13.81]	1.03%
Manzanares Campillo, 2017	42	12.70	8.30	42	13.10	10.80	-0.40	[-4.52; 3.72]	5.88%
Giger-Pabst, 2013	55	12.00	4.90	53	11.60	5.30	0.40	[-1.53; 2.33]	8.43%
Ciacio, 2021	199	12.00	11.00	200	11.00	10.00	1.00	[-1.06; 3.06]	8.29%
Mudge, 2018	65	14.00	6.05	62	13.00	3.94	1.00	[-0.77; 2.77]	8.60%
Gunerhan, 2009	13	16.54	14.83	11	14.22	9.12	2.32	[-7.38; 12.02]	2.13%
**Random effects model**	**768**			**734**			**-1.42**	**[-2.95; 0.10]**	**70.01%**
**Prediction interval**								**[-5.65; 2.80]**	
Heterogeneity: I^2 = 73% [50%; 85%] , τ^2 = 3.08									
Test for effect in subgroup: t_{10} = -2.08 (p = 0.065)									
**postoperative**									
Marano, 2013	54	12.70	2.30	55	15.90	3.40	-3.20	[-4.29; -2.11]	9.20%
Gianotti, 1997	87	16.10	6.20	87	19.20	7.90	-3.10	[-5.21; -0.99]	8.23%
**perioperative**									
Giger, 2007	14	13.70	2.30	15	23.10	3.60	-9.40	[-11.58; -7.22]	8.15%
Kanekiyo, 2019 *	20	27.28	4.76	20	30.52	11.90	-3.24	[-8.86; 2.38]	4.41%
**Random effects model**	**943**			**911**			**-2.47**	**[-4.13; -0.80]**	**100.00%**
**Prediction interval**								**[-8.40; 3.46]**	

Heterogeneity: I^2 = 83% [73%; 89%] , τ^2 = 6.98
Residual heterogeneity: I^2 = 70% [48%; 83%] , τ^2 = 3.08
Test for overall effect: t_{14} = -3.18 (p = 0.007)

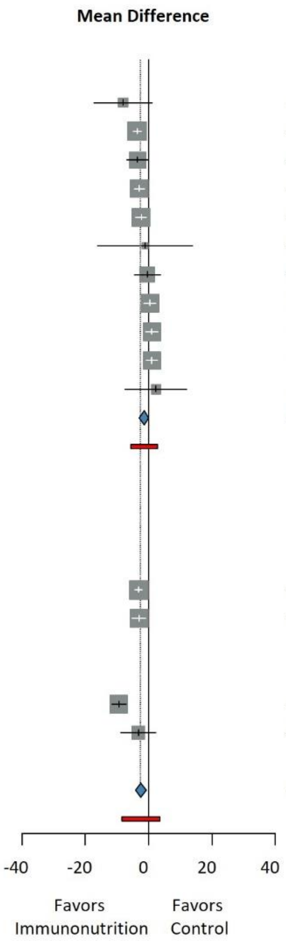

Means and/or standard deviations of articles marked with * were estimated from medians, quartiles or minimum/maximum values.

SD= standard deviation, MD= mean difference, CI= 95% confidence interval, N= number of patients

Supplementary Figures 30-38: Individual Forest plots for subgroup analysis based on cancer stage

Figure 30: Subgroup analysis based on cancer stage of the effect of immunonutrition support (**arginine, nucleotides, omega-3 fatty acids**) on **anastomotic leakage** in gastrointestinal cancer

Study	Immunonutrition Events	Immunonutrition Total	Control Events	Control Total	OR	95% CI	Weight
**I-III stage**							
Marano, 2013	2	54	4	55	0.49	[0.09; 2.80]	5.00%
Kimura, 2011	3	125	3	115	0.92	[0.18; 4.64]	5.77%
Fujitani, 2012	3	120	3	111	0.92	[0.18; 4.67]	5.77%
**Random effects model**	**8**	**299**	**10**	**281**	**0.76**	**[0.32; 1.83]**	**16.55%**
Heterogeneity: I^2 = 0% [0%; 90%] , τ = 0							
Test for effect in subgroup: t_2 = -1.34 (p = 0.313)							
**I-IV stage**							
Kanekiyo, 2019	0	20	1	20	0.32	[0.01; 8.26]	1.43%
Suzuki, 2010	2	10	4	10	0.38	[0.05; 2.77]	3.79%
Moya, 2016	6	122	10	122	0.58	[0.20; 1.65]	13.89%
**Random effects model**	**8**	**152**	**15**	**152**	**0.51**	**[0.26; 0.99]**	**19.11%**
Heterogeneity: I^2 = 0% [0%; 90%] , τ = 0							
Test for effect in subgroup: t_2 = -4.40 (p = 0.048)							
**NA**							
Senkal, 1999	3	78	8	76	0.34	[0.09; 1.33]	8.12%
Braga, 2002	3	50	6	50	0.47	[0.11; 1.99]	7.26%
Lewis, 2018	5	54	8	54	0.59	[0.18; 1.92]	10.75%
Senkal, 1997	7	77	10	77	0.67	[0.24; 1.86]	14.51%
Manzanares Campillo, 2017	5	42	7	42	0.68	[0.20; 2.33]	9.91%
Mudge, 2018	8	65	8	62	0.95	[0.33; 2.70]	13.80%
**Random effects model**	**31**	**366**	**47**	**361**	**0.62**	**[0.44; 0.89]**	**64.35%**
Heterogeneity: I^2 = 0% [0%; 75%] , τ = 0							
Test for effect in subgroup: t_5 = -3.41 (p = 0.019)							
**Random effects model**	**47**	**817**	**72**	**794**	**0.62**	**[0.50; 0.76]**	**100.00%**

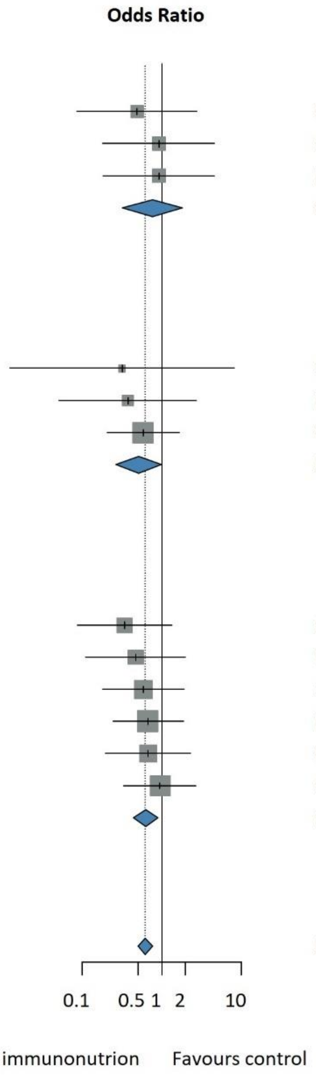

Heterogeneity: I^2 = 0% [0%; 58%] , τ = 0 [<0.0000; <0.0000]
Residual heterogeneity: I^2 = 0% [0%; 62%] , τ = 0
Test for overall effect: t_{11} = -5.04 (p < 0.001)
Test for subgroup differences: χ^2_2= 2.59, df = 2 (p = 0.274)

OR=odds ratio, 95% CI= 95% confidence interval, NA= not available

Figure 31: Subgroup analysis based on cancer stage of the effect of immunonutrition support (**arginine, nucleotides, omega-3 fatty acids**) on **infectious complications** in gastrointestinal cancer

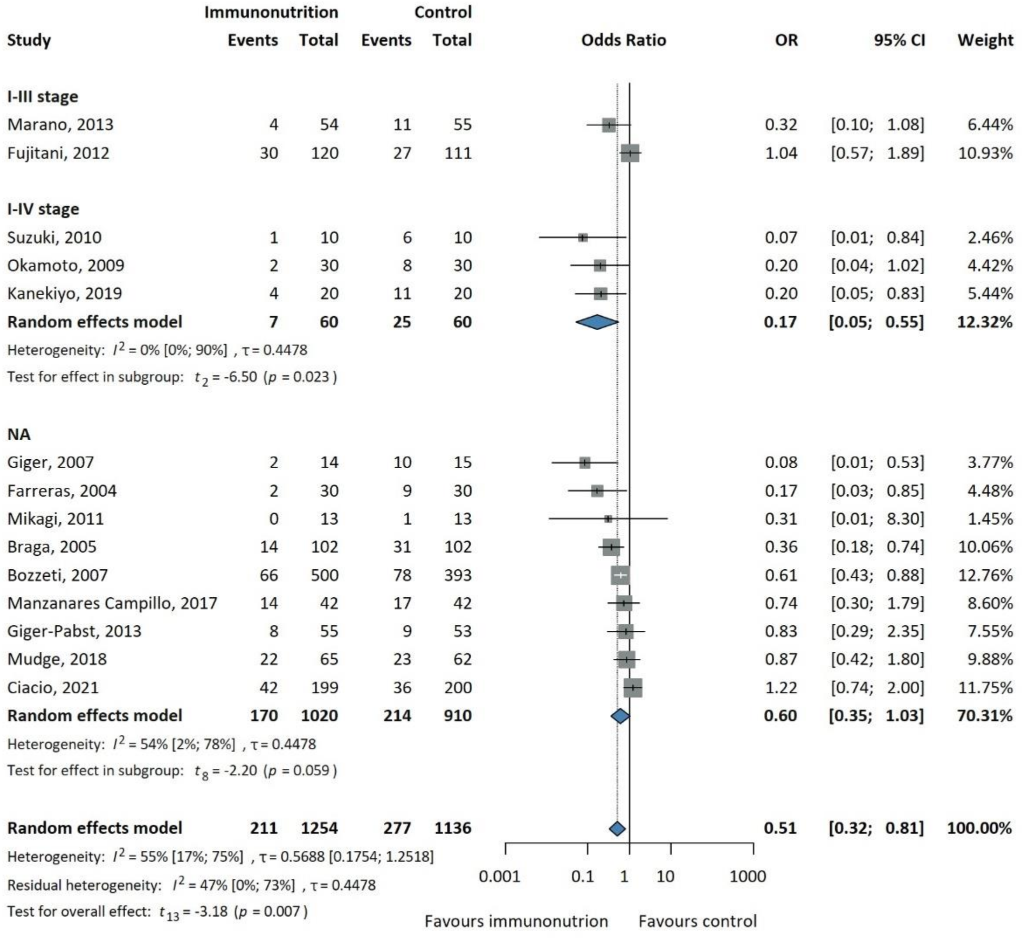

OR=odds ratio, 95% CI= 95% confidence interval, NA= not available

Figure 32: Subgroup analysis based on cancer stage of the effect of immunonutrition support (**arginine, nucleotides, omega-3 fatty acids**) at different time points on the **length of hospital stay** in gastrointestinal cancer

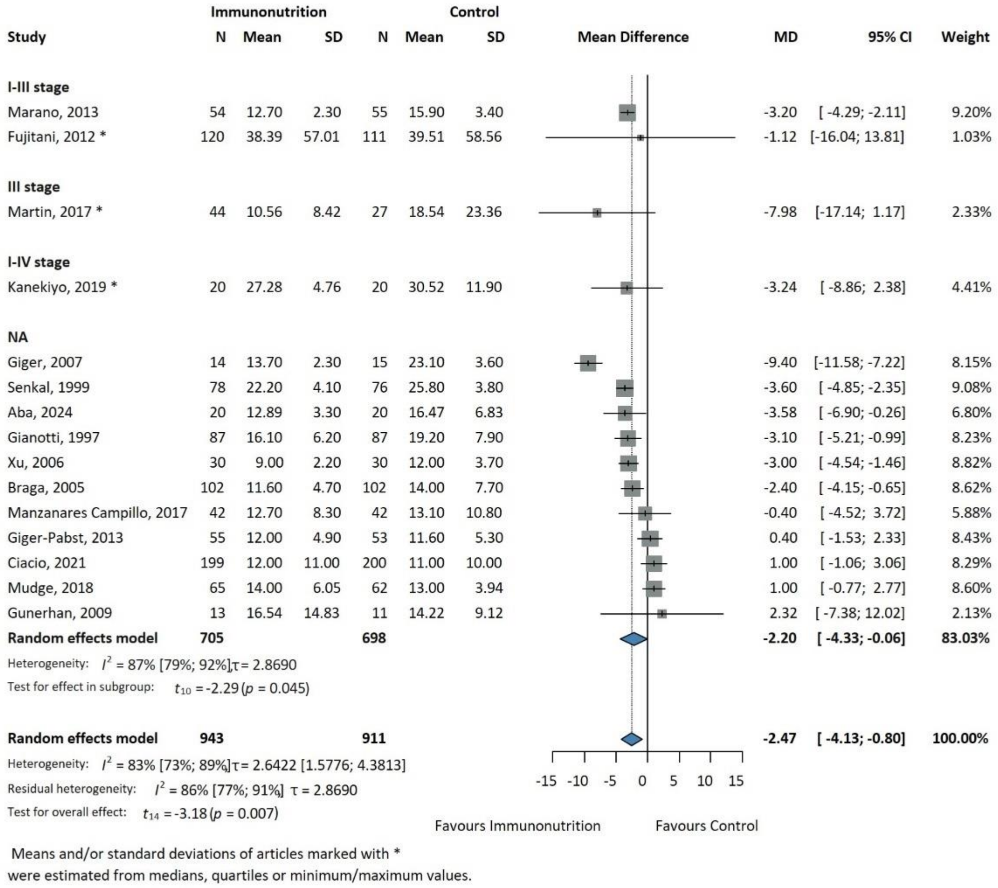

SD= standard deviation, MD= mean difference, CI= 95% confidence interval, N= number of patients, NA= not available

Figure 33: Subgroup analysis based on cancer stage of the effect of **postoperative** immunonutrition support (**arginine, nucleotides, omega-3 fatty acids**) on **anastomotic leakage** in gastrointestinal cancer

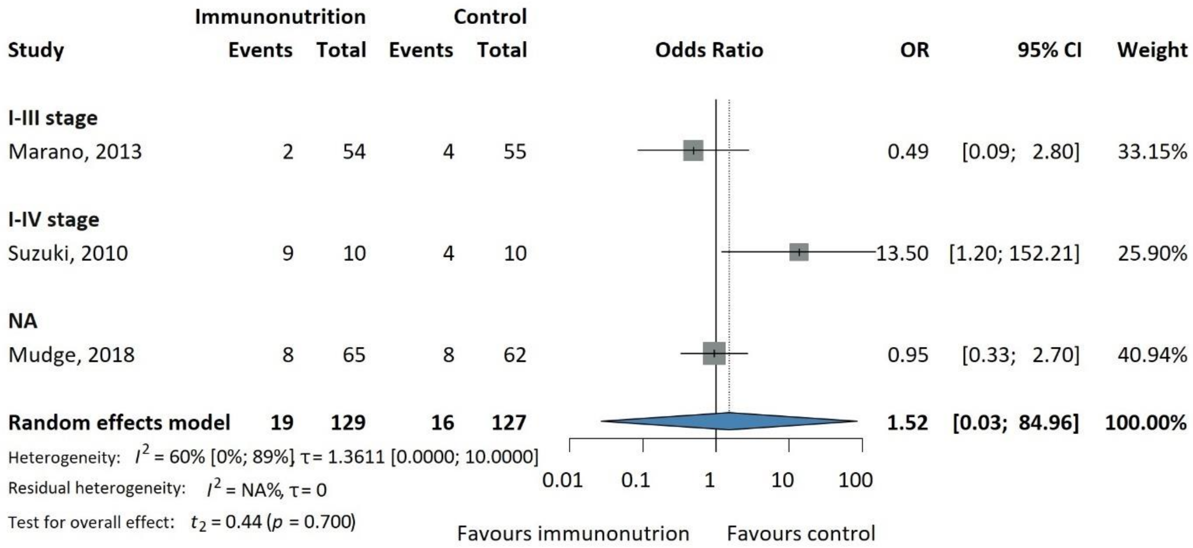

OR=odds ratio, 95% CI= 95% confidence interval, NA= not available

Figure 34: Subgroup analysis based on cancer stage of the effect of immunonutrition support (**omega-3 fatty acids**) on **anastomotic leak** in gastrointestinal cancer

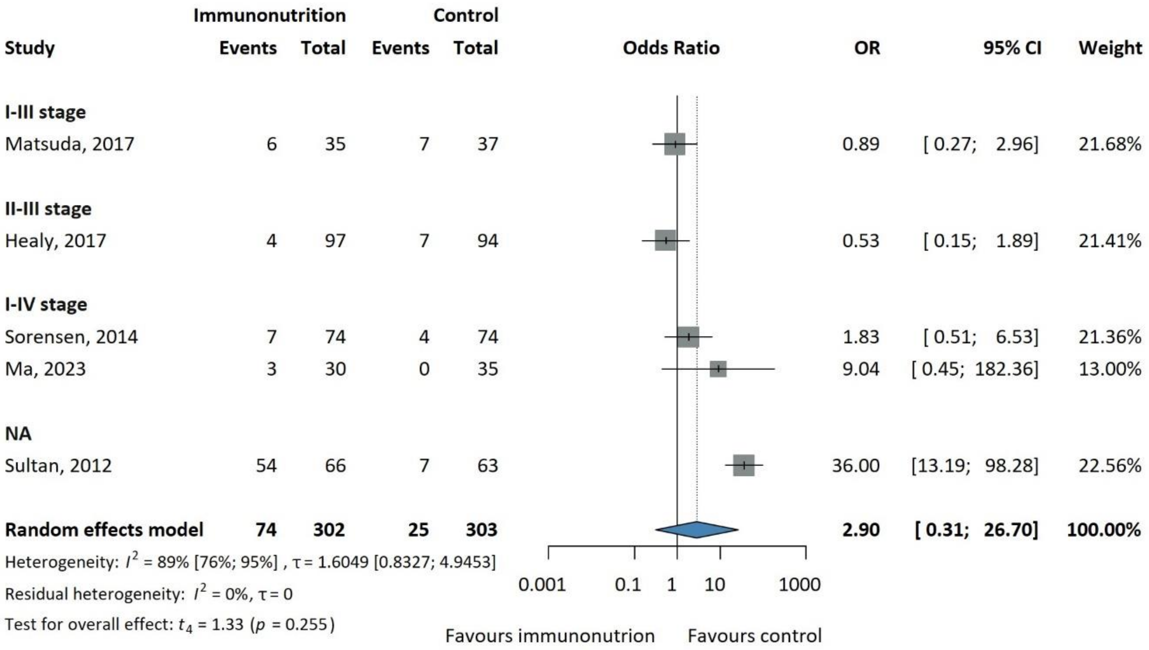

OR=odds ratio, 95% CI= 95% confidence interval, NA= not available

Figure 35: Subgroup analysis based on cancer stage of the effect of immunonutrition support (**arginine, nucleotides, omega-3 fatty acids**) on **in-hospital mortality** in gastrointestinal cancer

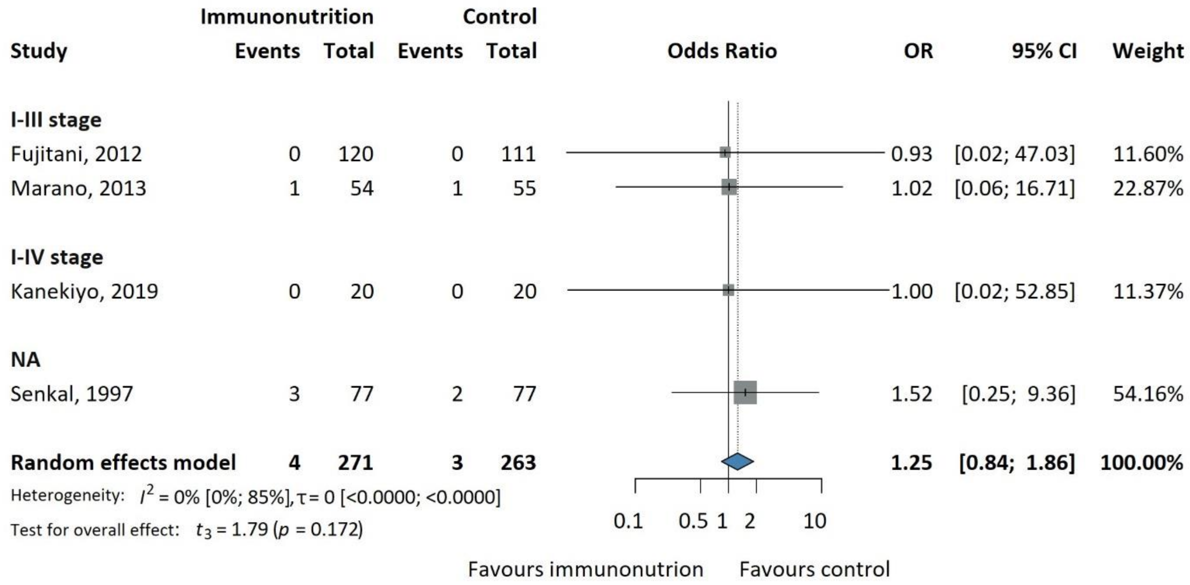

OR=odds ratio, 95% CI= 95% confidence interval, NA= not available

Figure 36: Subgroup analysis based on cancer stage of the effect of immunonutrition support (**mixed types**) on **3-year mortality** in gastrointestinal cancer

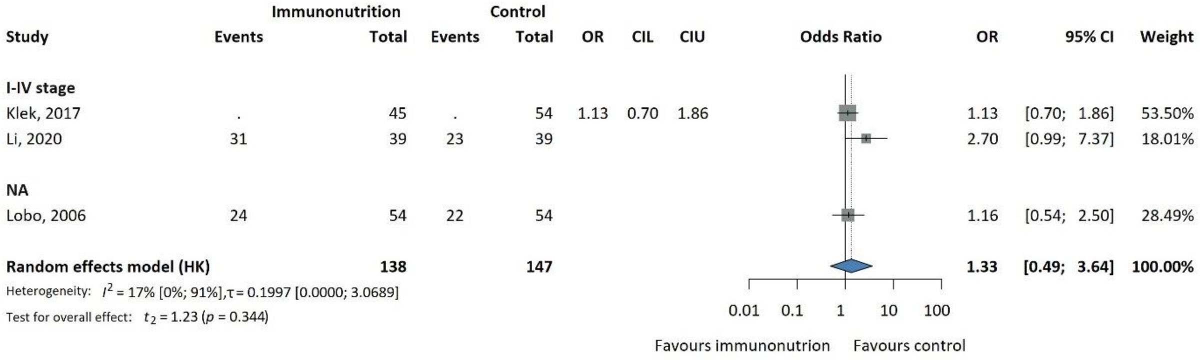

OR=odds ratio, 95% CI= 95% confidence interval, NA= not available

Figure 37: Subgroup analysis based on cancer stage of the effect of immunonutrition support (**mixed types**) on **5-year mortality** in gastrointestinal cancer

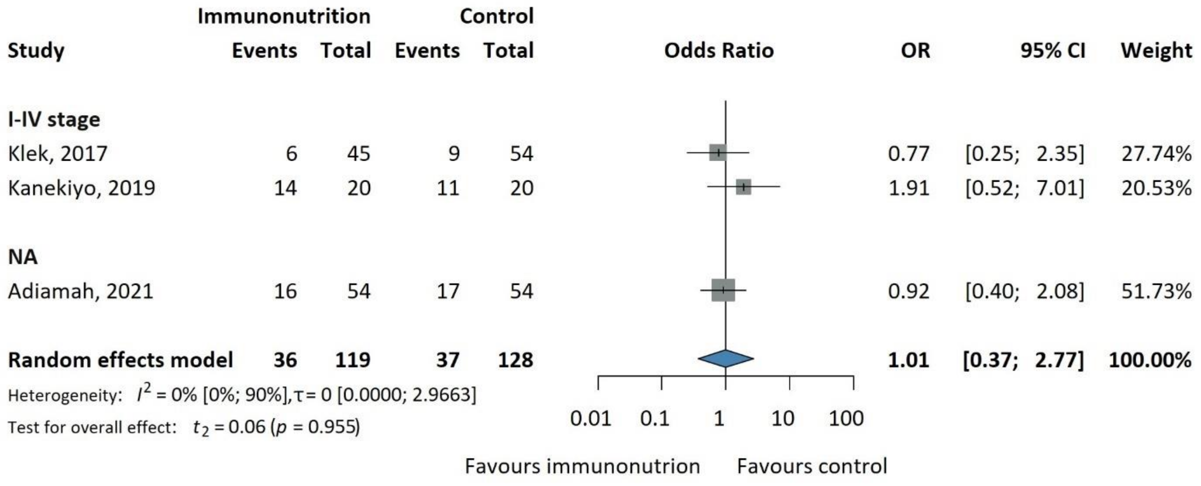

OR=odds ratio, 95% CI= 95% confidence interval, NA= not available

Figure 38: Subgroup analysis based on cancer stage of the effect of immunonutrition support (**arginine, nucleotides, omega-3 fatty acids**) on **surgical site infection** in gastrointestinal cancer

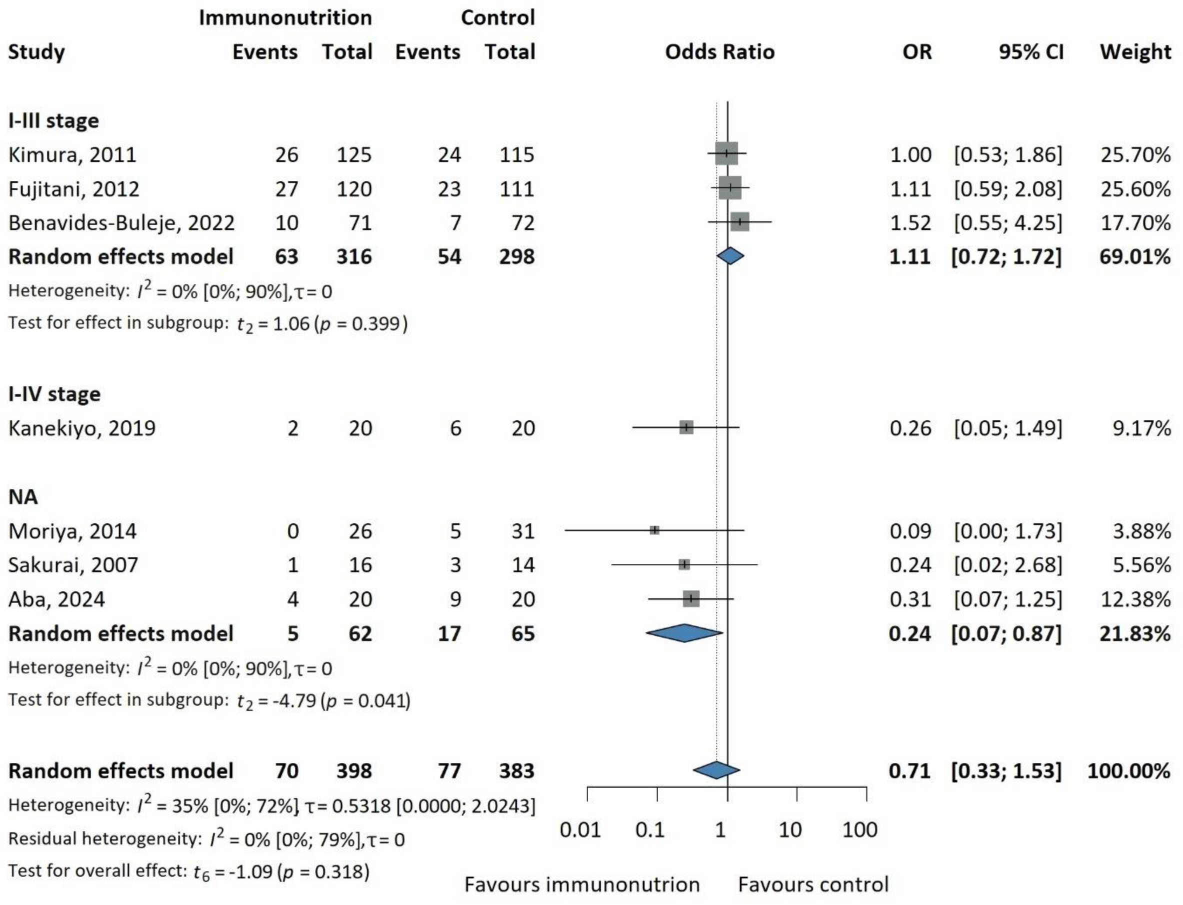

OR=odds ratio, 95% CI= 95% confidence interval, NA= not available

Supplementary Figures 39- 45: Funnel plots for publication bias

Figure 39. Funnel plot: Analysis of the effect of perioperative immunonutrition support (arginine, nucleotides, omega-3 fatty acids) on anastomotic leakage in gastrointestinal cancer patients

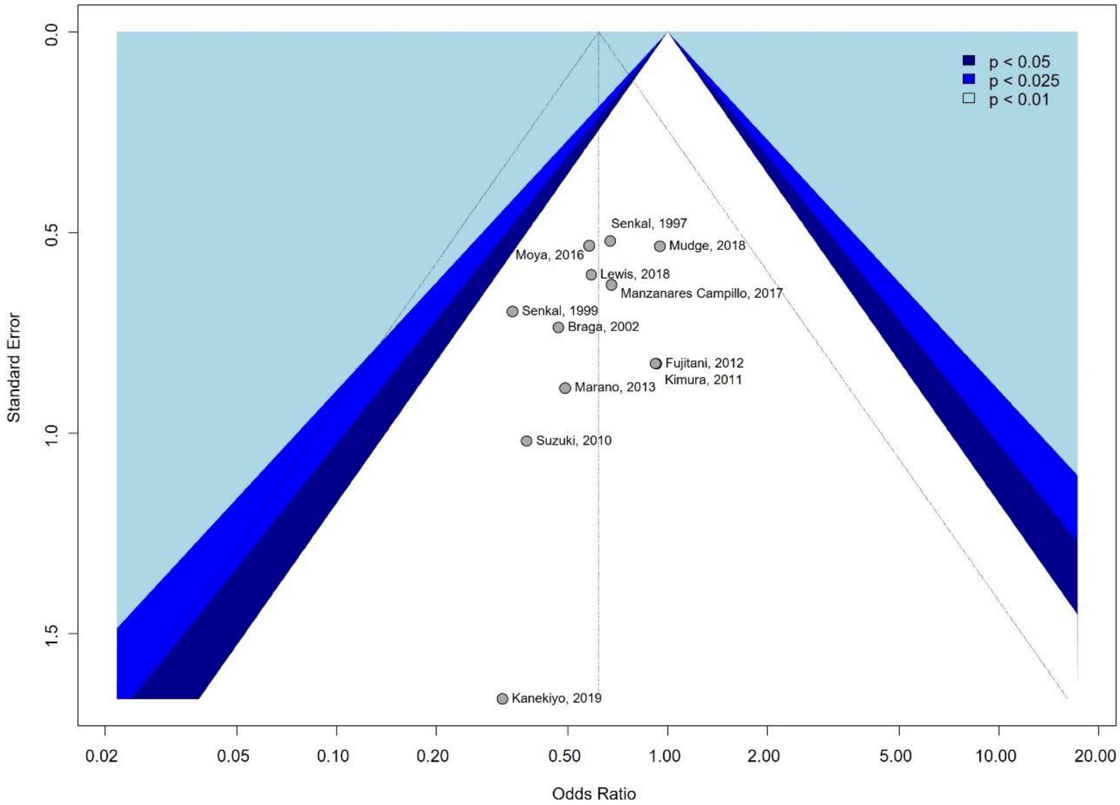

Egger test: t = -1.24, df = 10, p-value = 0.2444

Figure 40. Funnel plot: Analysis of the effect of perioperative immunonutrition support (arginine, nucleotides, omega-3 fatty acids) on infection complications in gastrointestinal cancer patients

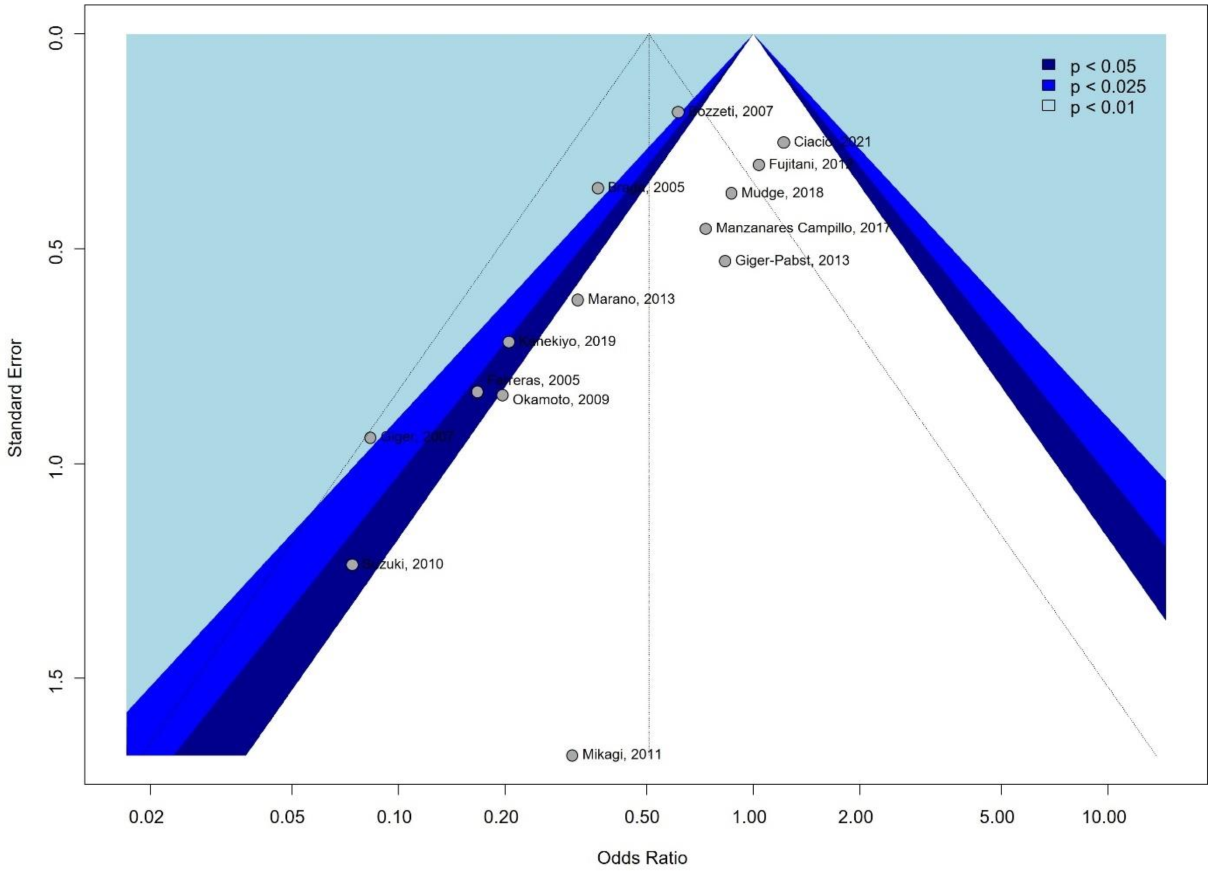

Egger test: t = -3.61, df = 12, p-value = 0.0036

Figure 41. Funnel plot: Analysis of the effect of perioperative immunonutrition support (arginine, nucleotides, omega-3 fatty acids) on urinary tract infection in gastrointestinal cancer patients

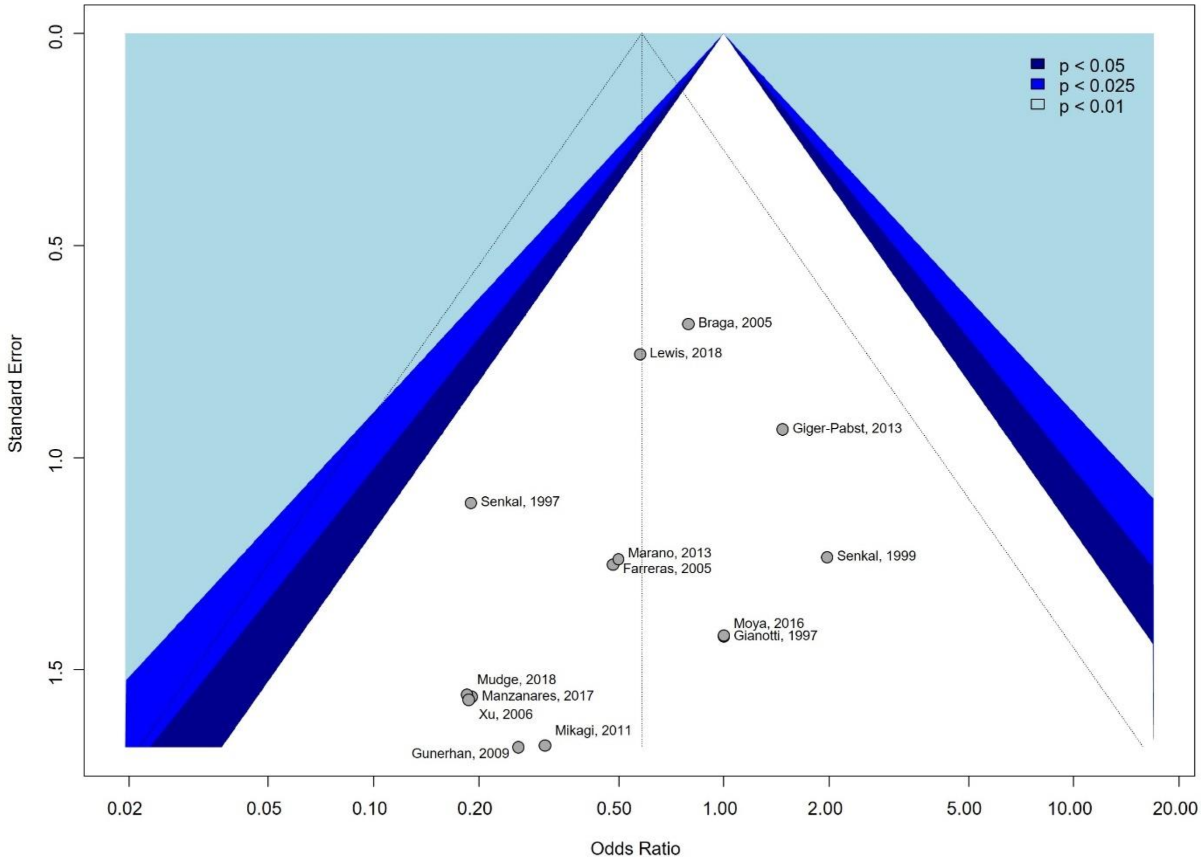

Egger test: t = -1.12, df = 12, p-value = 0.2832

Figure 42. Funnel plot: Analysis of the effect of perioperative immunonutrition support (arginine, nucleotides, omega-3 fatty acids) on wound infection in gastrointestinal cancer patients

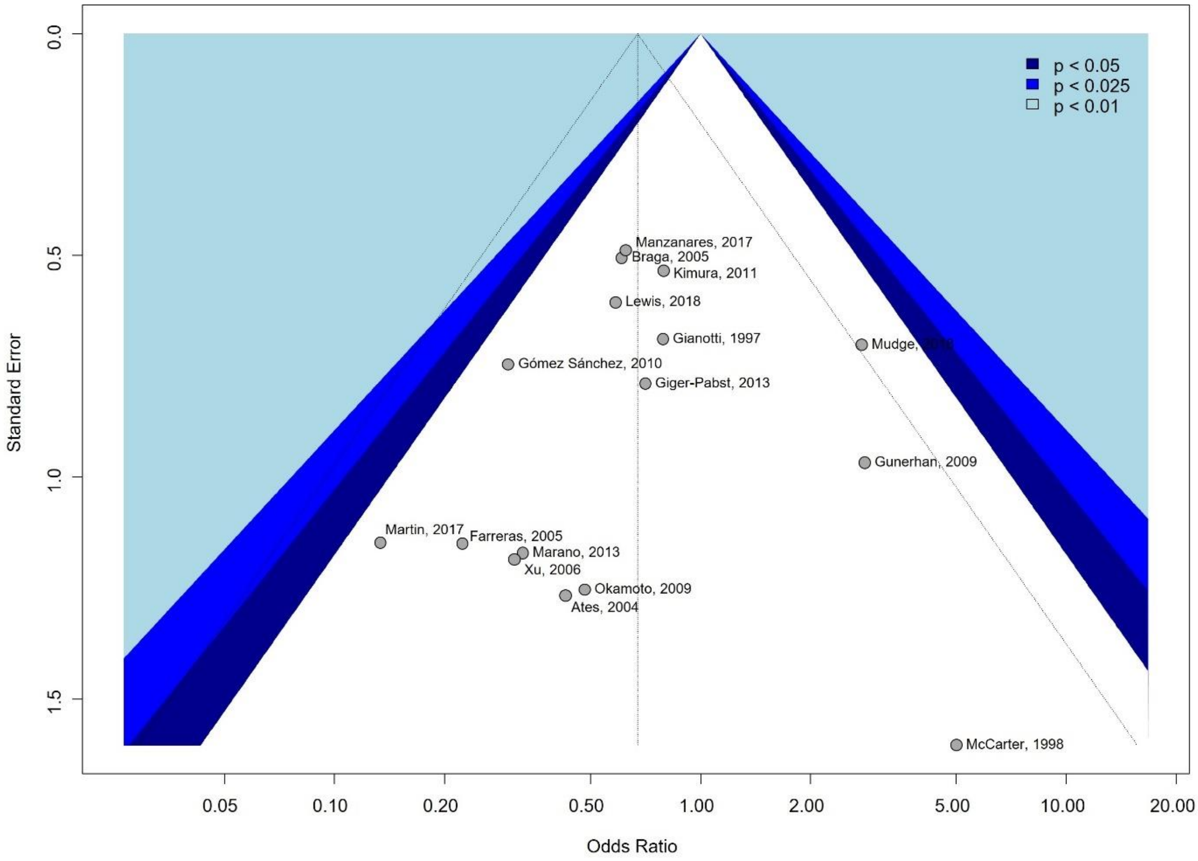

Egger test: t = 0.56, df = 14, p-value = 0.5818

Figure 43. Funnel plot: Analysis of the effect of preoperative immunonutrition support (arginine, nucleotides, omega-3 fatty acids) on wound infection in gastrointestinal cancer patients

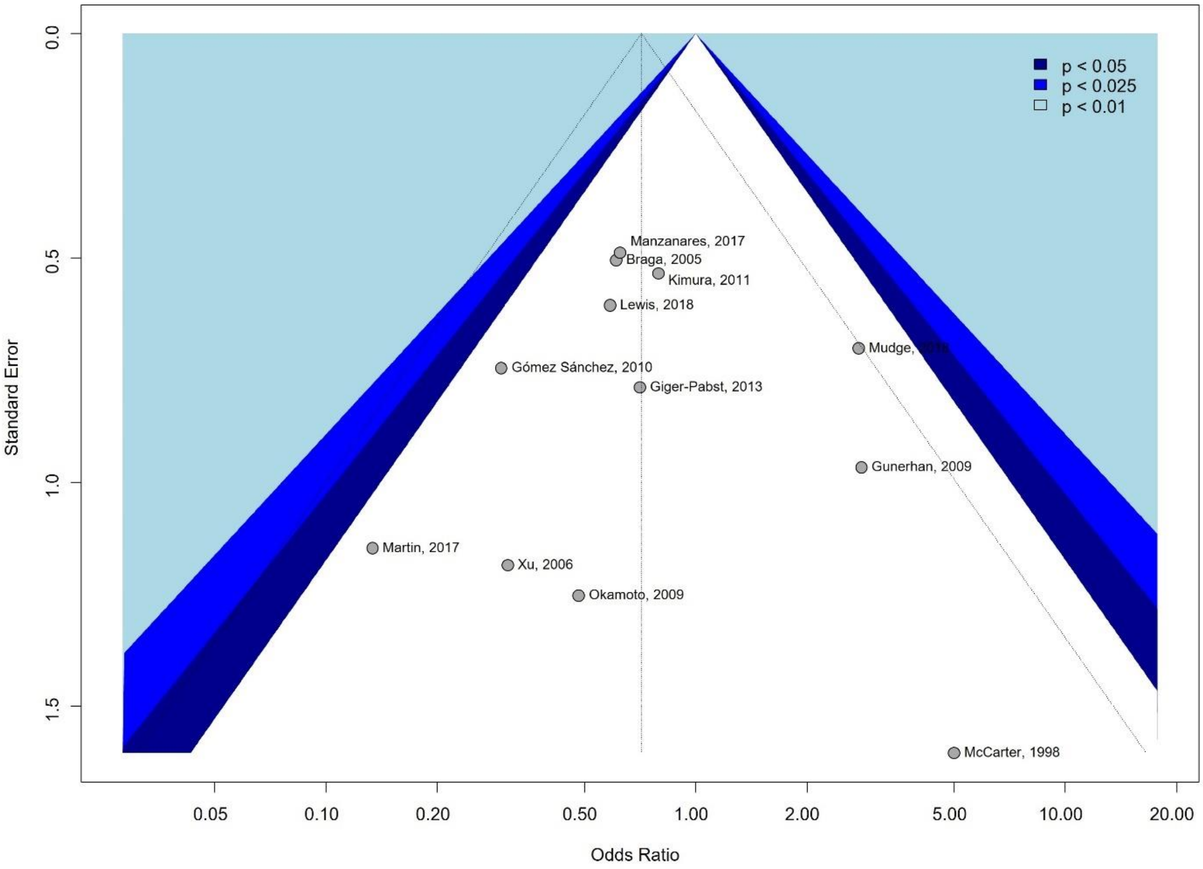

Egger test: t = 0.72, df = 10, p-value = 0.4897

Figure 44. Funnel plot: Analysis of the effect of perioperative immunonutrition support (arginine, nucleotides, omega-3 fatty acids) on non-infection complications in gastrointestinal cancer patients

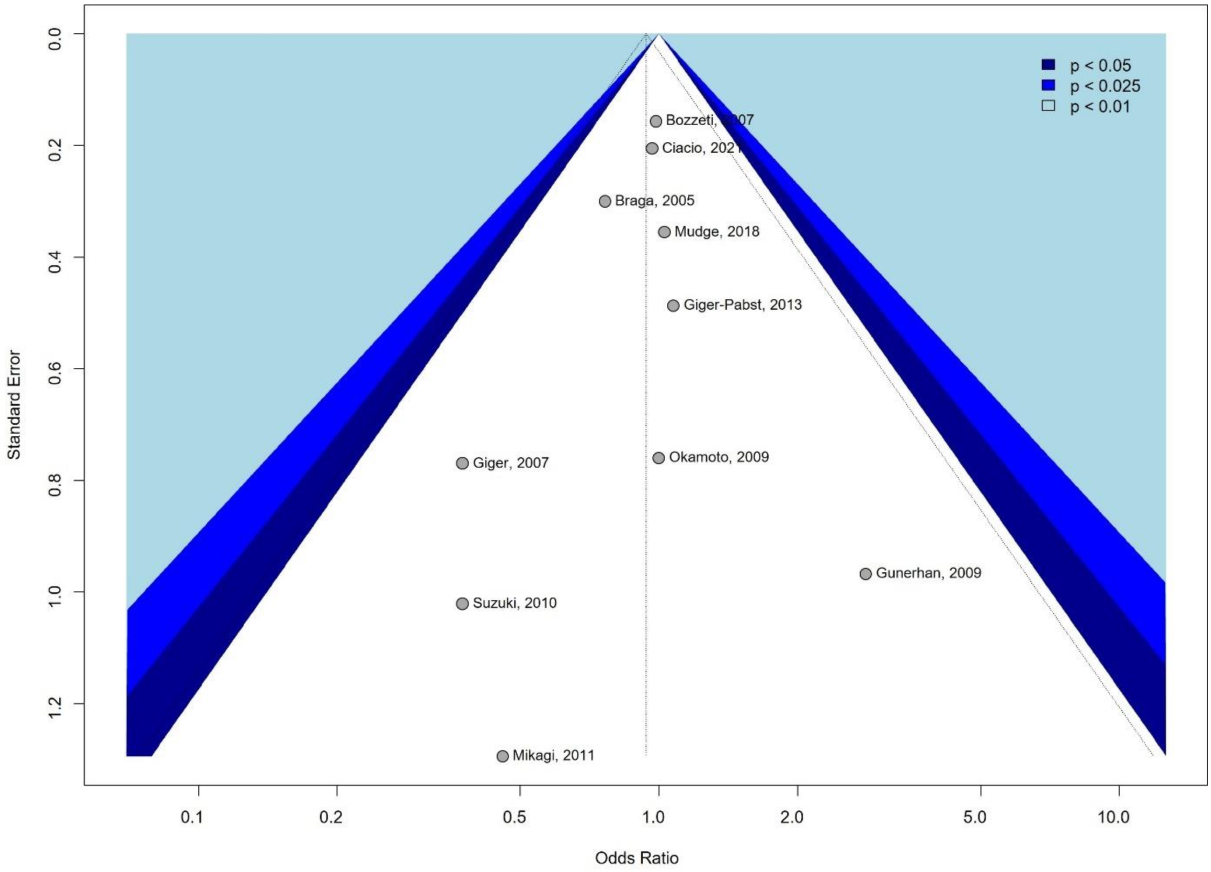

Egger test: t = -0.97, df = 8, p-value = 0.3623

Figure 45. Funnel plot: Analysis of the effect of perioperative immunonutrition support (arginine, nucleotides, omega-3 fatty acids) on the length of hospital stay in gastrointestinal cancer patients

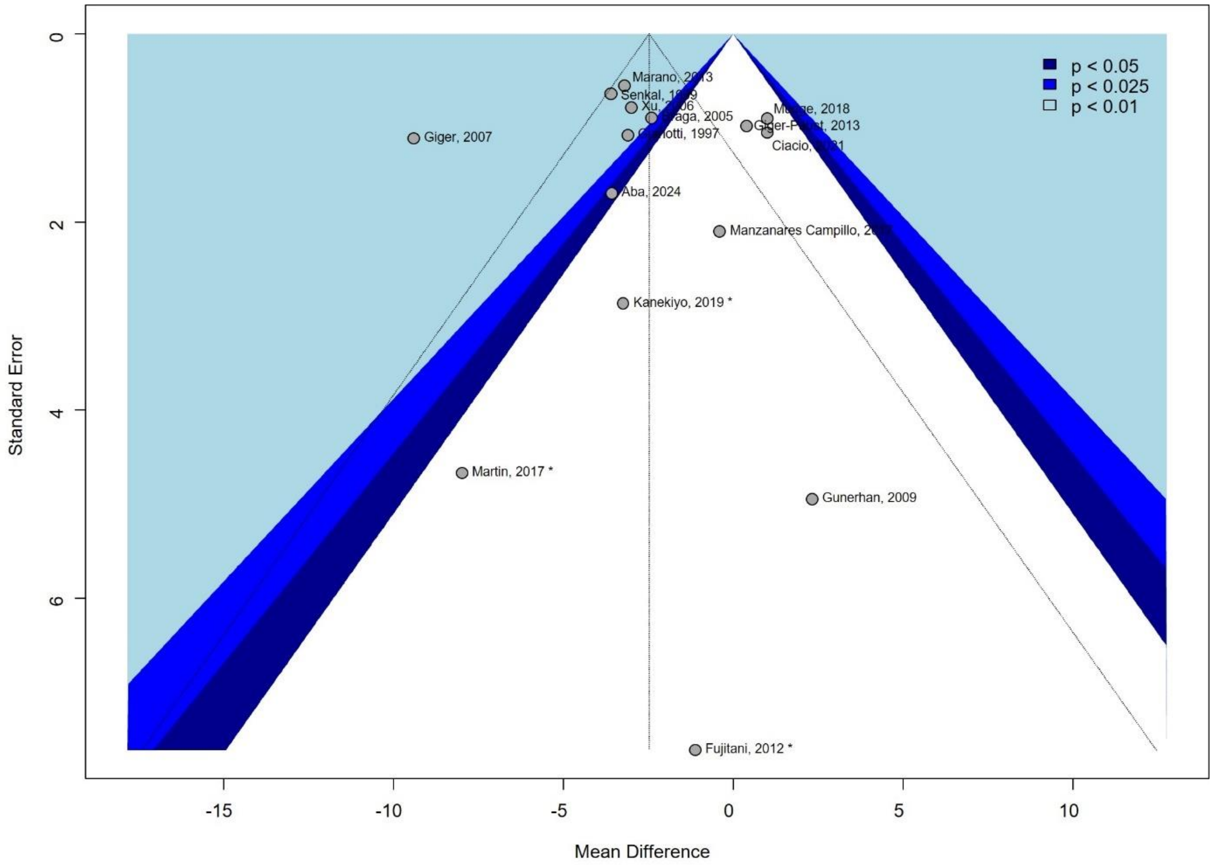

Egger test: t = 0.23, df = 13, p-value = 0.8194

Supplementary Figures 46-52: Leave-one-out analysis

We did leave-one-out analysis to assess the possible influence of articles on the overall results. Cases that are considered as possibly influential with respect to any of the shown measures are marked with a ‘star’ in column ‘Influential.’ Note that the chosen cut-offs are (somewhat) arbitrary based on dmetar package.

The provided column names are: ‘Influental’, ‘effect size’, ‘95% CI’, ‘I2’, ‘Std residual’, ‘Dffits’, ‘Cook’s dist.’, ‘Covariance ratio’, ‘Hat value’. The effect size is the pooled effect size without the given study. The 95% confidence interval of the pooled effect size without the given study. I^2 means the Higgins&Thomson I^2 heterogeneity value without the given study. Std residual is the studentized residual. It shows the deleted residual divided by its estimated standard deviation. Dffits is the difference in fits. It quantifies the number of standard deviations that the fitted value changes without the given study. (Typical threshold is $3*\sqrt{\frac{P}{k-P}}$). Cook’s dist means Cook’s distance. It depends on both the residual and leverage of the omitted study. (The typical threshold value is 2). Covariance ratio is the covariance ratio. It shows the change in the determinant of the covariance matrix of the effect size. (The typical threshold value is 1) Hat value means the value of the hat matrix without the given study. (Typical threshold is $3*\frac{P}{k}$)

Based on the leave-one-out analyses (LOO), we found influential articles in two cases, but did not find a relevant change in the effect (> 1 range in OR) or a relevant change in the decision on the null-hypothesis in the following cases.

Figure 46: Leave-one-out plot: Analysis of the effect of perioperative immunonutrition support (arginine, nucleotides, omega-3 fatty acids) on anastomotic leakage in gastrointestinal cancer patients

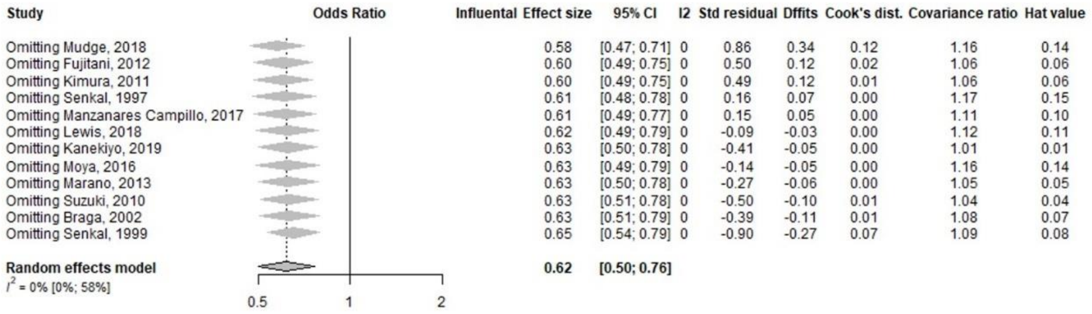

Study	Influental	Effect size	95% CI	I2	Std residual	Dffits	Cook's dist.	Covariance ratio	Hat value
Omitting Mudge, 2018		0.58	[0.47; 0.71]	0	0.86	0.34	0.12	1.16	0.14
Omitting Fujitani, 2012		0.60	[0.49; 0.75]	0	0.50	0.12	0.02	1.06	0.06
Omitting Kimura, 2011		0.60	[0.49; 0.75]	0	0.49	0.12	0.01	1.06	0.06
Omitting Senkal, 1997		0.61	[0.48; 0.78]	0	0.16	0.07	0.00	1.17	0.15
Omitting Manzanares Campillo, 2017		0.61	[0.49; 0.77]	0	0.15	0.05	0.00	1.11	0.10
Omitting Lewis, 2018		0.62	[0.49; 0.79]	0	-0.09	-0.03	0.00	1.12	0.11
Omitting Kanekiyo, 2019		0.63	[0.50; 0.78]	0	-0.41	-0.05	0.00	1.01	0.01
Omitting Moya, 2016		0.63	[0.49; 0.79]	0	-0.14	-0.05	0.00	1.16	0.14
Omitting Marano, 2013		0.63	[0.50; 0.78]	0	-0.27	-0.06	0.00	1.05	0.05
Omitting Suzuki, 2010		0.63	[0.51; 0.78]	0	-0.50	-0.10	0.01	1.04	0.04
Omitting Braga, 2002		0.63	[0.51; 0.79]	0	-0.39	-0.11	0.01	1.08	0.07
Omitting Senkal, 1999		0.65	[0.54; 0.79]	0	-0.90	-0.27	0.07	1.09	0.08
**Random effects model**		**0.62**	**[0.50; 0.76]**						

Figure 47: Leave-one-out plot: Analysis of the effect of perioperative immunonutrition support (arginine, nucleotides, omega-3 fatty acids) on infection complications in gastrointestinal cancer patients

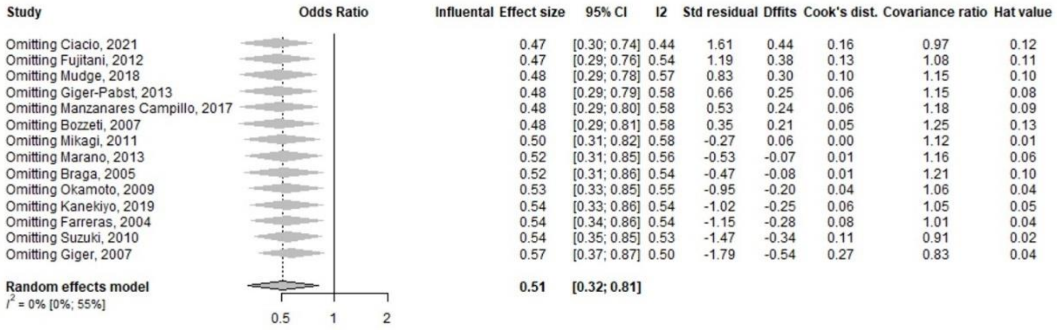

Figure 48: Leave-one-out plot: Analysis of the effect of perioperative immunonutrition support (arginine, nucleotides, omega-3 fatty acids) on urinary tract infection in gastrointestinal cancer patients

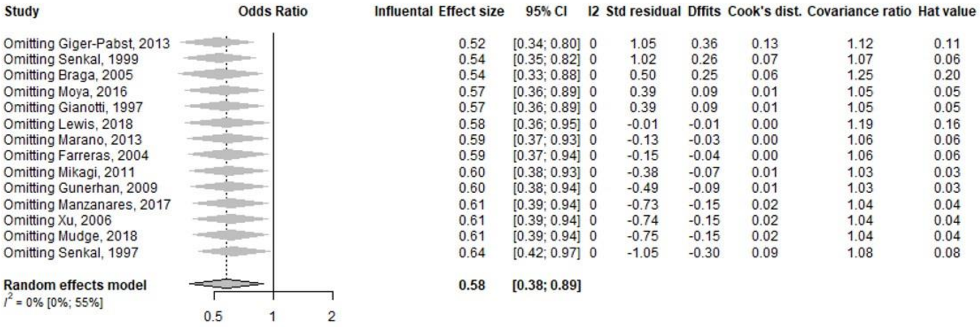

Figure 49: Leave-one-out plot: Analysis of the effect of perioperative immunonutrition support (arginine, nucleotides, omega-3 fatty acids) on wound infection in gastrointestinal cancer patients

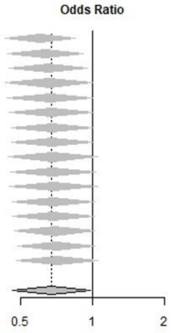

Study	Influental	Effect size	95% CI	I2	Std residual	Dffits	Cook's dist.	Covariance ratio	Hat value
Omitting Mudge, 2018		0.60	[0.43; 0.84]	0	2.09	0.59	0.35	1.08	0.07
Omitting Gunerhan, 2009		0.64	[0.44; 0.92]	0	1.51	0.30	0.09	1.04	0.04
Omitting McCarter, 1998		0.65	[0.45; 0.95]	0	1.26	0.15	0.02	1.01	0.01
Omitting Kimura, 2011		0.66	[0.43; 1.00]	0	0.33	0.13	0.02	1.15	0.13
Omitting Gianotti, 1997		0.66	[0.44; 1.00]	0	0.24	0.07	0.00	1.08	0.08
Omitting Giger-Pabst, 2013		0.67	[0.45; 1.01]	0	0.06	0.02	0.00	1.06	0.06
Omitting Okamoto, 2009		0.68	[0.45; 1.01]	0	-0.27	-0.04	0.00	1.02	0.02
Omitting Ates, 2004		0.68	[0.46; 1.01]	0	-0.36	-0.05	0.00	1.02	0.02
Omitting Manzanares, 2017		0.68	[0.44; 1.05]	0	-0.16	-0.07	0.00	1.18	0.15
Omitting Lewis, 2018		0.68	[0.45; 1.04]	0	-0.24	-0.08	0.01	1.11	0.10
Omitting Braga, 2005		0.68	[0.45; 1.05]	0	-0.21	-0.09	0.01	1.17	0.14
Omitting Marano, 2013		0.69	[0.46; 1.02]	0	-0.62	-0.10	0.01	1.03	0.03
Omitting Xu, 2006		0.69	[0.46; 1.02]	0	-0.66	-0.11	0.01	1.03	0.03
Omitting Farreras, 2004		0.69	[0.47; 1.02]	0	-0.97	-0.16	0.03	1.03	0.03
Omitting Martin, 2017		0.70	[0.49; 1.02]	0	-1.43	-0.24	0.06	1.03	0.03
Omitting Gómez Sánchez, 2009		0.71	[0.48; 1.05]	0	-1.13	-0.30	0.09	1.07	0.07
**Random effects model**		**0.67**	**[0.46; 0.98]**						
I^2 = 0% [0%; 52%]									

Figure 50: Leave-one-out plot: Analysis of the effect of preoperative immunonutrition support (arginine, nucleotides, omega-3 fatty acids) on wound infection in gastrointestinal cancer patients

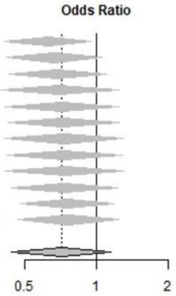

Study	Influental	Effect size	95% CI	I2	Std residual	Dffits	Cook's dist.	Covariance ratio	Hat value
Omitting Mudge, 2018		0.63	[0.41; 0.95]	0.00	2.02	0.63	0.36	0.99	0.09
Omitting Gunerhan, 2009		0.67	[0.42; 1.06]	0.00	1.45	0.32	0.10	0.94	0.05
Omitting McCarter, 1998		0.69	[0.43; 1.10]	0.01	1.22	0.16	0.03	0.93	0.02
Omitting Kimura, 2011		0.70	[0.40; 1.25]	0.13	0.17	0.06	0.00	1.38	0.15
Omitting Giger-Pabst, 2013		0.72	[0.41; 1.24]	0.14	-0.02	-0.02	0.00	1.29	0.07
Omitting Okamoto, 2009		0.73	[0.42; 1.24]	0.13	-0.31	-0.07	0.01	1.21	0.03
Omitting Manzanares, 2017		0.73	[0.41; 1.30]	0.13	-0.25	-0.12	0.02	1.40	0.17
Omitting Lewis, 2018		0.73	[0.42; 1.29]	0.13	-0.30	-0.12	0.02	1.33	0.12
Omitting Braga, 2005		0.74	[0.42; 1.30]	0.13	-0.29	-0.13	0.02	1.38	0.16
Omitting Xu, 2006		0.74	[0.44; 1.24]	0.10	-0.70	-0.15	0.02	1.13	0.03
Omitting Martin, 2017		0.75	[0.48; 1.19]	0.00	-1.48	-0.25	0.06	0.93	0.04
Omitting Gómez Sánchez, 2009		0.77	[0.47; 1.25]	0.01	-1.21	-0.34	0.11	1.00	0.08
**Random effects model**		**0.71**	**[0.44; 1.15]**						
I^2 = 0% [0%; 58%]									

Figure 51: Leave-one-out plot: Analysis of the effect of preoperative immunonutrition support (arginine, nucleotides, omega-3 fatty acids) on non-infection complications in gastrointestinal cancer patients

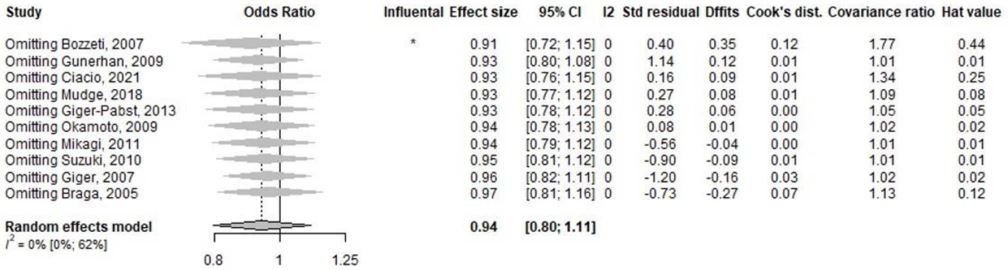

Study	Influental	Effect size	95% CI	I2	Std residual	Dffits	Cook's dist.	Covariance ratio	Hat value
Omitting Bozzeti, 2007	*	0.91	[0.72; 1.15]	0	0.40	0.35	0.12	1.77	0.44
Omitting Gunerhan, 2009		0.93	[0.80; 1.08]	0	1.14	0.12	0.01	1.01	0.01
Omitting Ciacio, 2021		0.93	[0.76; 1.15]	0	0.16	0.09	0.01	1.34	0.25
Omitting Mudge, 2018		0.93	[0.77; 1.12]	0	0.27	0.08	0.01	1.09	0.08
Omitting Giger-Pabst, 2013		0.93	[0.78; 1.12]	0	0.28	0.06	0.00	1.05	0.05
Omitting Okamoto, 2009		0.94	[0.78; 1.13]	0	0.08	0.01	0.00	1.02	0.02
Omitting Mikagi, 2011		0.94	[0.79; 1.12]	0	-0.56	-0.04	0.00	1.01	0.01
Omitting Suzuki, 2010		0.95	[0.81; 1.12]	0	-0.90	-0.09	0.01	1.01	0.01
Omitting Giger, 2007		0.96	[0.82; 1.11]	0	-1.20	-0.16	0.03	1.02	0.02
Omitting Braga, 2005		0.97	[0.81; 1.16]	0	-0.73	-0.27	0.07	1.13	0.12
**Random effects model**		**0.94**	**[0.80; 1.11]**						

I^2 = 0% [0%; 62%]

In case of "Analysis of the effect of preoperative immunonutrition (arginine, nucleotides, omega-3 fatty acids) on non-infection complications in gastrointestinal cancer patients," omitting Bozzeti 2007, the OR would be 0.91 (95% CI 0.72-1.15).

Figure 52: Leave-one-out plot: Analysis of the effect of perioperative immunonutrition support (arginine, nucleotides, omega-3 fatty acids) on length of hospital stay in gastrointestinal cancer patients

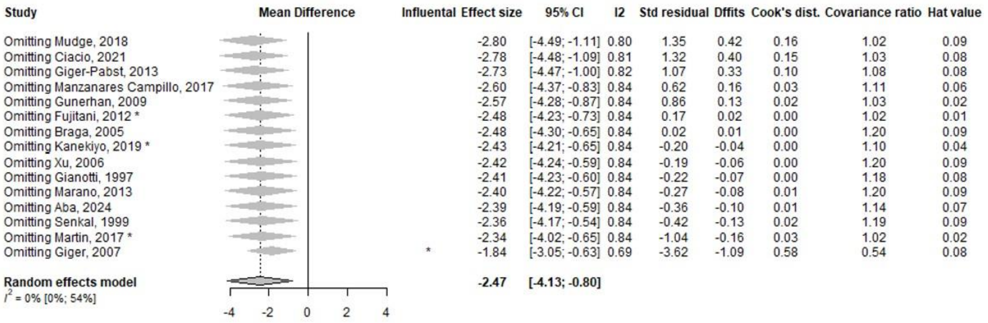

Study	Influental	Effect size	95% CI	I2	Std residual	Dffits	Cook's dist.	Covariance ratio	Hat value
Omitting Mudge, 2018		-2.80	[-4.49; -1.11]	0.80	1.35	0.42	0.16	1.02	0.09
Omitting Ciacio, 2021		-2.78	[-4.48; -1.09]	0.81	1.32	0.40	0.15	1.03	0.08
Omitting Giger-Pabst, 2013		-2.73	[-4.47; -1.00]	0.82	1.07	0.33	0.10	1.08	0.08
Omitting Manzanares Campillo, 2017		-2.60	[-4.37; -0.83]	0.84	0.62	0.16	0.03	1.11	0.06
Omitting Gunerhan, 2009		-2.57	[-4.28; -0.87]	0.84	0.86	0.13	0.02	1.03	0.02
Omitting Fujitani, 2012 *		-2.48	[-4.23; -0.73]	0.84	0.17	0.02	0.00	1.02	0.01
Omitting Braga, 2005		-2.48	[-4.30; -0.65]	0.84	0.02	0.01	0.00	1.20	0.09
Omitting Kanekiyo, 2019 *		-2.43	[-4.21; -0.65]	0.84	-0.20	-0.04	0.00	1.10	0.04
Omitting Xu, 2006		-2.42	[-4.24; -0.59]	0.84	-0.19	-0.06	0.00	1.20	0.09
Omitting Gianotti, 1997		-2.41	[-4.23; -0.60]	0.84	-0.22	-0.07	0.00	1.18	0.08
Omitting Marano, 2013		-2.40	[-4.22; -0.57]	0.84	-0.27	-0.08	0.01	1.20	0.09
Omitting Aba, 2024		-2.39	[-4.19; -0.59]	0.84	-0.36	-0.10	0.01	1.14	0.07
Omitting Senkal, 1999		-2.36	[-4.17; -0.54]	0.84	-0.42	-0.13	0.02	1.19	0.09
Omitting Martin, 2017 *		-2.34	[-4.02; -0.65]	0.84	-1.04	-0.16	0.03	1.02	0.02
Omitting Giger, 2007	*	-1.84	[-3.05; -0.63]	0.69	-3.62	-1.09	0.58	0.54	0.08
**Random effects model**		**-2.47**	**[-4.13; -0.80]**						

I^2 = 0% [0%; 54%]

In case of "Analysis of the effect of perioperative immunonutrition (arginine, nucleotides, omega-3 fatty acids) on length of hospital stay (days) in gastrointestinal cancer patients," omitting Giger 2007, the MD would be -1.84 (95% CI -3.05- -0.63).
